# Annulated Radical Cations with a C_4_E_2_‐Core (E = P, As, Sb): Stable Pnictogen Analogs of Elusive Aryl Radical Anions of Birch Reduction Reactions

**DOI:** 10.1002/anie.202505142

**Published:** 2025-05-19

**Authors:** Henric Steffenfauseweh, Yury V. Vishnevskiy, Beate Neumann, Hans‐Georg Stammler, Demi D. Snabilié, Bas de Bruin, Rajendra S. Ghadwal

**Affiliations:** ^1^ Molecular Inorganic Chemistry and Catalysis, Inorganic and Structural Chemistry Center for Molecular Materials Faculty of Chemistry Universität Bielefeld Universitätsstrasse 25 D‐33615 Bielefeld Germany; ^2^ University of Amsterdam (UvA), Faculty of Science, Van ’t Hoff Institute for Molecular Sciences (HIMS) Homogeneous and Supramolecular Catalysis Group Science Park 904 1098 XH Amsterdam The Netherlands

**Keywords:** Birch‐reduction, Heavy benzenes, Pnictogen, Radical cation

## Abstract

The benzene radical anion (C_6_H_6_)^●−^, possessing a 7π‐electron count, is a crucial intermediate in the Birch reduction and has been extensively studied both experimentally and theoretically. Herein, we report the tricyclic pnictogen radical cations [(ADC)E]_2_[B], [**2**‐E][B] (ADC = PhC{N(Dipp)C}_2_; Dipp = 2,6‐*i*Pr_2_C_6_H_3_; E = P, As, Sb; [B] = [B1] = B(C_6_F_5_)_4_ or [B2] = {3,5‐(CF_3_)_2_C_6_H_3_}) featuring a central 7π‐electron planar C_4_E_2_ ring embedded between two 1,3‐imidazole‐based anionic dicarbene (ADC) frameworks, as crystalline solids. [**2**‐E][B] are prepared by one‐electron (1e) oxidations of the corresponding base‐stabilized cyclic bis‐pnictinidene compounds [(ADC)E]_2_ (**1**‐E). Calculated spin densities (for E = P 76%, As 80%, Sb 88%) and EPR measurements suggest that [**2**‐E]^●+^ are pnictogen‐centered radicals and stabilized by the delocalization of the unpaired electron over the C_4_E_2_ ring. The EPR spectra of [**2**‐E][B] exhibit characteristic signals for *S* = ½ systems with clear hyperfine coupling constants for the two similar P, As, or Sb nuclei. Further 1e‐oxidations of [**2**‐E][B] result in the dicationic 6π‐electron C_4_E_2_‐species [(ADC)E]_2_[B]_2_ [**3**‐E][B]_2_, which readily undergo comproportionations with **1**‐E to afford [**2**‐E][B]. The radical reactivity of [**2**‐E][B2] is shown with 2,2,6,6‐tetramethylpiperidinyloxyl (TEMPO) and diphenyl diselenide (PhSeSePh) in yielding compounds [(ADC)_2_E{E(TEMPO)}][B2] (**4**‐E) (E = P, Sb) and [(ADC)_2_P{P(SePh)}][B2] (**5**‐P), respectively.

## Graphical abstract

One‐electron (1e‐) oxidations of the base‐stabilized cyclic bis‐pnictinidene compounds **1**‐E afford the 7π‐electron C_4_E_2_‐ring‐containing radical cations [**2**‐E]^●+^, which are valence isoelectronic to the benzene radical anion [C_6_H_6_]^●−^ of the Birch reduction reactions. Further, 1e‐oxidations of [**2**‐E]^●+^ give the dications [**3**‐E]^2+^, while reactions with TEMPO (or Ph_2_Se_2_) yield the corresponding diamagnetic monocations.

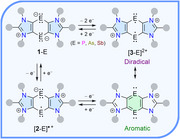

## Introduction

Stable radicals containing one or more unpaired electrons are highly sought‐after synthetic targets in modern organic^[^ ^1^, ^2^, ^3^ ^]^ and main‐group molecular chemistry.^[^ ^4^, ^5^, ^6^, ^7^, ^8^, ^9^, ^10^, ^11^, ^12^, ^13^, ^14^ ^]^ Their appeal stems primarily from their exceptional optical, magnetic, and electronic properties, which have significant implications for materials science.^[^ ^15^, ^16^, ^17^, ^18^, ^19^, ^20^, ^21^, ^22^, ^23^, ^24^, ^25^ ^]^ Additionally, these open‐shell species exhibit diverse and rich reactivity profiles, making them invaluable tools in synthetic chemistry.^[^ ^26^, ^27^, ^28^, ^29^, ^30^, ^31^ ^]^ Since Gomberg's isolation of the first stable radical (Ph_3_C) in 1900,^[^ ^32^ ^]^ numerous stable radicals have been reported.^[^ ^1^, ^2^, ^3^ ^]^ However, structurally characterized heavier main‐group radicals remain relatively scarce.^[^ ^4^, ^5^, ^6^, ^7^, ^8^, ^9^, ^10^, ^11^, ^12^, ^13^, ^14^, ^33^ ^]^ Among Group 15 elements,^[^ ^34^, ^35^ ^]^ the homolytic dissociation of dipnictanes (R₂E─ER₂; E = P or As) into their corresponding radicals (R₂E) in solution was first described by Lappert in 1976.^[^ ^36^ ^]^ In 2007, Cummins and coworkers isolated a monomeric neutral radical (R_2_P) (R = NV(N(Ar)CH_2_ *t*Bu; Ar = 3,5‐Me_2_C_6_H_3_), stabilized by the vanadium(IV/V) redox couple.^[^ ^37^ ^]^ Subsequently, Bertrand et al. reported a phosphinyl radical cation supported by a cyclic alkyl amino carbene (cAAC) Lewis base.^[^ ^38^, ^39^ ^]^

**Figure 1:**
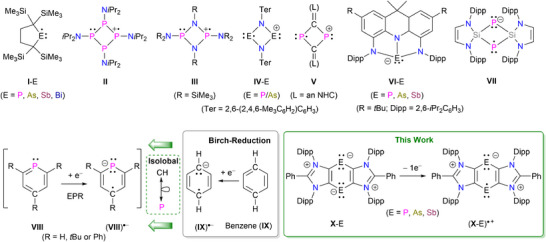
Selected examples of structurally characterized cyclic pnictogen‐containing radical species **I**‐E–**VII**. A schematic illustration of the Birch reduction of benzene (**IX**) to the radical anion (**IX**)^●−^ (and phosphabenzene (**VIII**) to (**VIII**)^●−^) and valence isoelectronic pnictogen radical cations (**X**‐E)^●+^.

In 2011, Iwamoto and coworkers synthesized the first metal‐free, neutral cyclic C_4_P‐radical **I**‐P (Figure 1).^[^ ^40^ ^]^ The same research group later succeeded in isolating its heavier analogues, **I**‐E (E = As, Sb, Bi.^[^ ^41^, ^42^ ^]^ In 2014, Wang and coworkers reported the four‐membered cyclic radical cations **II** and **III**, in which the spin density primarily resides on the exocyclic nitrogen atoms.^[^ ^43^ ^]^ The following year, Schulz and coworkers prepared delocalized radical cations **IV**‐E^[^ ^44^, ^45^ ^]^ through one‐electron (1e) oxidations of the corresponding singlet diradicaloids featuring a 6π‐electron N_2_E_2_‐ring.^[^ ^46^ ^]^ In **IV**‐E, the spin density was found to be almost entirely localized on the pnictogen atoms of the N_2_E_2_ ring. Grützmacher, Li, and colleagues reported related carbon derivatives,^[^ ^47^, ^48^ ^]^ such as **V**, obtained via 1e‐oxidation of an N‐heterocyclic carbene (NHC)‐based 6π‐electron C_2_P_2_ diradicaloid.^[^ ^49^ ^]^ In 2019, Wang and coworkers isolated tri‐coordinated, non‐trigonal pnictogen‐centered radical anions **VI**‐E, based on an NNN‐pincer ligand.^[^ ^50^ ^]^ The same research group also reported two radical cations featuring a Ga_2_E_2_ ring (E = P or As).^[^ ^51^ ^]^ Most recently, Liu's research group adopted a different approach and successfully isolated a Si_2_P_2_‐radical anion **VII**, characterized by a unique 2‐center‐3‐electron π‐bond.^[^ ^52^ ^]^

The formation of the radical cations **IV**‐E^[^ ^44^, ^45^ ^]^ and **V** ^[^ ^47^ ^]^ through the 1e‐oxidation of their corresponding E_2_N_2_‐ and P_2_C_2_‐diradicaloid, both Hückel 6π‐electron aromatic systems, is remarkable. Notably, Ashe III, Maerkl, and colleagues demonstrated as early as 1974 the viability of phosphabenzene radical anions (**VIII**)^●−^, generated via 1e‐reduction of **VIII** with potassium, as evidenced by EPR spectroscopy.^[^ ^53^ ^]^ The species (**VIII**)^●−^ can be considered phosphorus analogs of aryl radical anions of Birch reduction reactions,^[^ ^54^, ^55^ ^]^ such as (**IX**)^●−^, which serve as intermediates in organic synthesis.^[^ ^56^, ^57^, ^58^, ^59^, ^60^, ^61^, ^62^ ^]^ We recently reported tricyclic compounds **X**‐E, which feature a central C_4_E_2_ ring with two‐coordinated pnictogen E(I) atoms.^[^ ^63^, ^64^, ^65^ ^]^ Each pnictogen atom in **X**‐E possesses two lone pairs: one in the σ‐type (ns) orbital and the other in the π‐type (np) orbital. Consequently, **X**‐E can be considered Lewis base‐stabilized cyclic bis‐pnictinidene species.^[^ ^66^, ^67^ ^]^ These compounds undergo 2e‐oxidations, yielding the corresponding dications (**X**‐E)^2+^ with a 6π‐electron aromatic C_4_E_2_ core. Building on these findings,^[^ ^68^, ^69^, ^70^ ^]^ we assumed that the radical cations (**X**‐E)^●+^, which are valence isoelectronic to both aryl anions (**IX**)^●−^ of Birch reduction reactions^[^ ^54^, ^55^ ^]^ and transient phosphabenzene radical anions (**VIII**)^●−^, should be synthetically accessible via 1e‐oxidation of **X**‐E. Herein, we report the synthesis of radical cations (**X**‐E)^●+^ as crystalline solids and explore their structure and reactivity.

## Results and Discussions

**Figure 2:**
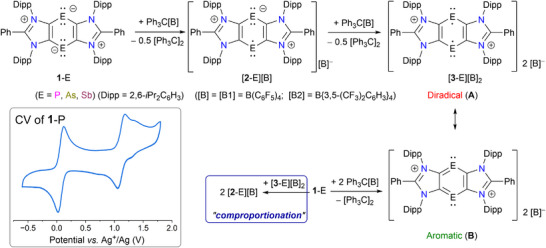
Sequential 1e‐oxidations of **1**‐E to [**2**‐E][B] and [**3**‐E][B]_2_ (represented by diradical **A** and aromatic **B** resonance forms). Comproportionation of **1**‐E and [**3**‐E][B]_2_ to [**2**‐E][B]. Inset: CV of compound **1**‐P (in *o*‐difluorobenzene, 0.1 m *n*Bu_4_N[PF_6_], 100 mV s^−1^: see the Supporting Information for details).

We began our studies with electrochemical investigations of the starting compounds **1**‐P, **1**‐As, and **1**‐Sb, which were synthesized as previously reported.^[^ ^63^, ^64^, ^65^ ^]^ The cyclic voltammogram (CV) of **1**‐P (Figure 2 inset) exhibits two main reversible redox events at *E*_1/2 _= 0.07 and 1.12 V, which may be tentatively assigned to the [**1**‐P] ⇌ [**1**‐P]^●+^ and [**1**‐P]^●+^ ⇌ [**1**‐P]^2+^ redox couples, respectively. For **1**‐As and **1**‐Sb, the first reversible redox wave appears at *E*_1/2_ = 0.08 and 0.10 V, respectively, while the second (quasi/irreversible) wave is located in the 1.10 to 1.19 V region (see Figures S39 and S40). The dicationic species [**1**‐E]^2+^ (E = P, As), featuring a 6π‐electron C_4_E_2_ ring, are closed‐shell compounds and have been previously isolated as stable crystalline solids with chloride or triflate counter anions.^[^ ^63^, ^64^ ^]^

Treating a benzene solution of **1**‐E with one equivalent of Ph_3_C[B] ([B] = [B1] = B(C_6_F_5_)_4_) or [B2] = B{3,5‐(CF_3_)_2_C_6_H_3_}_4_) at room temperature induced an immediate color change (brown to blue for **1**‐P, brown to turquoise for **1**‐As, and wine‐red to green for **1**‐Sb) and led to the precipitation of [**2**‐E][B]. The resulting compounds [**2**‐P][B] (blue), [**2**‐As][B] (turquoise), and [**2**‐Sb][B] (green) were isolated as colored solids in 48%–85% yields. Further reaction of [**2**‐P][B1] and [**2**‐As][B1] with Ph_3_C[B1] led to the formation of the new dicationic compounds [**3**‐P][B1]_2_ and [**3**‐As][B1]_2_, respectively. The [**3**‐E]^2+^ species can be represented by diradical (**A**) and aromatic (**B**) resonance forms, as discussed previously with different counter anions.^[^ ^63^, ^64^, ^65^ ^]^ Similarly, the unpaired electron in [**2**‐E][B] may delocalize over the central C_4_E_2_ ring, contributing to their stability. Compounds [**2**‐E][B] can also be obtained in near‐quantitative yields via the comproportionation of **1**‐E and [**3**‐E][B]_2_. Compounds [**2**‐E][B] are NMR silent and exhibit a doublet EPR signal, consistent with *S* = ½ spin systems (see below). As expected, [**3**‐E][B1]_2_ (E = P or As) are closed‐shell species, displaying well‐resolved NMR spectra (see the Supporting Information).

**Figure 3:**
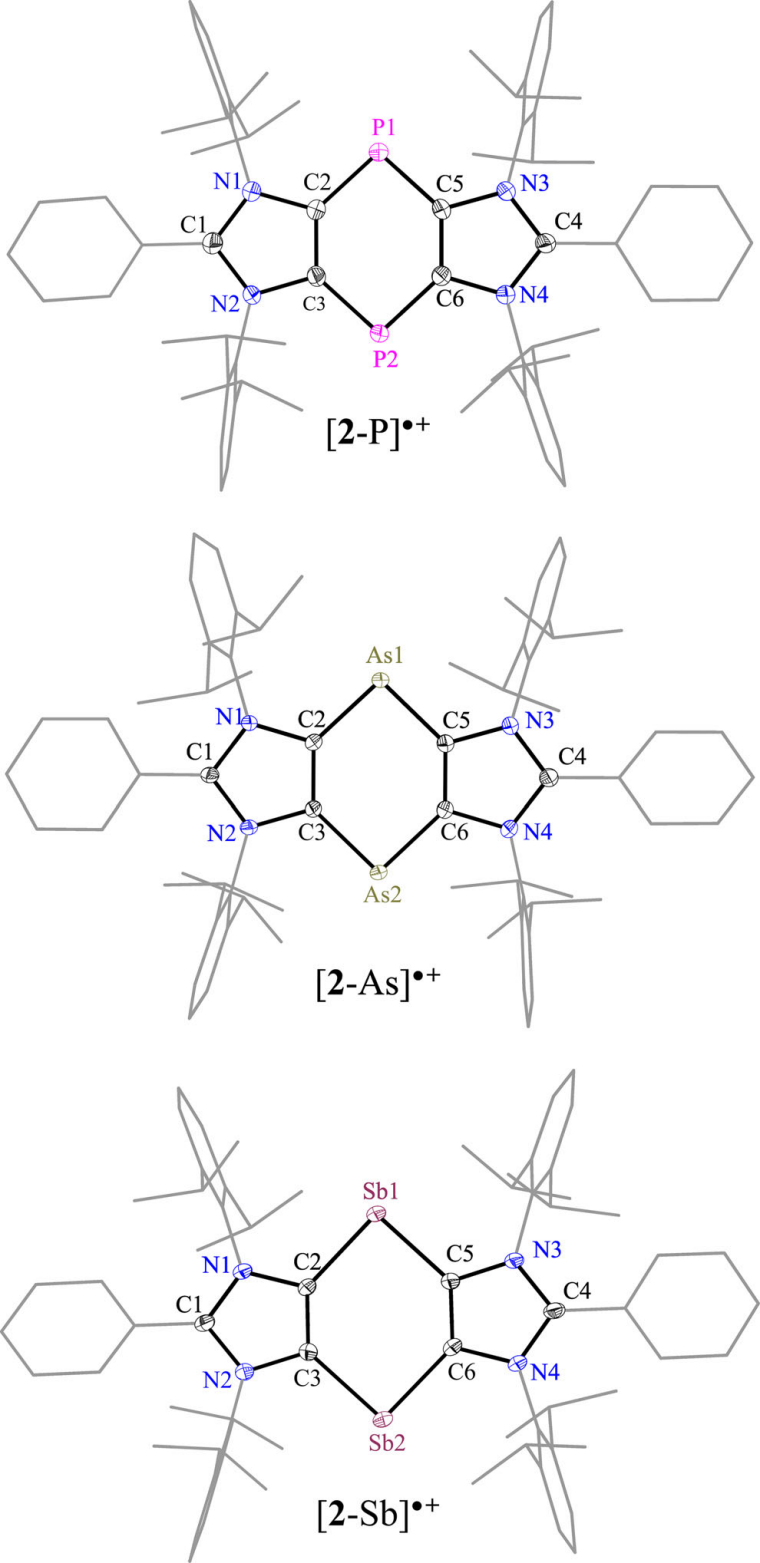
Solid‐state molecular structure of the radical cations [**2**‐E]^●+^ (E = P, As, Sb). For clarity, hydrogen atoms, minor disordered components, solvent molecules, and counter anions are omitted, while Dipp groups are depicted as wireframes. Thermal ellipsoids are drawn at 50% probability.

The solid‐state molecular structures of [**2**‐E][B] (Figure 3),^[^ ^71^ ^]^ determined by single‐crystal X‐ray diffraction (sc‐XRD), reveal the expected atom connectivity for the cationic part [**2**‐E]^●+^ and feature a borate counter anion [B1] or [B2]. The C_4_E_2_ ring of [**2**‐E]^●+^ is nearly planar, with two‐coordinated pnictogen atoms. The C─E bond lengths in [**2**‐E]^●+^ are intermediate between those of the corresponding neutral (**1**‐E) and dicationic [**3**‐E]^2+^ compounds (Table 1). As in [**3**‐E]^2+^ (see aromatic form **B** in Figure 2), this suggests delocalization of the unpaired electron over the C_4_E_2_ ring. Therefore, [**2**‐E]^●+^ can be formally regarded as isovalent electronic species to elusive aryl anions (e.g., (**IX**)^●−^) of Birch reduction reactions,^[^ ^54^, ^55^ ^]^ which contain 7π electrons in the six‐membered ring.

**Table 1:** Selected bond lengths (Å) and angles (°) for the C_4_E_2_ ring of **1**‐E, [**2**‐E]^●+^, and [**3**‐E]^2+^.^a)^

	C–E	C–C	C–C–E	C–E–C
**1**‐P	1.788(1)‐1.794(1)	1.393(2)‐1.395(2)	131.8(1)‐132.3(1)	94.6(1)‐95.1(1)
[**2**‐P]^●+^ [calculated]^b)^	1.771(2)‐1.779(2) [1.770]	1.391(2)‐1.393(2) [1.394]	132.2(1)‐133.3(1) [132.7]	94.4(1)‐95.0(1) [94.4]
[**3**‐P]^2+^	1.747(2)‐1.748(2)	1.398(3)	125.0(3)‐125.1(3)	96.0(2)
**1**‐As	1.918(2)‐1.923(2)	1.383(2)‐1.384(4)	132.9(2)‐133.8(1)	92.6(1)‐93.2(1)
[**2**‐As]^●+^ [calculated]^b)^	1.896(2)‐1.910(2) [1.900]	1.383(3)‐1.388(3) [1.387]	133.2(2)‐133.7(2) [133.6]	92.4(1)‐92.8(1) [92.7]
[**3**‐As]^2+^	1.856(5)‐1.877(5)	1.395(7)‐1.398(7)	132.0(4)‐133.6(4)	93.3(2)‐94.5(2)
**1**‐Sb	2.139(2)‐2.142(2)	1.385(2)‐1.386(2)	134.2(2)	90.8(1)
[**2**‐Sb]^●+^ [calculated]^b)^	2.115(2)‐2.127(3) [2.112]	1.381(3)‐1.387(3) [1.384]	133.3(2)‐135.1(2) [134.5]	90.4(1)‐91.2(1) [90.4]
[**3**‐Sb]^2+^	2.088(2)‐2.094(2)	1.390(3)	132.3(2)	91.5(1)

The values of only one molecule are given from two molecules in the asymmetric unit of [**2**‐E][B1].

^a)^ Taken from references [63, 64, 65] for **1**‐E and [**3**‐E]^2+^.

^b)^ At PBE0‐D3BJ/def2‐TZVPP.

The C–E bond lengths in [**2**‐E]^●+^ [E = P: 1.771(2)–1.779(2) Å, As: 1.896(2)–1.910(2) Å, Sb: 2.115(2)–2.127(3) Å] increase with the size of the pnictogen atom, while the C─E─C bond angle becomes more acute [E = P: 94.4(1)–95.0(1)°, As: 92.4(1)–92.8(1)°, Sb: 90.4(1)–91.2(1)°]. Moreover, the C─E bond lengths in [**2**‐E]^●+^ are shorter than those of the localized pnictogen radicals **I**‐E (P: 1.869(1) Å; As: 2.006(2)‐2.007(2) Å; Sb: 2.252(2)‐2.253(2) Å).^[^ ^40^, ^41^, ^42^ ^]^ The C─P bond lengths in [**2**‐E]^●+^ are slightly larger than that in a phosphaalkene radical anion (1.757(3) Å)^[^ ^72^ ^]^ but closely resemble those in the delocalized C_2_P_2_‐radical cation (1.771(3)‐1.772(3) Å).^[^ ^47^ ^]^ Additionally, the C─C bond lengths in [**2**‐E]^●+^ align with those observed in the dicationic aromatic systems [**3**‐E]^2+^ (Table 1). Collectively, these structural features suggest considerable delocalization of the unpaired electron over the C_4_E_2_ ring in [**2**‐E]^●+^, as further supported by EPR spectroscopy and computational studies (see below).

**Figure 4:**
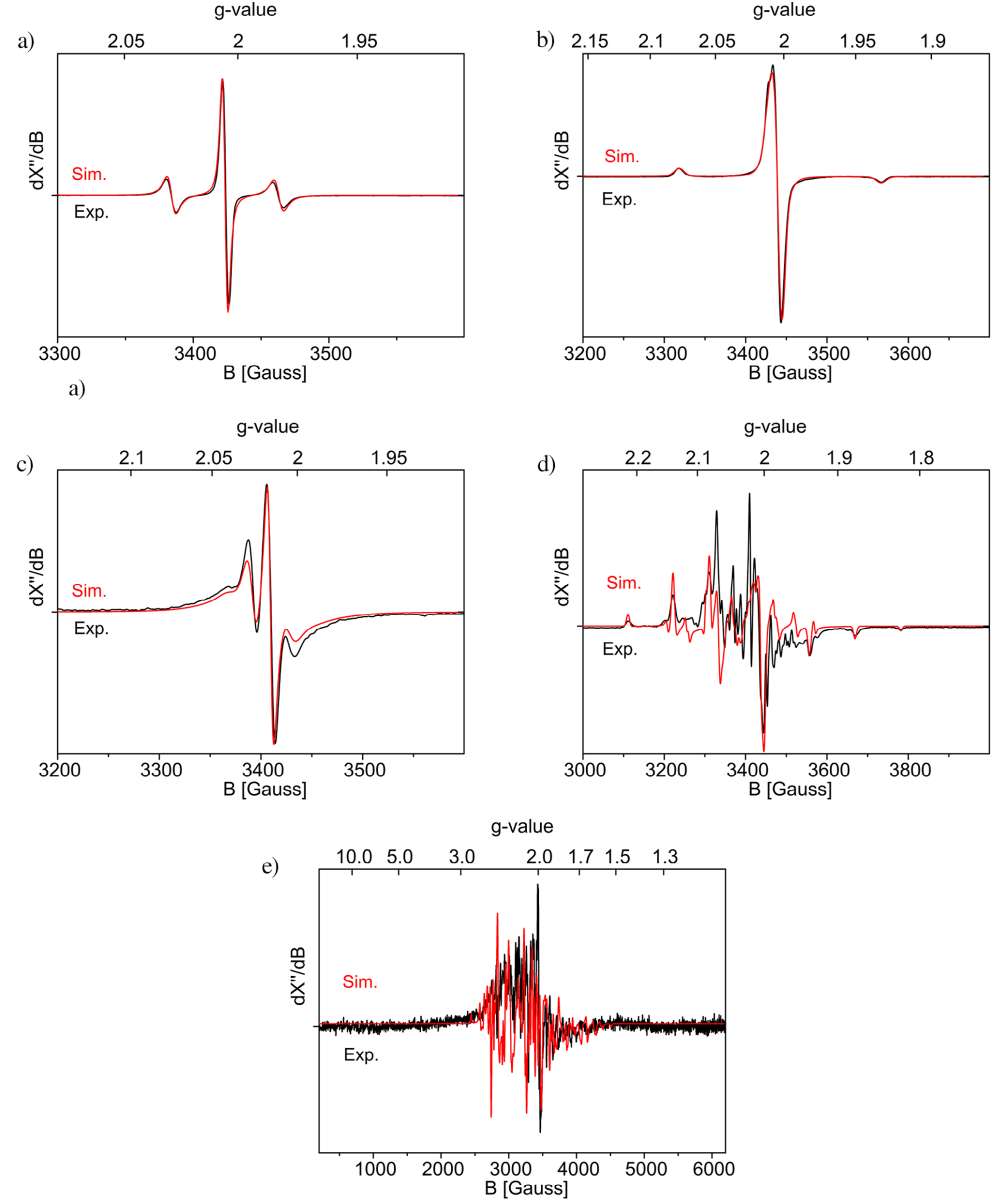
a) Isotropic X‐band EPR spectrum of [**2**‐P][B1] (at 298 K in Me‐THF). Mw. Freq = 9.6094 GHz, Mod. Amp 4 G. Mw. Power = 1 mW. b) X‐band EPR spectrum of [**2**‐P][B1] (at 20 K in Me‐THF). Mw. Freq = 9.6491 GHz, Mod. Amp 4 G. Mw. Power = 2 mW. c) Isotropic X‐band EPR spectrum of [**2**‐As][B1] (at 298 K in Me‐THF). Mw. Freq = 9.6094 GHz, Mod. Amp 4 G. Mw. Power = 1 mW. d) X‐band EPR spectrum of [**2**‐As][B1] (at 20 K in Me‐THF). Mw. Freq = 9.6451 GHz, Mod. Amp 4 G. Mw. Power = 2 mW. e) X‐band EPR spectrum of [**2**‐Sb][B1] (at 20 K in Me‐THF). Mw. Freq = 9.6491 GHz, Mod. Amp 4 G. Mw. Power = 20 mW.^[^ ^81^ ^]^ Applied simulation parameters are shown in Table 2 and Figures S41–S43.

The EPR spectra of [**2**‐P][B1], [**2**‐As][B1], and [**2**‐Sb][B1] (Figure 4 and Table 2) exhibit characteristic signals for *S* = ½ systems, with clear hyperfine coupling constants (HFCs) with two equivalent P, As, and Sb nuclei, respectively. The spectra do not show other resolved HFCs. The EPR spectrum of [**2**‐P][B1] shows three lines due to the coupling of the unpaired electron with two magnetically equivalent phosphorus ^31^P nuclei (*I* = ½). The magnitude of the HFC *A*_iso_(^31^P) in [**2**‐P][B1] amounts to 111 MHz (∼39.6 G), which is substantially larger than in the diphosphene radical cations [{(NHC)C(Ph)}P}_2_]^●+^ (NHC = IPr = {CHN(Dipp)}_2_C, 12 G, or SIPr = {CH_2_N(Dipp)}_2_C, 20 G)^[^ ^73^ ^]^ but smaller than in [(μ‐NTer)_2_P_2_]^●+^ (Ter = 2,6‐dimesitylphenyl, 55 G).^[^ ^44^ ^]^ The anisotropic spectrum recorded in frozen Me‐THF reveals rhombic, near axial *g*‐ and *A*‐tensors with large P‐HFCs in one direction to two equivalent P‐nuclei. The EPR spectra of [**2**‐As][B1] and [**2**‐Sb][B1] also reveal hyperfine coupling to two equivalent arsenic ^75^As nuclei (*I* = 3/2) and antimony nuclei [^121^Sb: *I* = 5/2 (57%) and ^123^Sb: *I* = 7/2 (43%)]. The observed EPR features of compound [**2**‐As][B1] match with those of the reported diarsene radical cations^[^ ^74^, ^75^, ^76^ ^]^ and the cyclic [(μ‐NTer)_2_As_2_]^●+^ radical cation.^[^ ^44^ ^]^ Expectedly, the hyperfine couplings increase for the heavier As and Sb nuclei compared to phosphorus. Furthermore, the anisotropy of the *A*‐tensor decreases from P via As to Sb with quite large HFCs to Sb in all three directions for [**2**‐Sb][B1]. At 298 K, compound [**2**‐Sb][B1] exhibits a featureless EPR signal, while at 20 K it shows a highly complex set of lines. A similar trend was also seen with literature known Sb‐radical‐cations.^[^ ^77^, ^78^, ^79^, ^80^ ^]^ Compounds [**2**‐E][B1] reveal restricted tumbling in solution, requiring simulation of the solution spectra with a restricted tumbling model parameter t_corr_ of about 1.3e^−10^, leaving the anisotropy of the *g*‐ and *A*‐tensors (partially) intact in the “garlic” EasySpin simulations.

**Table 2 anie202505142-tbl-0002:** Experimental (spectral simulation)^a)^ and DFT calculated^b)^ EPR parameters of [**2**‐P][B1], [**2**‐As][B1] and [**2**‐Sb][B1].

	Frozen Solution (20 K)		RT Solution (298 K)		
[**2**‐P][B1]	*g*‐tensor	*A*‐tensor^c)^	*g*‐tensor	*A*‐tensor^c)^	*t*_corr._ ^d)^
Exp.	2.010 2.0025 2.0025	24 (NR) −37 (NR) +348	2.009 2.004 2.003	−10 (NR) −5 (NR) +348	1.1 × 10^−10^
DFT	2.008 2.002 2.001	−24 −36 +318			
[**2**‐As][B1]	*g*‐tensor	*A*‐tensor^c)^	*g*‐tensor	*A*‐tensor^c)^	*t*_corr._ ^d)^
Exp.	2.047 2.005 1.990	−149 −127 +313	2.042 2.005 1.990	−75 (NR) −75 (NR) +300	1.35 × 10^−10^
DFT	2.037 2.003 1.997	−148 −129 +233			
[**2**‐Sb][B1]	*g*‐tensor	*A*‐tensor^c)^	*g*‐tensor	*A*‐tensor^c)^	*t*_corr._ ^d)^
Exp.	2.070 1.970 1.965	−390 +490 −550	−	−	−
DFT	2.073 1.972 1.967	−396 +492 −573			

^a)^ EasySpin 6.0.6 (cwEPR 3.6.0 plugin). For the applied (anisotropic) line broadening parameters (lwpp and H‐strain), see Figures S41–S43 NR = not resolved.

^b)^ Turbomole, b3‐lyp, x2c‐TZVPPall‐2c, relativistic two‐component calculations.

^c)^ Hyperfine couplings in MHz with two equivalent P, As, or Sb nuclei. The signs are based on the DFT results (and needed in the averaging of values for the solution spectra).

^d)^ Restricted tumbling of the molecule in solution: rotational correlation time constant.

**Figure 5:**
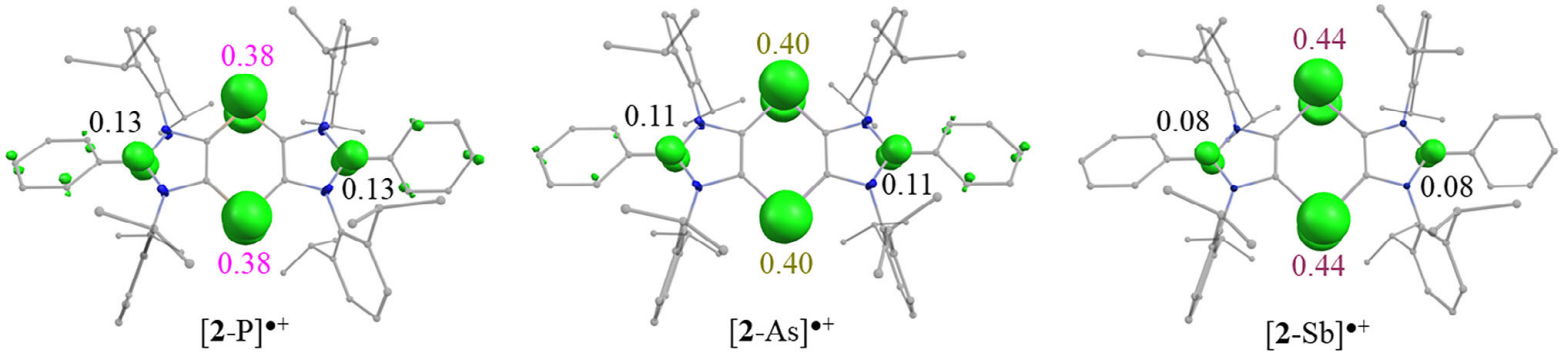
Calculated (PBE0‐D3BJ/def2‐TZVPP) spin density plots (isosurfaces 0.005 a.e.) for [**2**‐E]^●+^.

**Figure 6:**
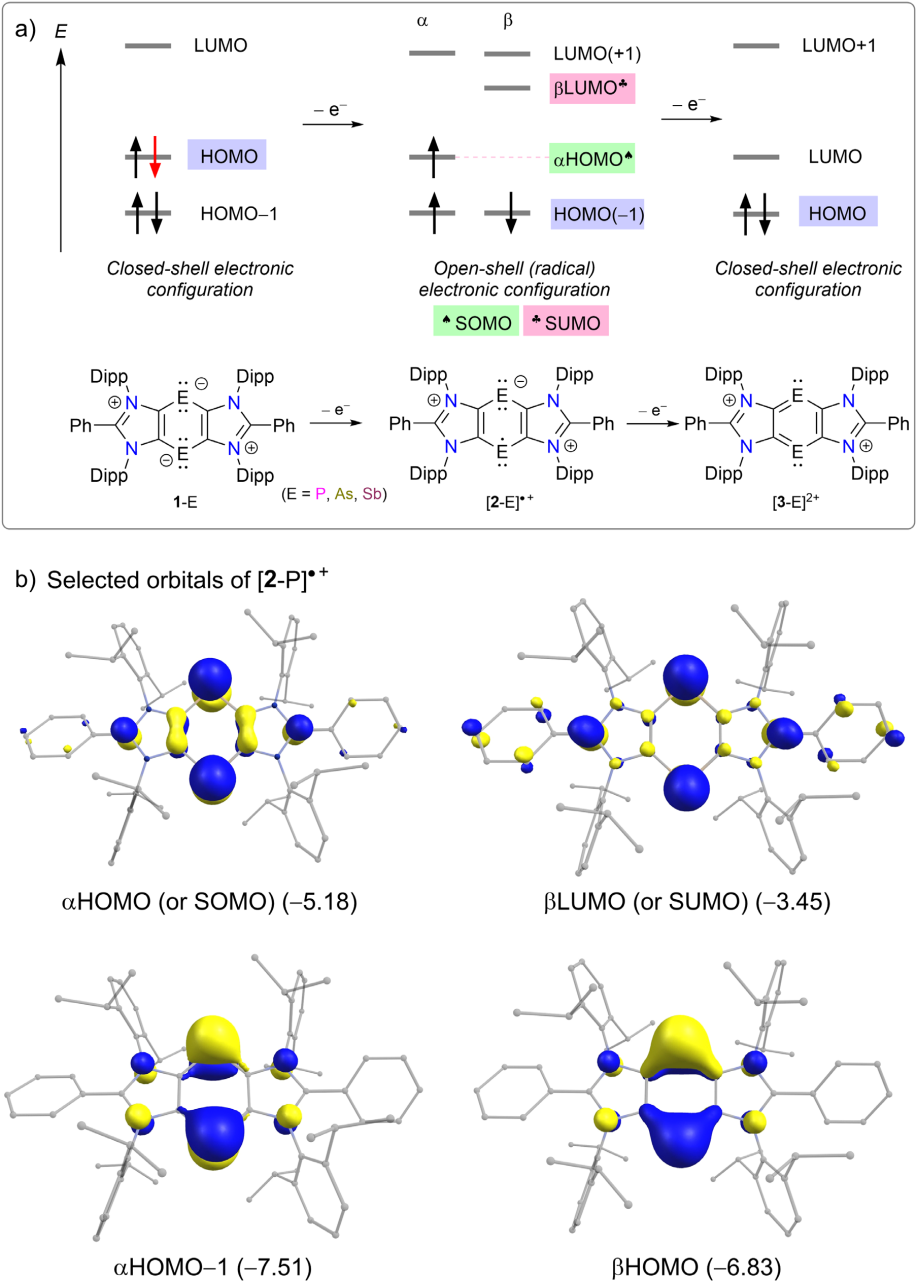
a) Simplified illustration of the frontier orbitals of **1**‐E, [**2**‐E]^●+^, and [**3**‐E]^2+^. b) Selected molecular orbitals (isosurfaces 0.05 a.u.) of [**2**‐P]^●+^ and their energies (in eV) calculated at PBE0/def2‐TZVPP (see the Supporting Information for [**2**‐As]^●+^ and [**2**‐Sb]^●+^).

To obtain[Table anie202505142-tbl-0002]further insights into the electronic structures of [**2**‐E][B], we performed quantum chemical calculations using the optimized structures (at PBE0‐D3BJ/def2‐TZVPP) of the corresponding radical cations, i.e., [**2**‐E]^●+^ (see the Supporting Information for details). The DFT‐optimized structures of [**2**‐P]^●+^ (Figure S33), [**2**‐As]^●+^ (Figure S34), and [**2**‐Sb]^●+^ (Figure S35) are in good qualitative agreement with those of the sc‐XRD structures (Table 1, Figure 3). According to NBO (natural bond orbital) analyses, the natural atomic charge (Table S4) on the pnictogen atoms of [**2**‐E]^●+^ (E = P, 0.46; As, 0.49; Sb, 0.62) is intermediate of those of the neutral **1**‐E and dicationic species [**3**‐E]^2+^.^[^ ^63^, ^64^, ^65^ ^]^ The carbon atoms of the C_4_E_2_ ring of [**2**‐E]^●+^ bear negative charges (for C_P_, −0.18; C_As_, −0.19; C_Sb_, −0.23), indicating the polar covalent nature of the C─E bonds. The WBIs (Wiberg bond indices) for the C─E (E = P, 1.11; As, 1.05; Sb, 0.93) and C─C (E = P, 1.34; As, 1.39; Sb, 1.45) bonds of the C_4_E_2_ ring of [**2**‐E]^●+^ suggest lowering of the C─E while raising of the C─C bond order with increasing the size of the pnictogen atoms (P < As < Sb). As expected for the heavier main group elements, the delocalization decreases (P > As > Sb) with increasing the size of the pnictogen atom (P < As < Sb). Consequently, the calculated spin density (Figure 5) on the antimony atoms in [**2**‐Sb]^●+^ amounts to 88% (i.e., 44% on each Sb), which is larger than that of 76% in [**2**‐P]^●+^ (i.e., 38% on each P) and 80% in [**2**‐As]^●+^ (i.e., 40% on each As). Interestingly, the C_Ph_ atom of the 1,3‐imidazole units of [**2**‐E]^●+^ also has a considerable spin density (8%–13%), while the same at the nitrogen atoms is negligible.^[^ ^82^, ^83^, ^84^, ^85^, ^86^ ^]^ This can be rationalized, considering the contribution of the C_Ph_ atoms to the αHOMO and βLUMO of the radical cations [**2**‐E]^●+^ (Figure 6). We also carried out fractional occupation number weighted density (FOD) calculations as an electron correlation diagnostic^[^ ^87^ ^]^ to examine the electronic structures of [**2**‐E]^●+^. FOD studies provide reliable information on the localization of “hot” (strongly correlated and chemically active) electrons in a molecule. The FOD plots of [**2**‐E]^●+^ (Figures S53–S55) nicely visualize the “hot” electrons, which are mostly located at the pnictogen atoms. The resulting FOD numbers (*N*_FOD_): 2.40 *e* for [**2**‐P]^●+^, 2.45 *e* for [**2**‐As]^●+^, 2.66 *e* for [**3**‐Sb]^●+^ suggest considerable electron correlations in [**2**‐E][B].^[^ ^65^, ^88^, ^89^, ^90^, ^91^ ^]^

The HOMO (highest occupied molecular orbital) and HOMO–1 of **1**‐E are essentially the p‐type lone pair orbitals on the pnictogen atoms of the C_4_E_2_ ring.^[^ ^63^, ^64^, ^65^ ^]^ One‐electron oxidation of **1**‐E (i.e., the removal of 1e from the HOMO of **1**‐E) to give the open‐shell species [**2**‐E]^●+^ is, in essence, the conversion of the HOMO of **1**‐E into the SOMO (singly occupied molecular orbital, i.e., αHOMO in the other notion) and the SUMO (singly unoccupied molecular orbital, i.e., βLUMO in the other notion) (Figure 6a) of [**2**‐E]^●+^. The SUMO (i.e., βLUMO) is the unoccupied (electron‐hole) counterpart to the SOMO (i.e., αHOMO). Further 1e‐oxidation of [**2**‐E]^●+^ results in the closed‐shell species [**3**‐E]^2+^, in which the HOMO of **1**‐E becomes the LUMO of [**3**‐E]^2+^. The SOMO and SUMO of [**2**‐P]^●+^ (Figure 6b), [**2**‐As]^●+^ (Figure S60), and [**2**‐Sb]^●+^ (Figure S61) are essentially the p‐type orbital on the pnictogen atoms. The αHOMO–1 and βHOMO are also mainly based on the pnictogen atoms of the C_4_E_2_ ring and have transannular antibonding character.

**Figure 7:**
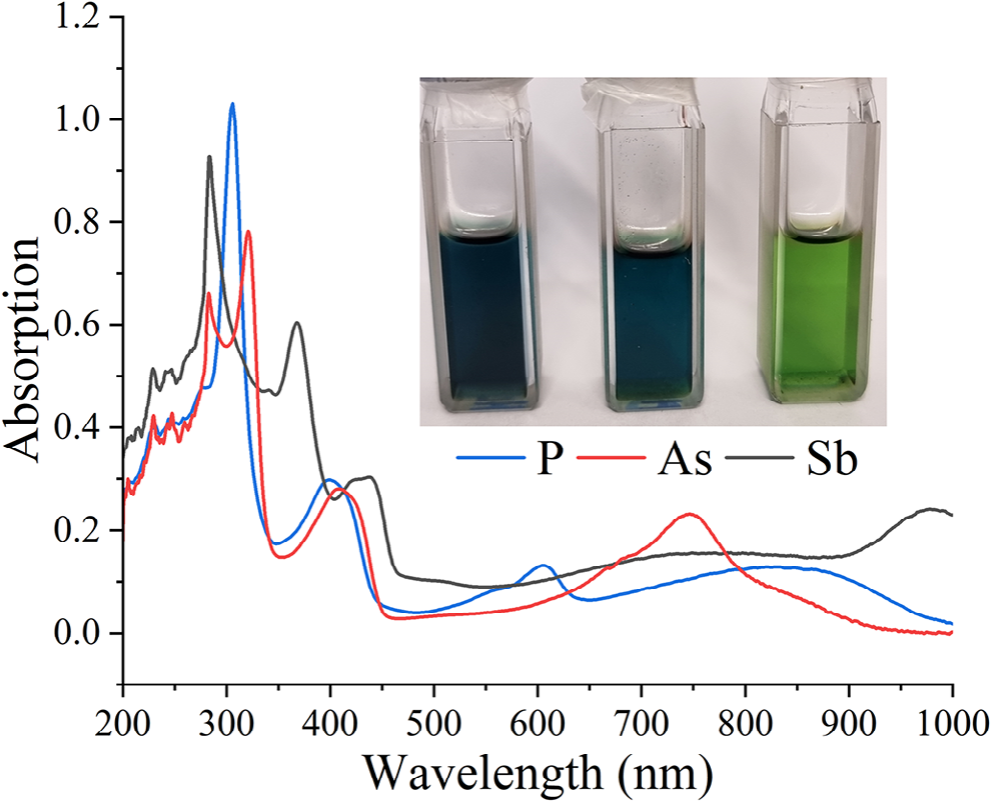
UV–Vis spectra of [**2**‐E][B1] measured in toluene.

The UV–Vis spectra of [**2**‐P][B1] (*λ*_max_ = 306, 399, 605, 820 nm), [**2**‐As][B1] (*λ*_max_ = 322, 409, 748 nm), and [**2**‐Sb][B1] (*λ*_max_ = 368, 438, 759, 978 nm) exhibit several absorption bands (Figure 7). Based on TD‐DFT calculations (Tables S6–S8), the lowest energy absorption band at 820 nm (for **2**‐P), 748 nm (for **2**‐As), and 978 nm (for **2**‐Sb) may be assigned to the αHOMO → αLUMO (722 nm), αHOMO → αLUMO (694 nm), and βHOMO → βLUMO (907 nm) transitions, respectively.

**Scheme 1:**
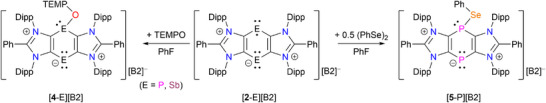
Reactivity of [**2**‐E][B2] with TEMPO and diphenyl diselenide.

Compounds [**2**‐E][B2] readily undergo radical coupling reactions with TEMPO (2,2,6,6‐ tetramethylpiperidinyloxyl) to afford [**4**‐E][B2] (E = P or Sb) as orange crystalline solids (Scheme 1). [**4**‐E][B2] are diamagnetic and exhibit well‐resolved ^1^H, ^11^B, ^13^C, ^19^F NMR signals expected for the ADC, [B2], and TEMPO moieties. Surprisingly, no reaction between [**2**‐As][B2] and TEMPO was observed under similar experimental conditions. The ^1^H NMR spectrum of [**4**‐P][B2] shows two septets and four doublets for the isopropyl groups, which is in line with its lower symmetry with respect to **1**‐P and [**3**‐P][B2]_2_. The ^31^P NMR spectrum of [**4**‐P][B2] shows two doublets at −4.4 and −79.8 ppm with the ^3^ *J*_PP_ coupling constant of 7.3 Hz. The ^31^P NMR chemical shifts^[^ ^92^ ^]^ and the ^3^ *J*_PP_ coupling constant^[^ ^93^, ^94^ ^]^ for [**4**‐P][B2] are consistent with those of related phosphorus heterocycles.

Treatment of [**2**‐P][B2] with diphenyl diselenide yields [**5**‐P][B2] as a red crystalline solid. Again, no reaction between [**2**‐As][B2] and diphenyl diselenide was observed, while treatment of [**2**‐Sb][B2] with diphenyl diselenide led to an intractable mixture of products. The ^1^H, ^13^C, ^11^B, ^19^F, ^31^P, and ^77^Se NMR spectra of [**5**‐P][B2] reveal expected signals. The ^31^P NMR spectrum of [**5**‐P][B2] exhibits two doublets at −25.5 and −44.8 ppm with ^3^ *J*_PP_ coupling constant of 15.2 Hz.^[^ ^93^, ^94^ ^]^ The ^31^P NMR signal at −25.5 ppm for [**5**‐P][B2] is accompanied by ^77^Se satellites (^1^ *J*_PSe_ = 243 Hz) and matches with that of Ph_2_PSePh (229 Hz),^[^ ^95^ ^]^ supporting the presence of a P─Se bond. Efforts to directly detect a ^77^Se NMR signal for [**5**‐P][B2] were unsuccessful.

## Conclusions

In conclusion, the first pnictogen radical cations [**2**‐E][B], featuring a central C₄E₂ ring with 7π electrons, have been successfully isolated as crystalline solids. These pnictogen‐centered radical species exhibit significant spin density (76%–88%) at the pnictogen atoms. Thus, they may be regarded as stable pnictogen analogs of the elusive aryl radical‐anion intermediates commonly involved in Birch reductions of arenes with alkali metals in liquid ammonia. Compounds [**2**‐E][B] have been thoroughly characterized using spectroscopic techniques (UV–Vis/EPR) and single‐crystal X‐ray diffraction (sc‐XRD), with their electronic structures further corroborated by quantum chemical calculations. The radical coupling reactivity of [**2**‐E][B2] has been demonstrated with TEMPO and diphenyl diselenide, yielding the diamagnetic compounds [**4**‐E][B2] (E = P or Sb) and [**5**‐P][B2], respectively. The successful synthesis of [**2**‐E][B], along with their dicationic counterparts [**3**‐E][B]₂, via stepwise single‐electron transfer (SET) underscores their potential in synthesis and beyond. The ability of [**2**‐E][B] and [**3**‐E][B]₂ to engage in SET reactions has promises in main‐group redox catalysis. This work lays the foundation for future explorations into the design and reactivity of pnictogen‐based radical species, potentially opening new avenues in synthetic and catalytic methodologies.

## Supporting Information

Experimental details, the plots of NMR, CV, EPR, and UV–Vis spectra as well as the detail of X‐ray crystallography and quantum chemical calculations of the reported compounds are given in the Supporting Information.

## Conflict of Interests

The authors declare no conflict of interest.

## Supporting information



Supporting Information S1

Supporting Information S2

Supporting Information S3

Supporting Information S4

## Data Availability

The data that support the findings of this study are available in the supplementary material of this article.
