# Supplementary material for: Annulated Radical Cations with a C4E2‐Core (E = P, As, Sb): Stable Pnictogen Analogs of Elusive Aryl Radical Anions of Birch Reduction Reactions
Source: Angew Chem Int Ed Engl. 2025 May 19;64(25):e202505142. doi: 10.1002/anie.202505142 (PMC12171318; doi:10.1002/anie.202505142)
Supplement: Supplementary file 1 — Supporting Information S1 [file ANIE-64-e202505142-s002.pdf]

Supporting Information

for

Annulated Radical Cations with a C_4E_2-Core (E = P, As, Sb): Stable Pnictogen Analogs of Elusive Aryl Radical Anions of Birch Reduction Reactions

Henric Steffenfauseweh,[a] Yury V. Vishnevskiy,[a] Beate Neumann,[a] Hans-Georg Stammler,[a] Demi D. Snabilié,[b] Bas de Bruin,[b] * and Rajendra S. Ghadwal,[a] *

[a]*Molecular Inorganic Chemistry and Catalysis, Inorganic and Structural Chemistry, Center for Molecular Materials, Faculty of Chemistry, Universität Bielefeld, Universitätsstrasse 25, D-33615, Bielefeld, Germany*

[b]*University of Amsterdam (UvA), Faculty of Science, Van 't Hoff Institute for Molecular Sciences (HIMS), Homogeneous and Supramolecular Catalysis Group, Science Park 904, 1098 XH Amsterdam, The Netherlands*

**E-Mail: rghadwal@uni-bielefeld.de (RSG); b.debruin@uva.nl* (BdB)
http://www.ghadwalgroup.de; Fax: +49 521 106 6026; Tel: +49 521 106 6167

TABLE OF CONTENTS

Materials and Methods S1
Synthesis of [(ADC)E]$_2$[B] [**2**-E][B] S1
Alternative Synthesis of [**2**-E][B1] from **1**-E and [**3**-E][B1]$_2$ S3
Synthesis of Dicationic Compounds [(ADC)E]$_2$[B1]$_2$ [**3**-E][B1]$_2$ (E = P or As) S3
Reaction of [**2**-E][B2] with TEMPO S4
Reaction of [(ADC)E]$_2$[B{$C_6H_3(CF_3)_2$}$_4$] with Se_2Ph_2 .... S6
NMR Plots S7
UV-Vis. Spectra S22
Cyclic Voltammetry (CV) S26
EPR Spectroscopy S28
Crystallographic Details S31
Computational Details S36
References S55

Materials and Methods

All syntheses and manipulations were carried out under an inert gas (Ar or N_2) atmosphere using standard *Schlenk* techniques or a glove-box (MBraun LABMasterPro). Organic solvents were dried over appropriate drying agents, distilled, and stored over 3 Å molecular sieve. Nano-ESI mass spectra were recorded using an Esquire 3000 ion trap mass spectrometer (Bruker Daltonik GmbH, Bremen, Germany) equipped with a nano-ESI source. Samples were dissolved in toluene and introduced by static nano-ESI using in-house pulled glass emitters. Nitrogen served both as nebulizer as well as dry gas and was generated by a Bruker nitrogen generator NGM 11. Helium served as cooling gas for the ion trap. The mass axis was externally calibrated with ESI-L Tuning Mix (Agilent Technologies, Santa Clara, CA, USA) as a calibration standard. Melting points (MPs) were measured using a Büchi B-545 melting point apparatus. Deuterated solvents were dried over appropriate drying agents, distilled, and stored inside a glove box. NMR spectra were recorded on a Bruker Avance III 500 or a Bruker Avance III 500 HD spectrometer. Chemical shifts (in *δ*, ppm) are referenced to the residual solvent signal(s): CD_2Cl_2 (^{1}H, 5.32; ^{13}C, 53.84), CD_3CN (^{1}H, 1.94; ^{13}C, 1.32), C_6D_6 (^{1}H, 7.16; ^{13}C, 128.06), and THF-d_8 (^{1}H, 3.58; ^{13}C, 67.21 ppm).[1]. UV-visible spectra were recorded on a Genesys 50 UV-visible spectroscopy system by Thermos Scientific. (IPr-Ph)Br,[2] Ni(cod)$_2$,[3] KC_8,[4] $Ph_3C[B(C_6F_5)_4]$,[5] $Ph_3C[B\{3,5\text{-}(CF_3)_2C_6H_3\}_4]$[6] and $[(ADC)E]_2$ (**1**-E)[7] were prepared according to literature protocols.

Synthesis of [(ADC)E]$_2$[B] [2-E][B]

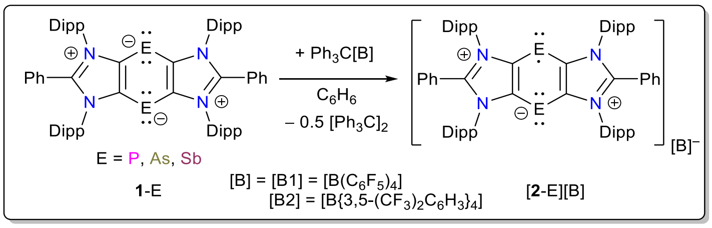

Images of solid samples of [**2**-E][B1]:

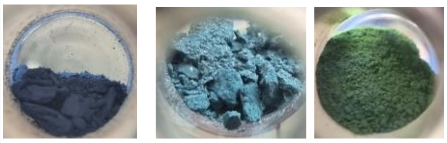

[**2**-P][B1] [**2**-As][B1] [**2**-Sb][B1]

*Synthesis of [(ADC)P]$_2$[B1] [**2**-P][B1]:* To a mixture of **1**-P (202 mg, 0.20 mmol) and Ph_3C[B1] ([B1] = $[B(C_6F_5)_4]$) (188 mg, 0.20 mmol) was added 10 mL of benzene at room temperature (rt). The resulting dark blue suspension was stirred for 5h at rt. The dark blue precipitate formed was isolated by filtration and then washed twice with 5 mL benzene. The volatiles were removed under vacuum to afford compound (**2**-P)[B1] as a dark blue solid in 85% yield (280 mg). Suitable single crystals for X-ray diffraction (sc-XRD) were obtained by storing a saturated toluene solution of [**2**-P][B1] at rt for 3 days. **MP**: 278 °C (dec.). Elemental analyses (%) calculated for $C_{90}H_{78}N_4BF_{20}P_2$ (1668.37) [**2**-P][B1]: C 64.79, H 4.71, N 3.36; found: C 64.19, H 4.90, N 3.31. **ESI (m/z)**: 1021.41 ([**2**-P] + $CH_3OH)^+$. **UV/Vis** (in toluene) λ (nm) (ε (M^{-1} cm^{-1})): 282 (8000); 306 (18667); 399 (5167); 558 (1500); 605 (2333), 820 (2250).

*Synthesis of [(ADC)As]$_2$[B1] [**2**-As][B1]:* Compound [**2**-As][B1] was prepared by employing a similar method as discussed above for [**2**-P][B1] using **1**-As (205 mg, 0.19 mmol) and Ph_3C[B1] (173 mg, 0.19 mmol) as a turquoise solid in 69% yield (225 mg). Crystals for sc-XRD analysis were obtained by storing a saturated toluene solution of [**2**-As][B1] at rt for 5 days. **MP**: 274 °C (dec.). Elemental analyses (%) calculated for $C_{90}H_{78}N_4BF_{20}As_2$ (1756.26) [**2**-As][B1]: C 61.55, H 4.48, N 3.19; found: C 60.36, H 4.86, N 3.21. **ESI (m/z)**: 1109.33 ([**2**-As] + $CH_3OH)^+$. **UV/Vis** (in toluene) λ (nm) (ε (M^{-1} cm^{-1})): 282 (10476), 322 (12380), 409 (4444), 748 (3651).

*Synthesis of [(ADC)Sb]$_2$[B1] [**2**-Sb][B1]:* Compound [**2**-Sb][B1] was prepared by employing a similar method as discussed above for [**2**-P][B1] using **1**-Sb (202 mg, 0.17 mmol) and Ph_3C[B1] (156 mg, 0.17 mmol) as a dark green solid in 48% yield (150 mg). **ESI (m/z)**: 1117.40 ([**2**-Sb] – Ph + $Na)^+$. **UV/Vis** (in toluene) λ (nm) (ε (M^{-1} cm^{-1})): 284 (12850); 370 (7050); 422 (3950); 438 (3950); 709 (1500) and 983 (2300).

*Synthesis of [(ADC)P]$_2$[B2] [**2**-P][B2]:* To a mixture of **1**-P (314 mg, 0.31 mmol) and Ph_3C[B2] ([B2] = B{3,5-$(CF_3)_2(C_6H_3\}_4$) (336 mg, 0.31 mmol) was added 10 mL fluorobenzene at rt. The resulting blue solution was stirred overnight at rt. The volatiles were removed under vacuum. The resulting residue was suspended in 20 mL *n*-hexane and sonicated to precipitate [**2**-P][B2] as a blue crystalline solid in 84% yield (502 mg). **MP**: 164 °C (dec.). Elemental analyses (%) calculated for $C_{98}H_{90}BF_{24}N_4P_2$ (1852.55) [**2**-P][B2]: C 63.54, H 4.90, N 3.02; found: C 63.09, H 4.73, N 2.85. **ESI (m/z)**: 1021.41 ([**2**-P] + $CH_3OH)^+$.

*Synthesis of [(ADC)As]$_2$[B2] [**2**-As][B2]:* Compound [**2**-As][B2] was prepared by employing a similar method as discussed above for [**2**-P][B2] using **1**-As (501 mg, 0.46 mmol) and Ph_3C[B2] (520 mg, 0.47 mmol) as a turquoise crystalline solid in 64% yield (590 mg).**MP**: 113 °C (dec.). **ESI (m/z)**: 1109.33 ([**2**-As] + $CH_3OH)^+$.

*Synthesis of [(ADC)Sb]$_2$[B2] [**2**-Sb][B2]:* Compound [**2**-Sb][B2] was prepared by employing a similar method as discussed above for [**2**-P][B2] using **1**-Sb (360 mg, 0.31 mmol) and Ph_3C[B2] (340 mg, 0.31 mmol) as a lemon-green crystalline solid in 87% yield (545 mg). Suitable crystals for sc-XRD analysis were obtained by storing a saturated toluene solution of [**2**-Sb][B2] at rt for 7 days. **MP**: 169 °C (dec.). Elemental analyses (%) calculated for $C_{98}H_{90}BF_{24}N_4Sb_2$ (2034.12) [**2**-Sb][B2]: C 57.87, H 4.46, N 2.75; found: C 56.78, H 4.58, N 2.59. **ESI (m/z)**: 1117.40 ([**2**-Sb] – Ph + H + $Na)^+$. **UV/Vis** (in toluene) λ (nm) (ε (M^{-1} cm^{-1})): 282 (30000), 368 (19063) 430 (9375), 759 (5000), 978 (7500).

Alternative Synthesis of [2-E][B1] from 1-E and [3-E][B1]$_2$

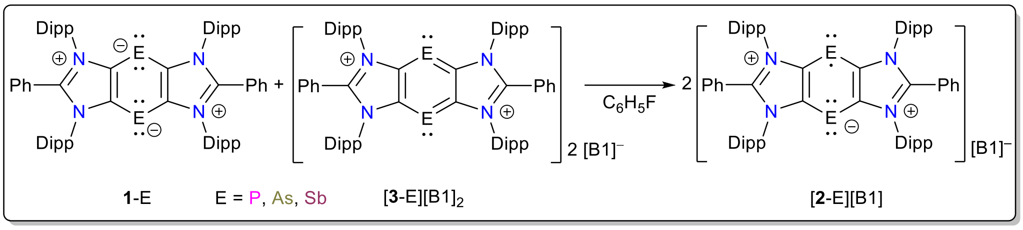

General Procedure: To a *Schlenk* tube containing a 1:1 mixture of **1**-E and [**3**-E][B1]$_2$ was added 5 mL fluorobenzene at rt, which led to an immediate color change (see above). The products [**2**-E][B1] were isolated in an almost quantitative yield after usual workup. They showed similar analytic properties (UV-vis and EPR spectra) as presented above.

Synthesis of Dicationic Compounds [(ADC)E]$_2$[B1]$_2$ [3-E][B1]$_2$ (E = P or As)

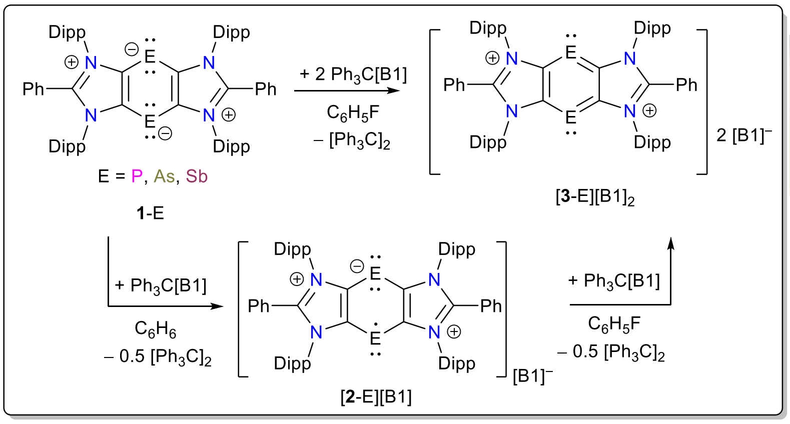

images of solid samples of [3-E][B1]:

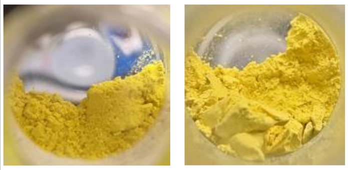

[3-P][B1]$_2$ [3-As][B1]$_2$

Synthesis of [(ADC)P]$_2$[B1]$_2$ [3-P][B1]$_2$: To a mixture of **1**-P (103 mg, 0.10 mmol) and Ph_3C[B1] (194 mg, 0.21 mmol) was added 15 mL fluorobenzene at rt. After stirring overnight at rt, the precipitate formed was isolated by filtration, washed with fluorobenzene (3 x 2 mL) and benzene (1x 5 mL), and dried under vacuum to yield [**3**-P][B1]$_2$ as a yellow solid in 72% yield (170 mg). **MP**: 356 °C (dec.). Elemental analyses (%) calculated for $C_{132}H_{93}B_2F_{43}N_4P_2$ (2635.72), [**3**-P][B1]$_2$ + 3 C_6H_5F: C 60.15, H 3.56, N 2.13; found: C 59.62, H 3.66, N 2.07. **ESI (m/z)**: 494.21 ([**3**-P])$^{2+}$. **^{1}H NMR** (500 MHz, THF-d_8, 298 K): 7.82 (t, J = 7.9 Hz, 4H, p-C_6H_3), 7.74 (t, J = 7.6 Hz, 2H, p-C_6H_5), 7.66 (d, J = 7.9 Hz, 8H, m-C_6H_3), 7.53 (t, J = 7.6 Hz, 4H, m-C_6H_5), 7.35 (m, 4H, o-C_6H_5), 2.32 (sept, J = 6.8 Hz, 8H, $CH(CH_3)_2$), 1.11 (d, J = 6.8 Hz, 24H, $CH(CH_3)_2$), 1.00 (d, J = 6.8 Hz, 24H, $CH(CH_3)_2$) ppm. **^{13}C{^{1}H} NMR** (125 MHz, C_6D_6, 298 K): 165.1 (CP), 163.1 (NCN), ~~156.4,~~ 150.3 (C_6F_5), 148.4 (C_6F_5), 146.4 ($ipso$-C_6H_3), 138.3 (C_6F_5), 137.5 (p-C_6H_5), 136.3 (C_6F_5), 135.7 (p-C_6H_3), 131.5 (o-C_6H_5), 131.4 (m-C_6H_5), 130.4, 129.1 (o-C_6H_3), 128.6 (m-C_6H_3), 119.6 ($ipso$-C_6H_3), 30.9 ($CHMe_2$), 26.0 ($CH(CH_3)_2$), 23.5 ($CH(CH_3)_2$) ppm. **^{11}B{^{1}H} NMR** (160 MHz, C_6D_6, 298 K): −16.6 ppm. **^{19}F{^{1}H} NMR** (471 MHz, C_6D_6, 298 K): −132.6 (s, o-C_6F_5), −164.9 (t, J = 20.0 Hz, p-C_6F_5), −168.4 (t, J = 20.0 Hz, m-C_6F_5)

ppm. **^{31}P NMR** (202 MHz, C_6D_6, 298 K): 117.8 (s) ppm. **UV/Vis** (in THF) λ (nm) (ε (M^{-1} cm^{-1})): 236 (24651); 260 (30000); 294 (35249); 355 (12791); 417 (5814); 456 (4419).

*Synthesis of [(ADC)As]$_2$[B1]$_2$ [**3**-As][B1]$_2$:* **1**-As (103 mg, 0.09 mmol) and $Ph_3C[B(C_6F_5)_4]$ (172 mg, 0.19 mmol) were dissolved in fluorobenzene (5 mL) and stirred at room temperature overnight. The resulting green suspension was filtered, washed with fluorobenzene (3x 2 mL) and benzene (1x 5 mL) to yield [**3**-As][B1]$_2$ as a yellow solid in 75% yield (172 mg). **MP**: 364 °C (dec.). Elemental analyses (%) calculated for $C_{114}H_{78}B_2F_{40}N_4As_2$ (2435.30) (**3**-As): C 56.23, H 3.23, N 2.30; found: C 56.10, H 3.68, N 1.89. **ESI (m/z)**: 538.21 ([**3**-As])$^{2+}$. **^{1}H NMR** (500 MHz, THF-*d*8, 298 K): 7.83 (m, 4H, *p*-C_6H_3), 7.66 (br m, 10H, *p*-C_6H_5 and *m*-C_6H_3), 7.51 (d, *J* = 7.8 Hz, 2H, *m*-C_6H_5), 7.47 (d, *J* = 7.8 Hz, 2H, *m*-C_6H_5), 7.34 (underlying 2H, *o*-C_6H_5), 7.23 (t, *J* = 7.5 Hz, 2H, *o*-C_6H_5), 2.37 (br s, 8H, C*H*Me$_2$), 1.12 (br s, 24H, CH(CH_3)$_2$), 1.02 (br s, 24H, CH(CH_3)$_2$) ppm. **^{13}C{^{1}H} NMR** (126 MHz, C_6D_6, 298 K): 165.1 (*C*As), 163.1 (NCN), 150.3 (*C*$_6F_5$), 148.4 (*C*$_6F_5$), 138.3 (*C*$_6F_5$), 136.3 (*C*$_6F_5$), 135.5, 135.2, 130.4, 128.8 (*m*-*C*$_6H_3$), 30.2 (*C*HMe$_2$), 23.7 (CH(*C*H$_3$)$_2$), 23.5 (CH(*C*H$_3$)$_2$) ppm. **^{11}B{^{1}H} NMR** (160 MHz, C_6D_6, 298 K): −16.6 (br. s) ppm. **^{19}F{^{1}H} NMR** (471 MHz, C_6D_6, 298 K): −132.7 (s, *o*-C_6F_5), −165.0 (t, *J* = 20.0 Hz, *p*-C_6F_5), −168.5 (t, *J* = 20.0 Hz, *m*-C_6F_5) ppm. **UV/Vis** (in THF) λ (nm) (ε (M^{-1} cm^{-1})): 236 (15254); 262 (18814); 295 (19492); 426 (8814); 449 (7966); 748 (1186).

Alternative synthesis of [(ADC)P]$_2$[B1]$_2$: To a mixture of [**2**-P][B1] (20 mg, 0.01 mmol) and Ph_3C[B1] (11 mg, 0.01 mmol) was added 5 mL fluorobenzene 5 mL and stirred at rt overnight. The resulting green precipitate was isolated by filtration, washed with fluorobenzene (3 x 1 mL) and benzene (1 x 3 mL), and dried under vacuum to give [**3**-P][B1]$_2$ as a yellow solid in 97% yield (27 mg).

Alternative synthesis of [(ADC)As]$_2$[B1]$_2$.]: Compound [**2**-As][B1]$_2$ was prepared by employing a similar method as discussed above for [**3**-P][B1]$_2$ using [**2**-As][B1] (21 mg, 0.01 mmol) and $Ph_3C[B(C_6F_5)_4]$ (12 mg, 0.01 mmol) as a yellow solid in 86% yield (25 mg).

Reaction of [2-E][B2] with TEMPO

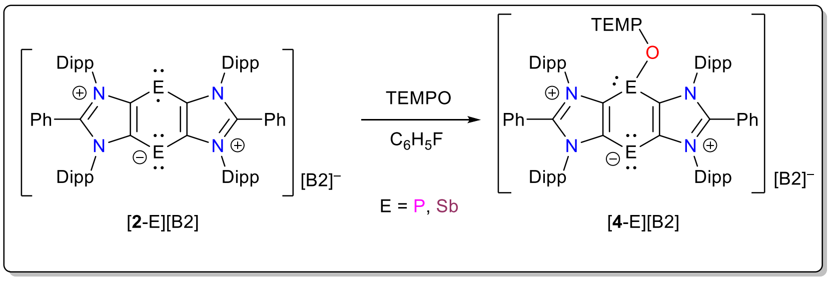

images of solid samples of [**4**-E][B2]:

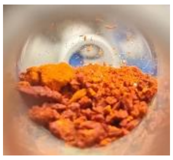

[**4**-P][B2]

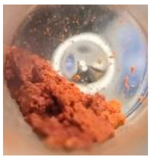

[**4**-Sb][B2]

*Synthesis of [{(ADC)P}$_2$(TEMPO)][B2] [**4**-P][B2]:* To a Schlenk flask containing [**2**-P][B2] (104.0 mg, 0.05 mmol) and TEMPO (10.1 mg, 0.06 mmol) was added 8 mL of fluorobenzene. An immediate color change from blue to red was observed and the solution was stirred overnight. The volatiles were

removed under vacuum to afford a red solid. Washing twice with 5 mL of *n*-hexane and drying under vacuum affords [**4**-P][B2] in 93% yield (101.0 mg). **MP**: 240 °C (dec.). ^{1}**H NMR** (500 MHz, C_6D_6, 298 K): 8.46 – 8.41 (m, 14H, *o*-$C_6H_3(CF_3)_2$), 7.71 (s, 7H, *p*-$C_6H_3(CF_3)_2$), 6.99 (d, J = 7.5 Hz, 8H, *m*-C_6H_3), 6.92 – 6.87 (m, 8H, *o*-C_6H_5, *m*-C_6H_5), 6.77 (t, J = 7.0, 8.0 Hz, 2H, *p*-C_6H_3), 6.53 (t, J = 8.0 Hz, 4H, *p*-C_6H_5), 3.00 (sept, J = 6.8 Hz, 4H, C*H*$(CH_3)_2$), 2.81 (sept, J = 6.8 Hz, 4H, C*H*$(CH_3)_2$), 1.31 (d, J = 6.6 Hz, 12H, CH(C*H*$_3$)$_2$), 1.26 (d, J = 6.8 Hz, 12H, CH(C*H*$_3$)$_2$), 1.24 (m, 12H, C(C*H*$_3$)$_2$), 0.89 (t, J = 6.8 Hz, 12H, CH(C*H*$_3$)$_2$), 0.80 (d, J = 6.8 Hz, 12H, CH(C*H*$_3$)$_2$), 0.76 (d, J = 6.8 Hz, 12H) ppm. ^{13}**C{^{1}H} NMR** (125 MHz, C_6D_6, 298 K): 163.0 (B2), 147.6 (*o*-*C*$_6$H$_3$), 145.9 (*ipso*-*C*$_6$H$_3$), 135.6 (B2), 133.3 (*C*$_6$H$_3$), 132.0 (*C*$_6$H$_3$), 131.7 (*C*$_6$H$_5$), 131.2 (*C*$_6$H$_5$), 130.7 (B2), 130.1 (*C*$_6$H$_3$), 129.8 (*C*$_6$H$_5$), 128.7 (*o*-C$_6$*H*$_5$), 128.5 (*p*-*C*$_6$H$_5$), 126.4 (B2), 125.8, 124.7 (*m*-C$_6$*H*$_3$), 124.3 (B2), 122.5 (*C*$_6$H$_3$), 122.1 (*C*$_6$H$_5$), 118.2 (B2), 32.0 (TEMPO), 29.6, 29.2 (*C*H(CH$_3$)$_2$), 25.0, 23.8 (TEMPO), 23.3, 23.1 (CH(*C*H$_3$)$_2$), 14.4 (TEMPO) ppm. ^{11}**B{^{1}H} NMR** (160 MHz, C_6D_6, 298 K): 5.9 (p, $^{5}J_{BF}$ = 2.79 Hz) ppm. ^{19}**F{^{1}H} NMR** (471 MHz, C_6D_6, 298 K): −62.0 ppm. ^{31}**P NMR** (202 MHz, C_6D_6, 298 K): −4.4 (d, $^{4}J_{PP}$ = 7.3 Hz), −79.8 (d, $^{4}J_{PP}$ = 7.3 Hz) ppm. Elemental analyses (%) calculated for $C_{107}H_{108}BF_{24}N_5OP_2$ (2008.79) ([**4**-P][B2]): C 63.98, H 5.42, N 3.49; found: C 63.80, H 5.02, N 3.31. **UV-Vis** (in toluene) λ (nm) (ε (M^{-1} cm^{-1})): 295 (22471), 360 (7059) and 509 (4471).

*Synthesis of [{(ADC)Sb}$_2$(TEMPO)][B2] [**4**-Sb][B2]:* A mixture of [**2**-Sb][B2] (21 mg, 0.01 mmol) and TEMPO (1.5 mg, 0.01 mmol) was dissolved in C_6D_6 in a Young-NMR tube. An immediate color change from green to red was observed. ^{1}**H NMR** (500 MHz, C_6D_6, 298 K): 8.44 (s, 8H, *o*-$C_6H_3(CF_3)_2$), 7.69 (s, 4H, *p*-$C_6H_3(CF_3)_2$, 4H), 7.21 (t, J = 7.8 Hz, 2H, *p*-C$_6$*H*$_3$), 7.07 (m, 6H, *m*-C$_6$*H*$_3$, *p*-C$_6$*H*$_3$), 6.97, (m, 6H, *p*-C$_6$*H*$_3$, m-C$_6$*H*$_5$), 6.77 (d, J = 7.5 Hz, 2H, *o*-C$_6$*H*$_5$), 6.65 (t, J = 7.5 Hz, 2H, *p*-C$_6$*H*$_5$), 6.58 (t, J = 7.8 Hz, 4H, *m*-C$_6$*H*$_3$), 3.23 (sept, J = 6.8 Hz, 2H, C*H*$(CH_3)_2$), 2.68 (sept, J = 6.8 Hz, 2H, C*H*$(CH_3)_2$), 2.58 (sept, J = 6.8 Hz, 2H, C*H*$(CH_3)_2$), 2.15 (sept, J = 6.8 Hz, 2H, C*H*$(CH_3)_2$), 1.58 (d, J = 6.8 Hz, 6H, CH(C*H*$_3$)$_2$), 1.32 (d, J = 6.8 Hz, 6H, CH(C*H*$_3$)$_2$), 1.21 (br m, 18H, br m, 20H, CH(C*H*$_3$)$_2$, C(C*H*$_3$)$_2$), 1.12 (m, 14H, CH(C*H*$_3$)$_2$, C*H*$_2$), 0.91 (br m, 20H, CH(C*H*$_3$)$_2$, C(C*H*$_3$)$_2$, C*H*$_2$) 0.72 (d, J = 7.8, 2H, C*H*$_2$) ppm. ^{13}**C{^{1}H} NMR** (125 MHz, C_6D_6, 298 K): 163.4 (N*C*N), 163.0 (N*C*N), 162.6 (B2), 162.3 (N*C*N), 161.6 (*C*Sb), 161.6 (*C*Sb), 146.3, 145.0, 144.9, 144.6, 144.5 (*C*$_6$H$_3$), 135.5 (B2), 133.3 (*C*$_6$H$_5$), 133.2 (*p*-*C*$_6$H$_3$), 132.1 (*C*$_6$H$_5$), 132.0 (*p*-C$_6$*H*$_5$), 131.7 (*m*-C$_6$*H*$_3$, *p*-*C*$_6$H$_3$), 130.1 (B2), 129.8 (*C*$_6$H$_5$), 128.9 (*m*- *C*$_6$H$_5$), 128.8 (*m*-C$_6$*H*$_3$), 126.7 (*o*-*C*$_6$H$_5$), 126.4 (B2), 125.5 (*C*$_6$H$_5$), 124.2 (B2), 122.7 (*C*$_6$H$_5$), 118.1 (B2), 60.4, 39.9, 32.0 (TEMPO), 29.5, 29.3, 29.1, 28.9 (*C*H(CH$_3$)$_2$), 26.7, 25.7, 25.6, 24.8, 24.5, 24.1, 23.2 (CH(*C*H$_3$)$_2$), 23.0, 22.5, 22.0, 17.2, 14.4 (TEMPO) ppm. ^{11}**B{^{1}H} NMR** (160 MHz, C_6D_6, 298 K): −5.9 ppm. ^{19}**F{^{1}H} NMR** (471 MHz, C_6D_6, 298 K): −62.0 ppm.

Reaction of $[(ADC)E]_2[B\{C_6H_3(CF_3)_2\}_4]$ with Se_2Ph_2

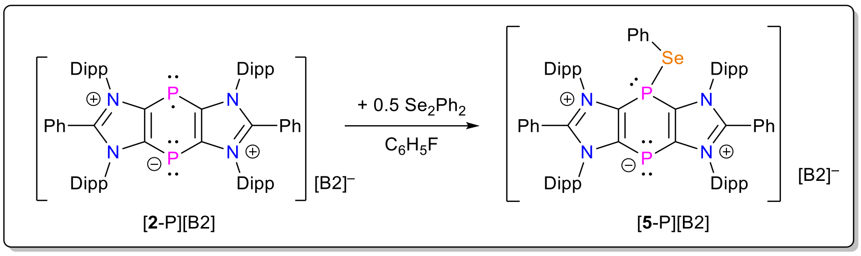

image of solid sample

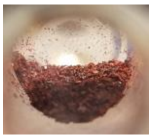

[5-P][B2]

Synthesis of $[\{(ADC)P\}_2(SePh)][B2]$ [5-P][B2]: To a Schlenk flask containing [**2**-P][B2] (98.0 mg, 0.05 mmol) and Se_2Ph_2 (9.4 mg, 0.03 mmol) was added 8 mL of fluorobenzene. An immediate color change from blue to red was observed and the solution was stirred overnight. All volatiles were removed under vacuum to afford a red solid. Washing twice with 5 mL of *n*-hexane and drying under vacuum affords [**5**-P][B2] in 93 % yield (103 mg). **MP**: 113 °C (dec.). **^{1}H NMR** (600 MHz, C_6D_6, 298 K): δ 8.44 (s, 8H, *o*-$C_6H_3(CF_3)_2$), 7.70 (s, 4H, *p*-$C_6H_3(CF_3)_2$), 7.50 (m, 1H, *p*-C_6H_5–Se), 7.31 (m, 2H, *m*-C_6H_5–Se), 7.21 (m, 2H, 1H, *o*-C_6H_5–Se), 7.05 (m, 4H, *m*-C_6H_3), 6.94 (d, J = 7.7 Hz, 4H, *o*-C_6H_5), 6.74 (m, 2H, *p*-C_6H_3), 6.70 (m, 4H, *m*-C_6H_3), 6.60 (t, J = 7.5 Hz, 2H, *p*-C_6H_3), 6.53 (t, J = 7.7 Hz, 4H, *m*-C_6H_5), 6.40 (d, J = 7.7 Hz, 2H, *p*-C_6H_5), 3.18 (sept, J = 6.8 Hz, 2H, C*H*$(CH_3)_2$), 2.83 (sept, J = 6.8 Hz, 2H, C*H*$(CH_3)_2$), 2.59 (sept, J = 6.8 Hz, 2H, C*H*$(CH_3)_2$), 2.08 (sept, J = 6.8 Hz, 2H, C*H*$(CH_3)_2$), 1.64 (d, J = 6.8 Hz, 6H, $CH(CH_3)_2$), 1.25 (d, J = 6.8 Hz, 18H, $CH(CH_3)_2$), 0.96 (d, J = 6.8 Hz, 6H, $CH(CH_3)_2$), 0.72 (d, J = 6.8 Hz, 6H, $CH(CH_3)_2$), 0.33 (d, J = 6.8 Hz, 6H, $CH(CH_3)_2$), 0.17 (d, J = 6.8 Hz, 6H, $CH(CH_3)_2$) ppm. **^{13}C{^{1}H} NMR** (150 MHz, C_6D_6, 298 K): 163.4 (N*C*N), 163.0 (B2), 162.7 (N*C*N), 162.4 (N*C*N), 160.8 (br. d, $^1J_{CP}$ = 50.0 Hz, *C*P), 147.8 (*ipso*-SeC_6H_5), 147.3 (C_6H_5), 146.5, 145.8, 145.5, 144.8 (*ipso*-C_6H_3), 135.5 (B2), 132.5 (C_6H_5), 132.3 (*p*-C_6H_3), 131.7 (*p*-SeC_6H_5), 131.7 (*m*-SeC_6H_5), 131.1 (C_6H_3), 130.9 (*o*-C_6H_3), 130.6 (*p*-C_6H_5), 130.4 (C_6H_5), 130.3 (C_6H_3), 130.1 (B2), 129.9, 129.6 (C_6H_5), 129.4 (*m*-C_6H_3), 129.3 (*m*-C_6H_3), 128.9 (*o*-C_6H_5), 128.8 (*m*-C_6H_5), 126.5 (C_6H_5), 126.3 (B2), 126.2 (*p*-C_6H_3), 126.1 (*o*-C_6H_3), 125.9 (*o*-SeC_6H_5), 124.4 (B2), 122.6 (B2), 122.1 (C_6H_3), 118.1 (B2), 29.8, 29.7, 29.7, 29.3 ($CH(CH_3)_2$), 24.9, 24.7, 24.1, 23.9, 23.7, 23.6, 23.1, 22.1 ($CH(CH_3)_2$), ppm. **^{11}B{^{1}H} NMR** (160 MHz, C_6D_6, 298 K): −5.9 (s) ppm. **^{19}F{^{1}H} NMR** (471 MHz, C_6D_6, 298 K): −62.0 ppm. **^{31}P NMR** (202 MHz, C_6D_6, 298 K): −25.5 (d, $^4J_{P–P}$ = 15.2 Hz, $^1J_{P–Se}$ = 263 Hz, PSe), −44.8 (d, J = 15.2 Hz) ppm. **^{1}H-^{77}Se{^{1}H} HMBC NMR** (500/95 MHz, C_6D_6, 298 K): 333.7 ppm. Elemental analyses (%) calculated for $C_{104}H_{95}BF_{24}N_4P_2Se$ (2008.62) [**5**-P][B2]: C 62.19, H 4.77, N 2.79; found: C 62.51, H 4.92, N 2.66. **UV/Vis** (in toluene) λ (nm) (ε (M^{-1} cm^{-1})): 298 (36400), 354 (16800), 430 (6500).

NMR Plots

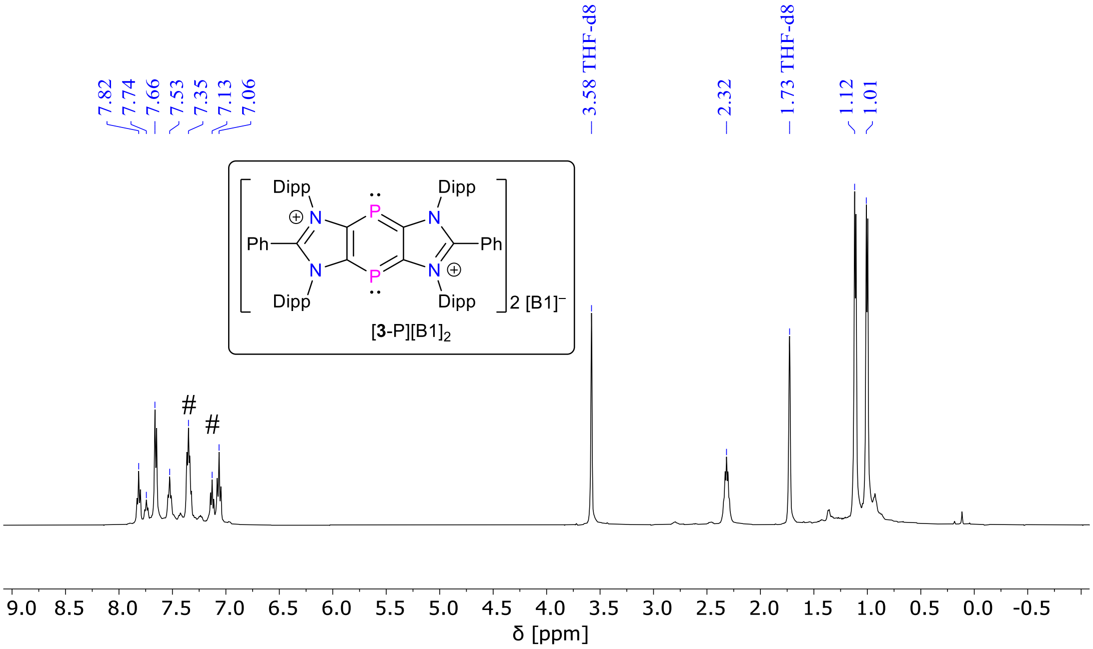

Figure S1. ^{1}H NMR (500 MHz, THF-*d*8, 298 K) of [**3**-P][B1]$_2$. $^{\#}C_6H_5F$.

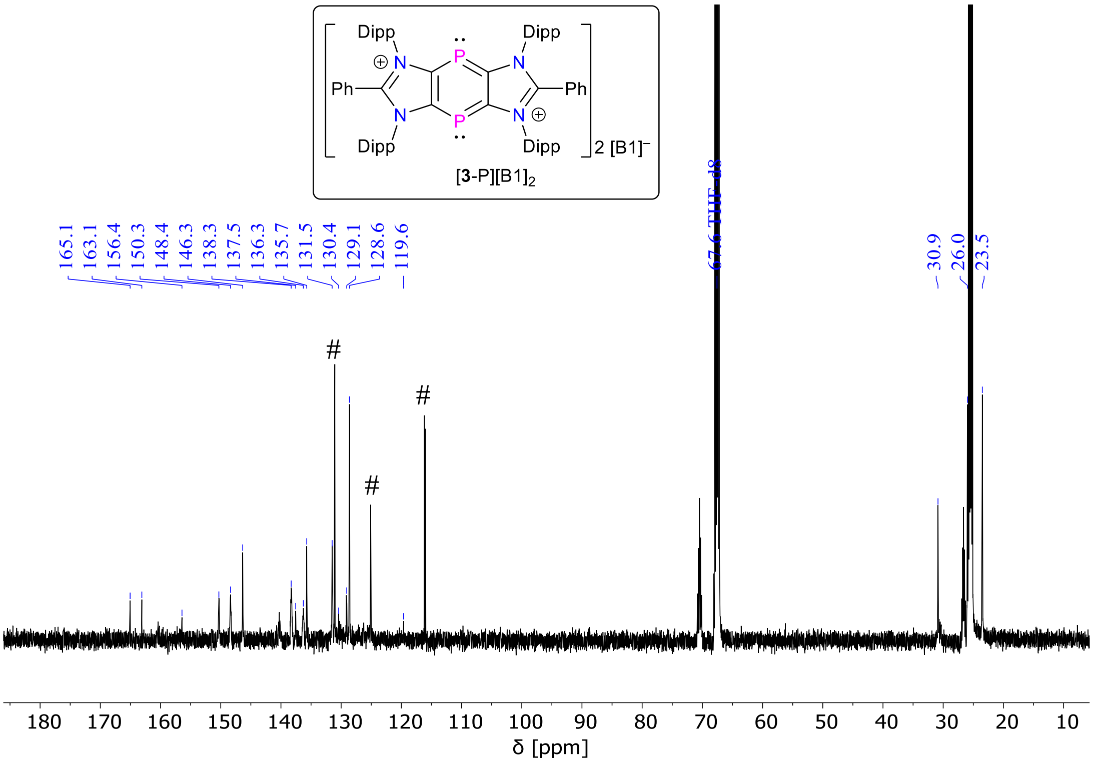

Figure S2. ^{13}C{^{1}H} NMR (125 MHz, THF-*d*8, 298 K) of [**3**-P][B1]$_2$. $^{\#}C_6H_5F$.

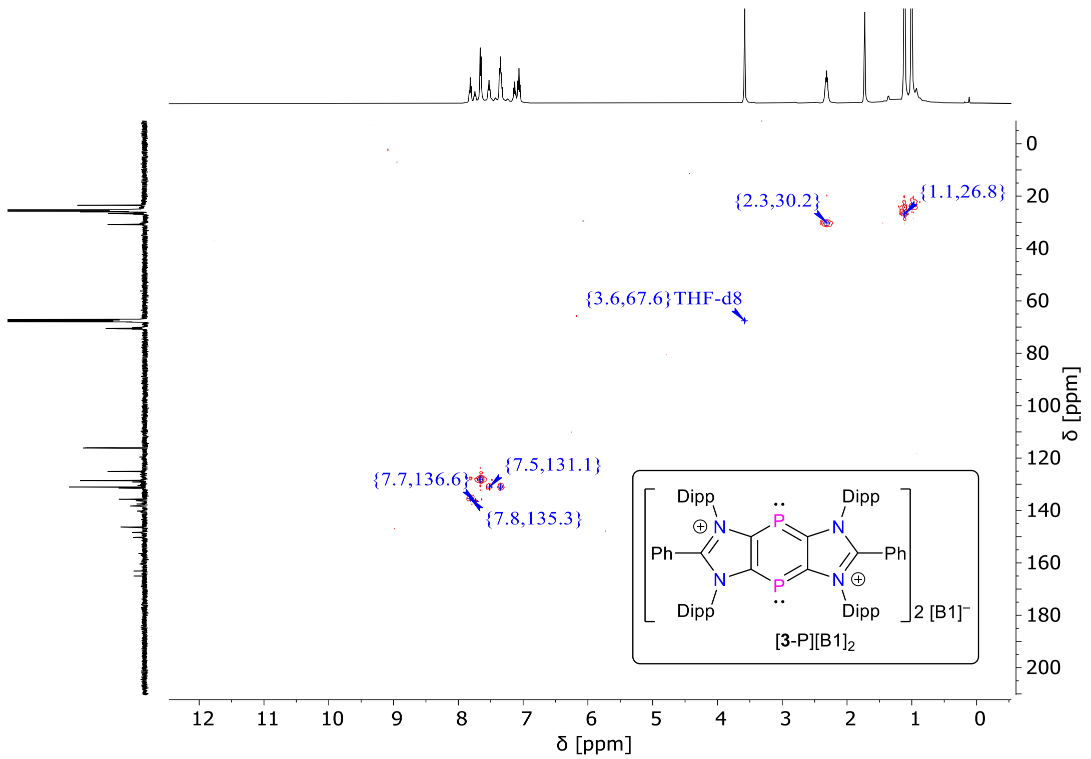

Figure S3. ^{1}H-^{13}C{^{1}H} HMQC NMR (500/125 MHz, THF-*d*8, 298 K) of [**3**-P][B1]$_2$.

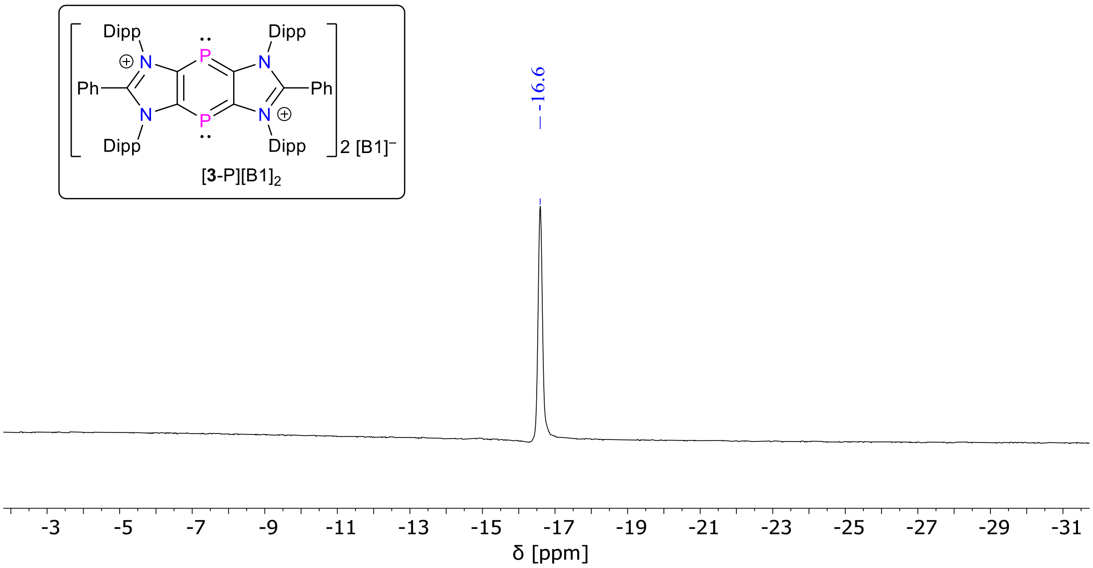

Figure S4. ^{11}B{^{1}H} NMR (160 MHz, THF-*d*8, 298 K) of [**3**-P][B1]$_2$.

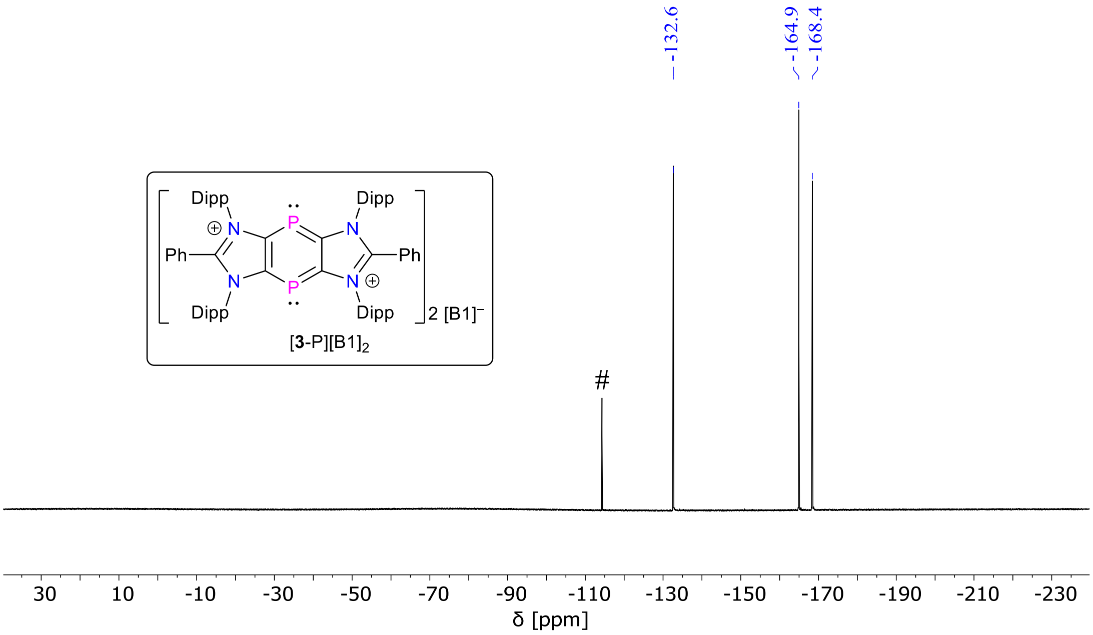

Figure S5. $^{19}F\{^{1}H\}$ NMR (471 MHz, THF-*d*8, 298 K) of [**3**-P][B1]$_2$.$^{\#}C_6H_5F$.

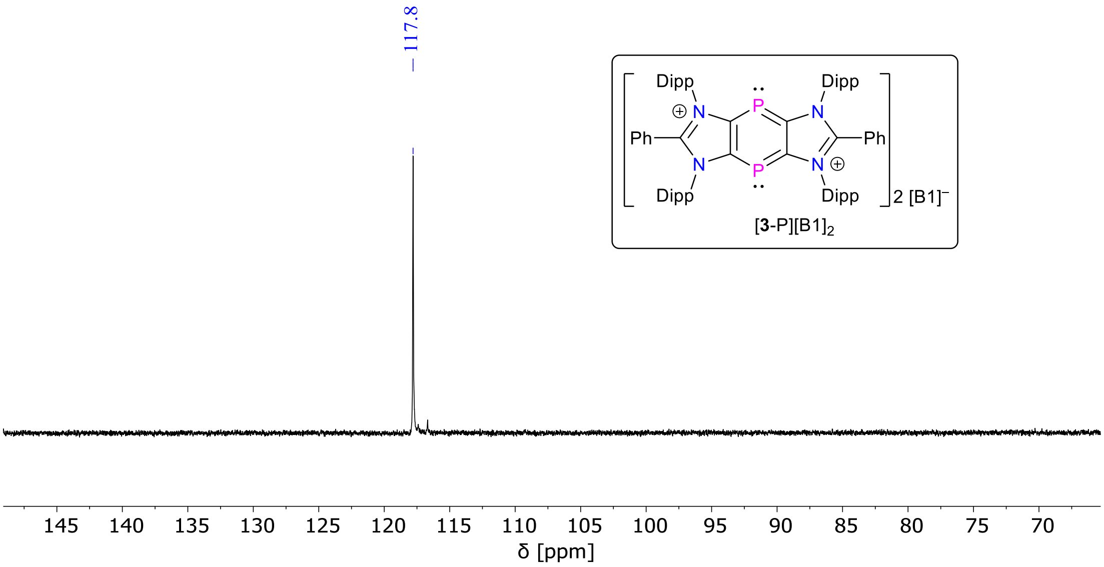

Figure S6. ^{31}P NMR (202 MHz, THF-*d*8, 298 K) of [**3**-P][B1]$_2$.

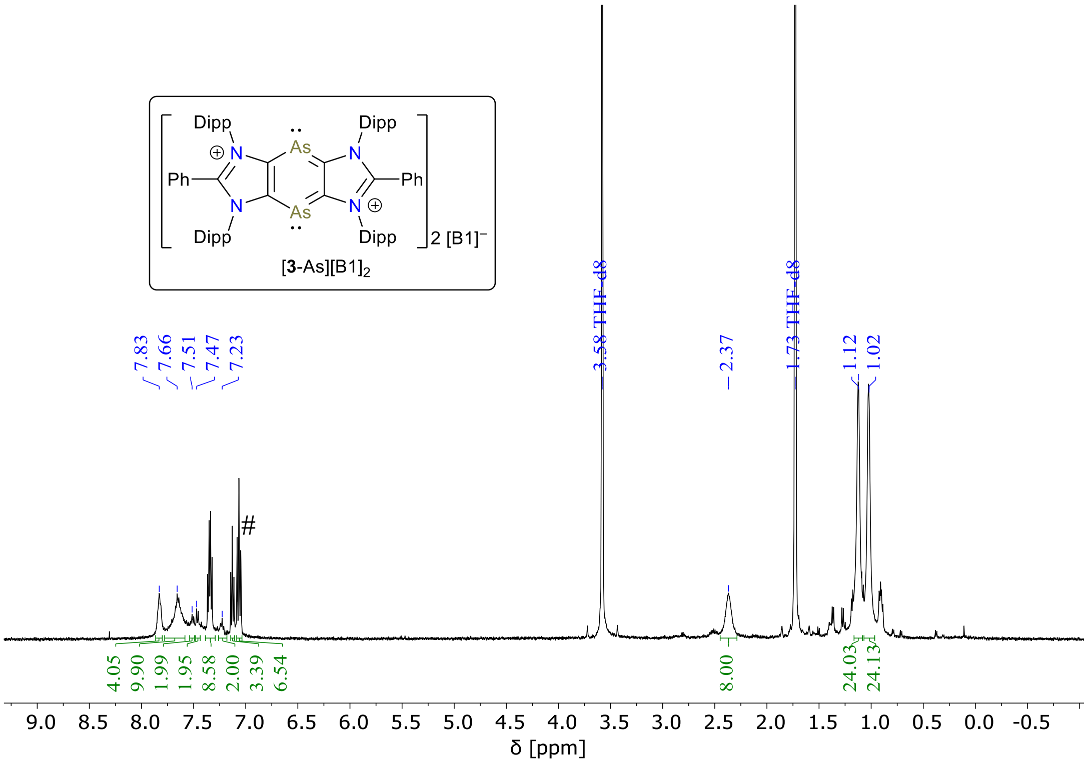

Figure S7. ^{1}H NMR (500 MHz, THF-*d*8, 298 K) of [**3**-As][B1]$_2$. $^{\#}C_6H_5F$.

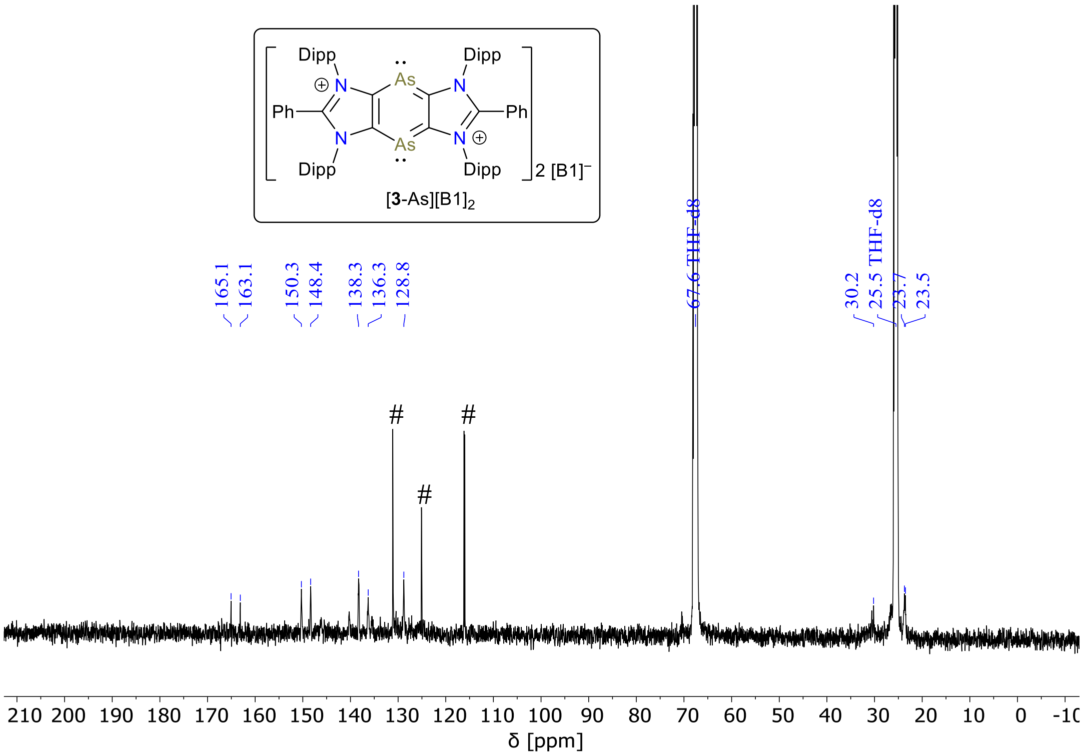

Figure S8. $^{13}C\{^{1}H\}$ NMR (125 MHz, THF-*d*8, 298 K) of [**3**-As][B1]$_2$. $^{\#}C_6H_5F$.

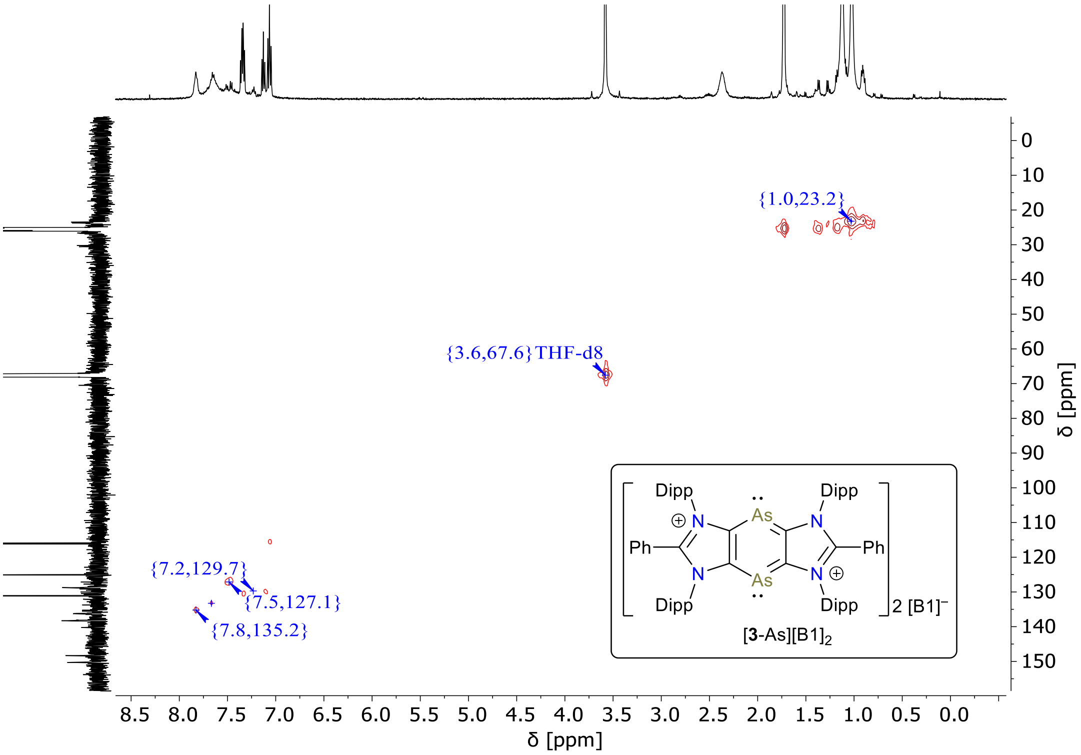

Figure S9. ^{1}H-$^{13}C\{^{1}H\}$ HMQC NMR (500/125 MHz, THF-*d*8, 298 K) of [**3**-As][B1]$_2$.

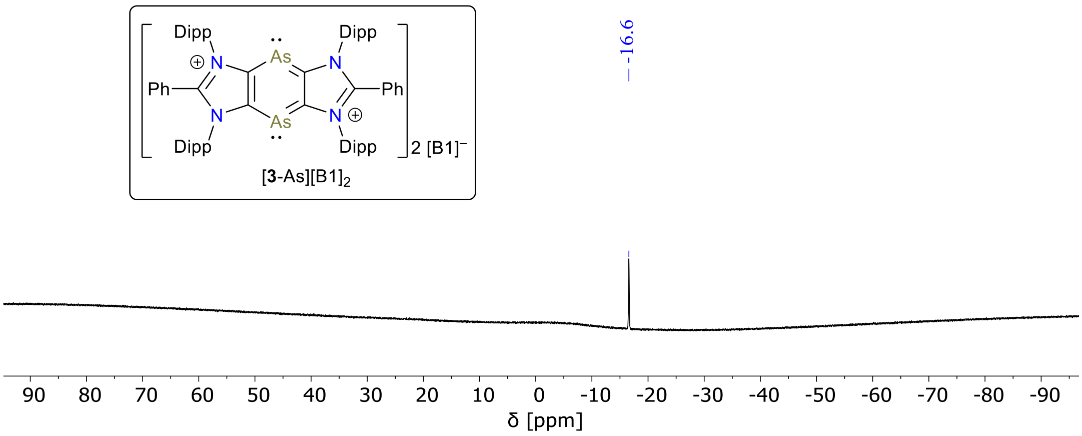

Figure S10. $^{11}B\{^{1}H\}$ NMR (160 MHz, THF-*d*8, 298 K) of [**3**-As][B1]$_2$.

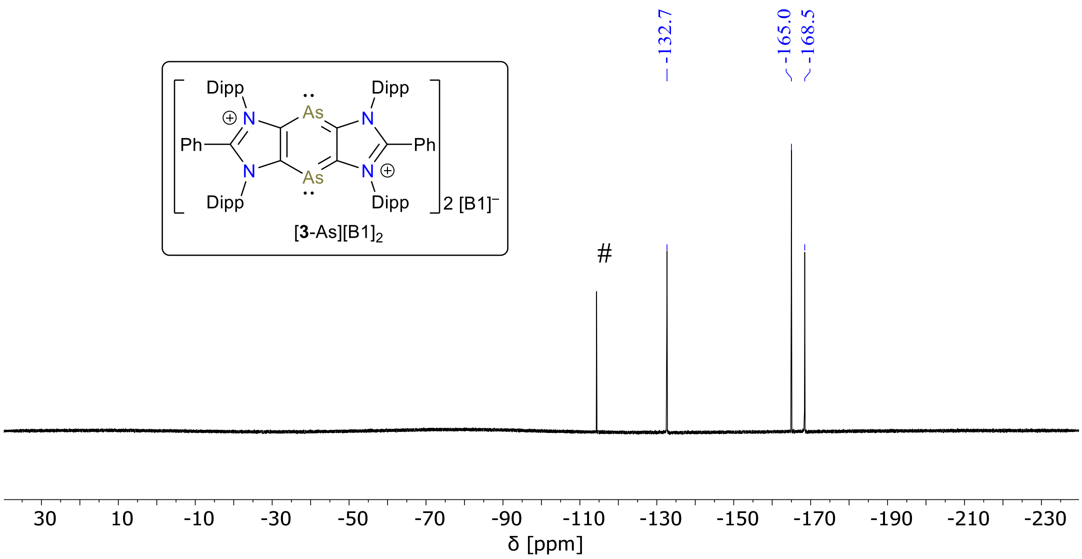

Figure S11. $^{19}F\{^{1}H\}$ NMR (471 MHz, THF-*d*8, 298 K) of [**3**-As][B1]$_2$. $^{\#}C_6H_5F$.

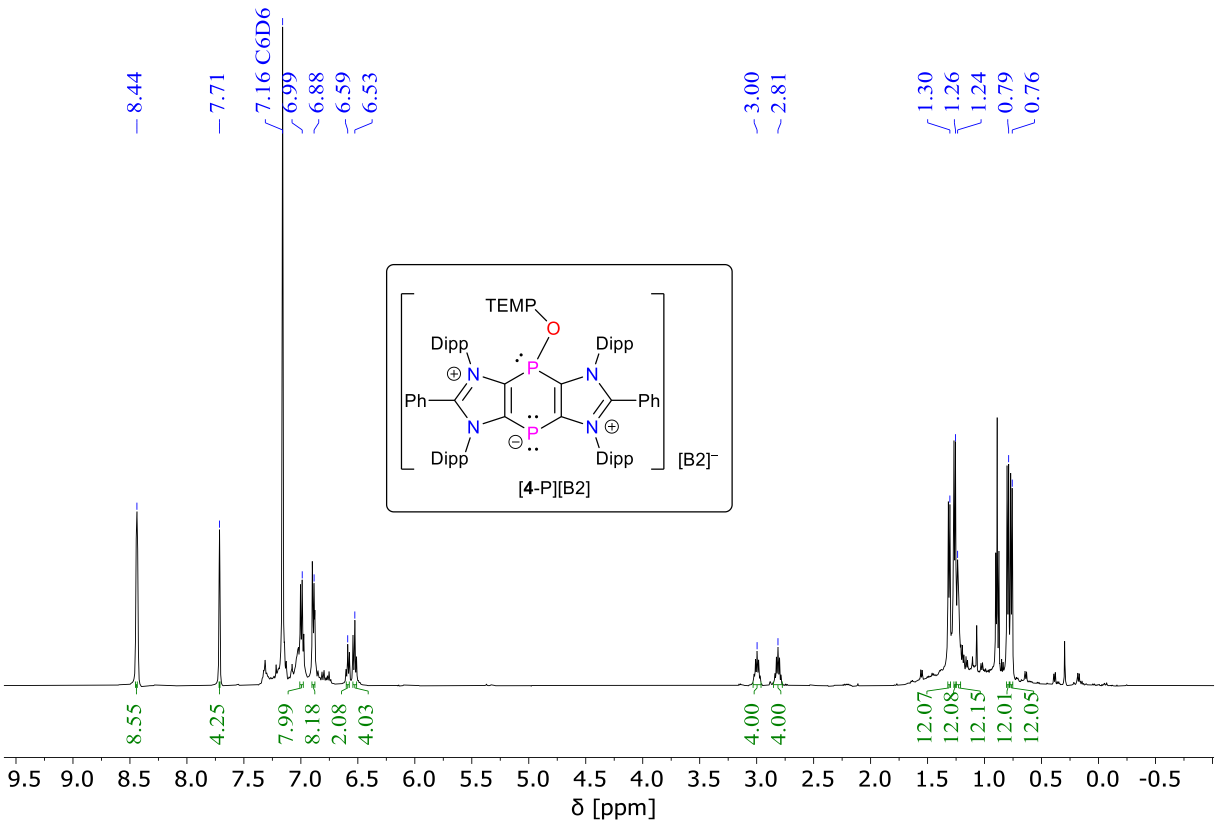

Figure S12. ^{1}H NMR (500 MHz, C_6D_6, 298 K) of [**4**-P][B2].

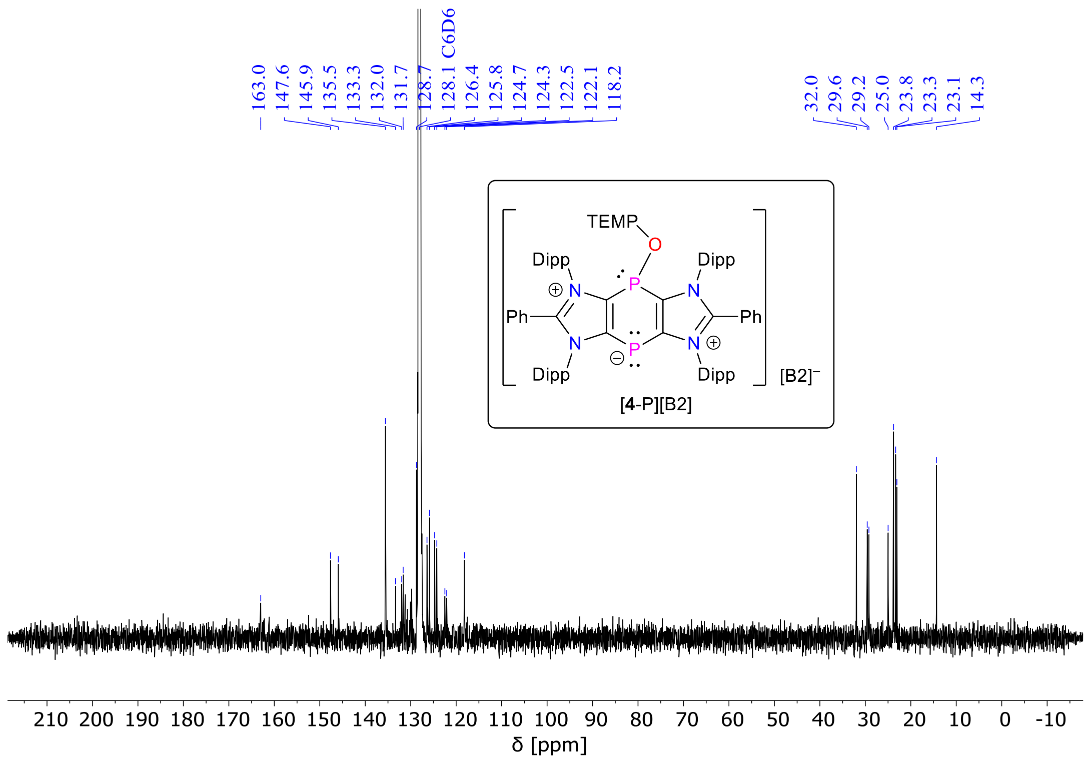

Figure S13. $^{13}C\{^{1}H\}$ NMR (125 MHz, C_6D_6, 298 K) of [**4**-P][B2].

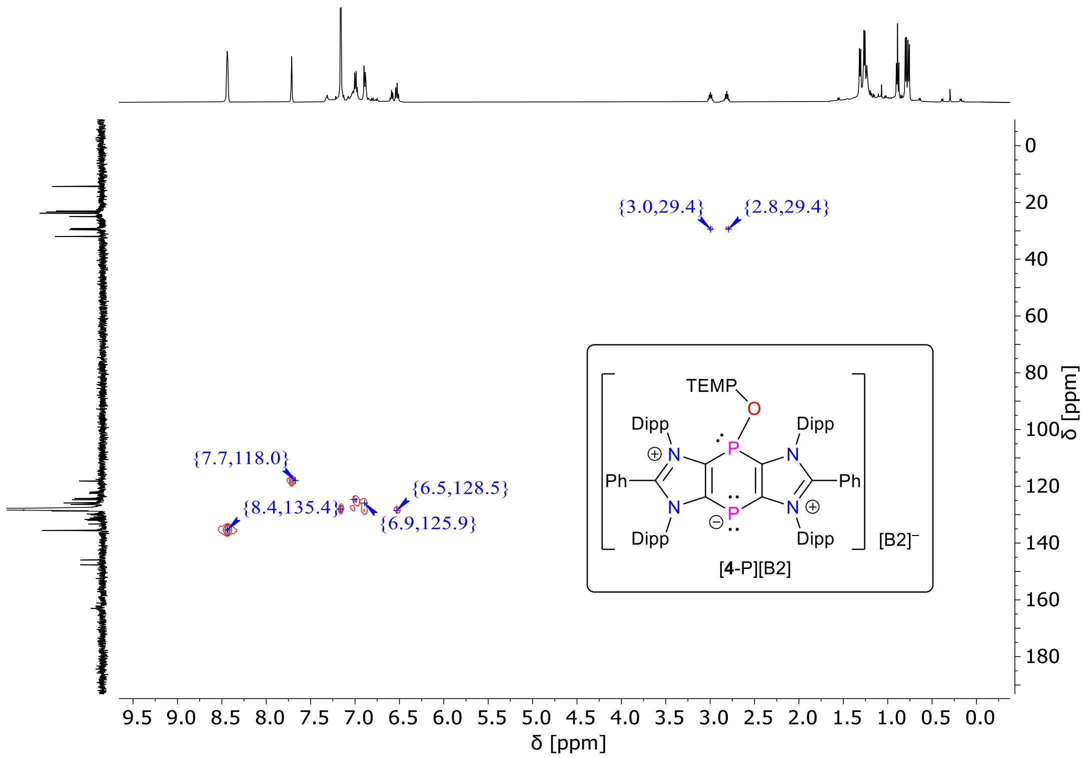

Figure S14. ^{1}H-$^{13}C\{^{1}H\}$ HMQC NMR (500/125 MHz, C_6D_6, 298 K) of [**4**-P][B2].

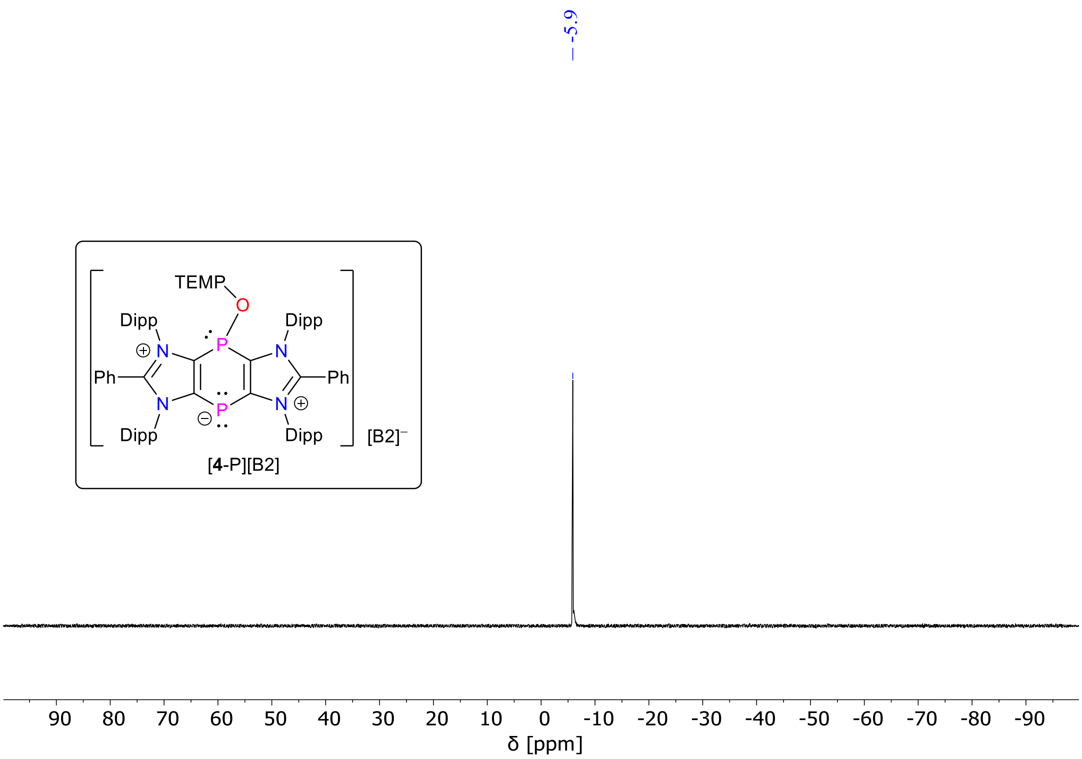

Figure S15. $^{11}B\{^{1}H\}$ NMR (160 MHz, C_6D_6, 298 K) of [**4**-P][B2].

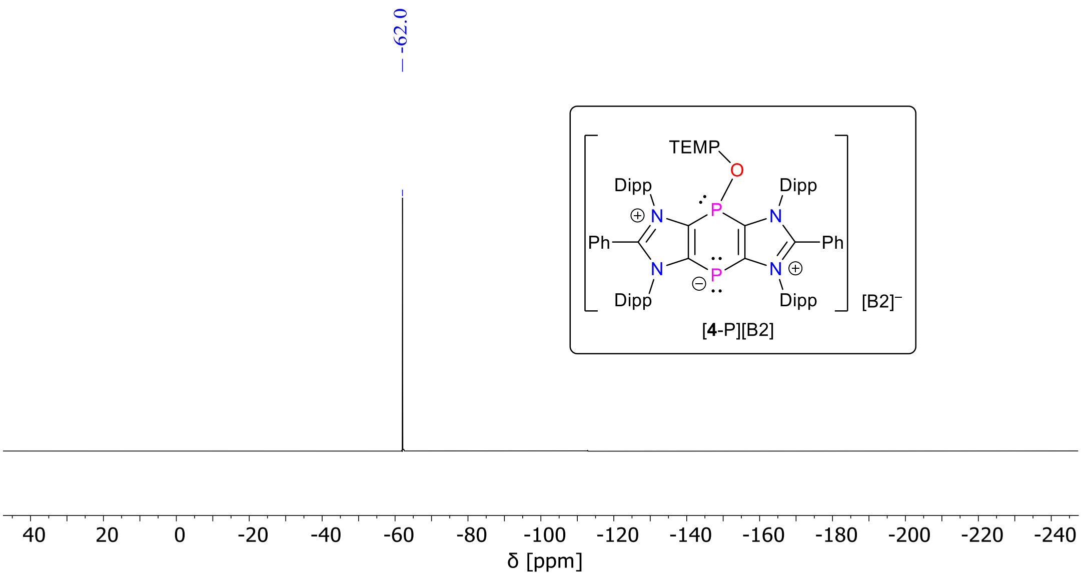

Figure S16. $^{19}F\{^{1}H\}$ NMR (471 MHz, C_6D_6, 298 K) of [**4**-P][B2].

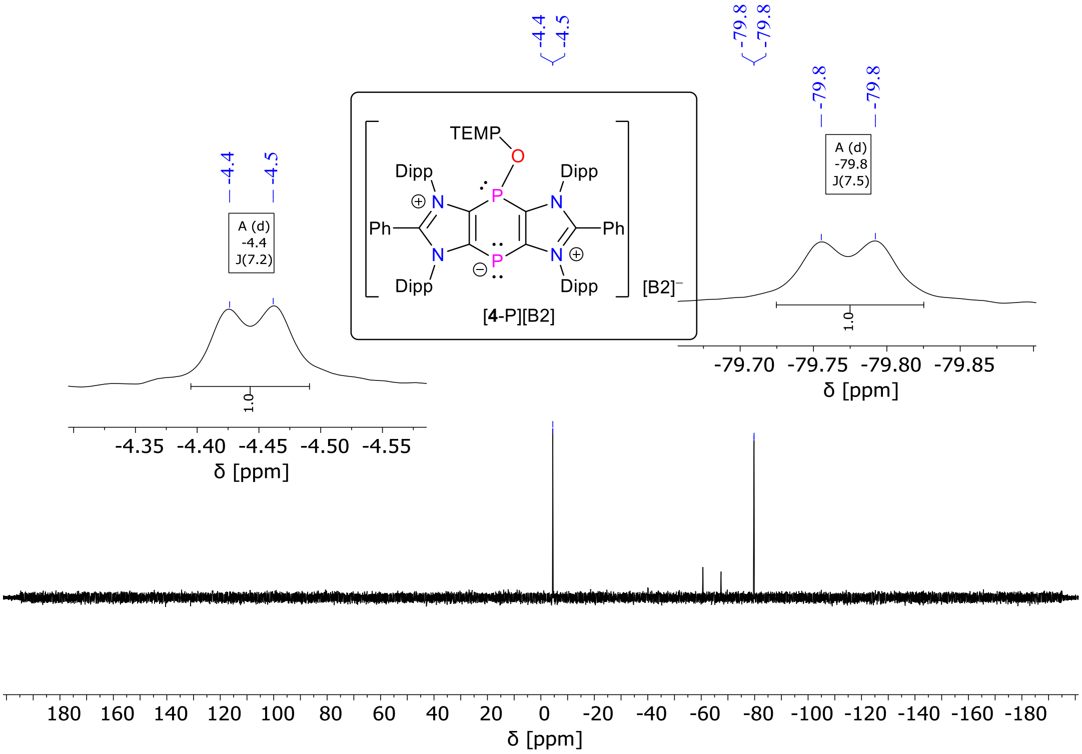

Figure S17. ^{31}P NMR (202 MHz, C_6D_6, 298 K) of [**4**-P][B2].

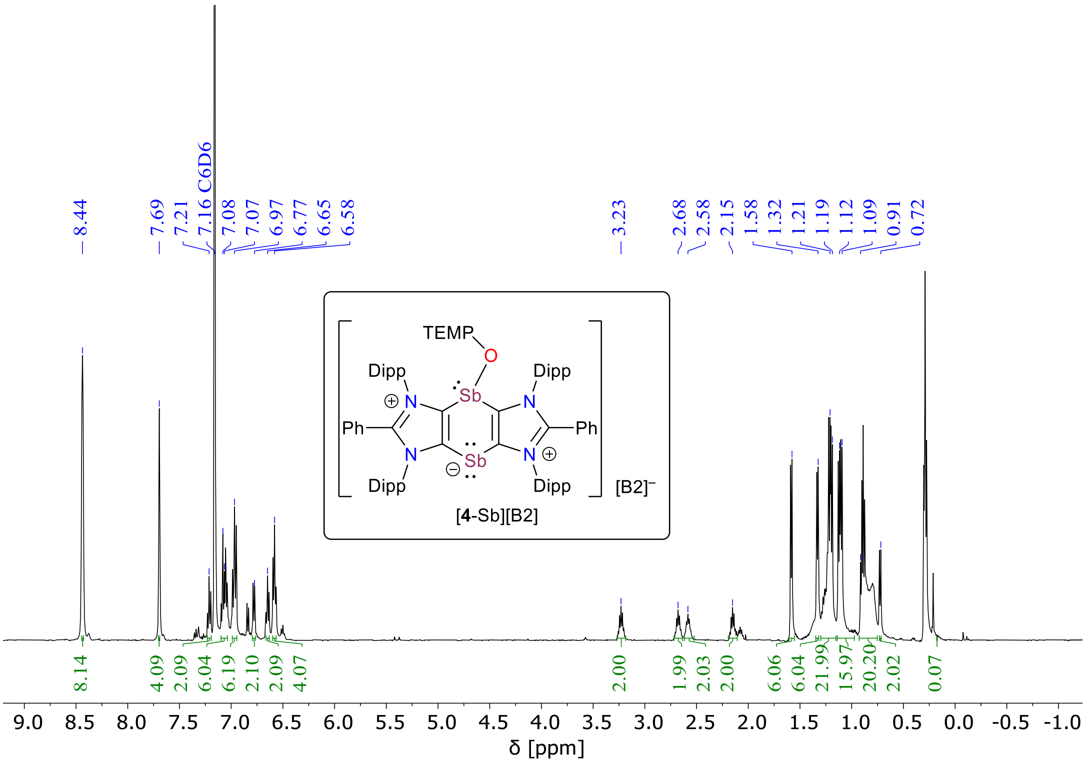

Figure S18. ^{1}H NMR (500 MHz, C_6D_6, 298 K) of [**4**-Sb][B2].

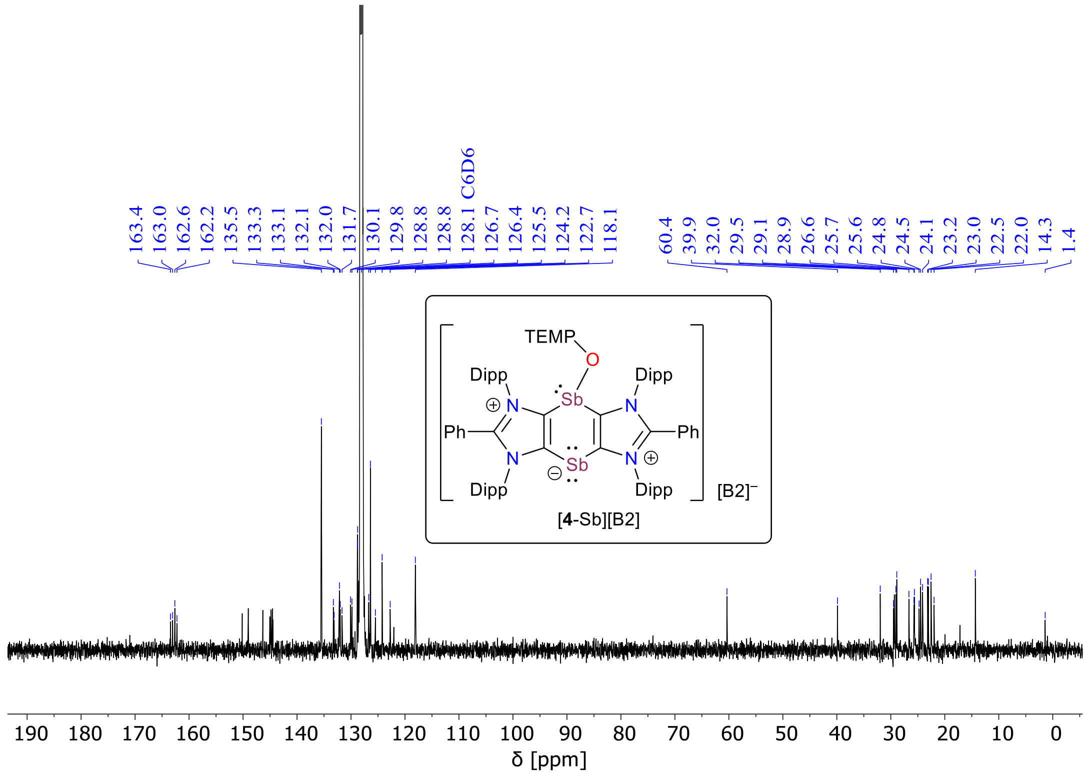

Figure S19. $^{13}C\{^{1}H\}$ NMR (125 MHz, C_6D_6, 298 K) of [**4**-Sb][B2].

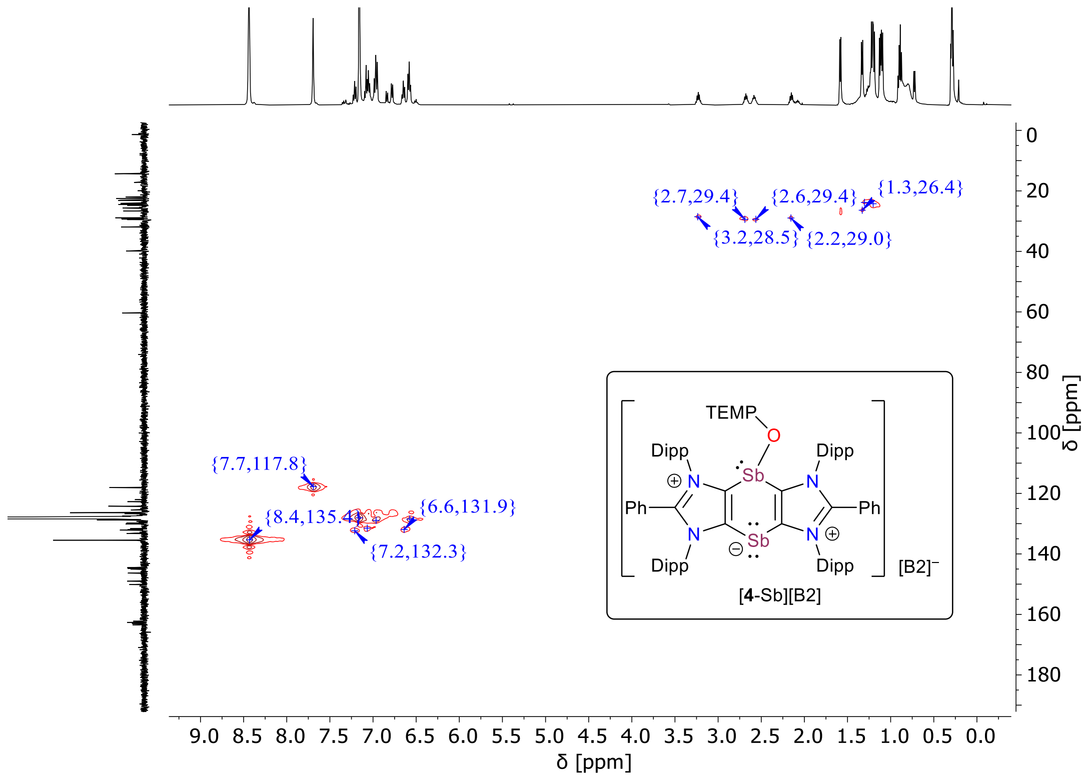

Figure S20. ^{1}H-$^{13}C\{^{1}H\}$ HMQC NMR (500/125 MHz, C_6D_6, 298 K) of [**4**-Sb][B2].

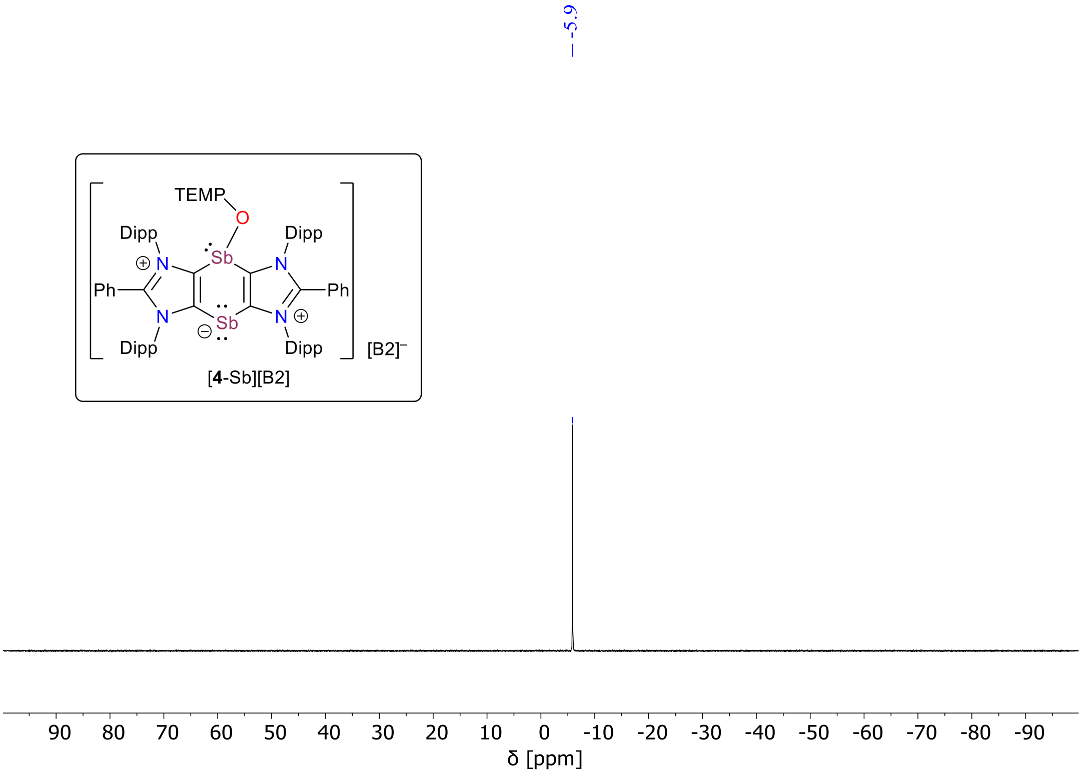

Figure S21. $^{11}B\{^{1}H\}$ NMR (160 MHz, C_6D_6, 298 K) of [**4**-Sb][B2].

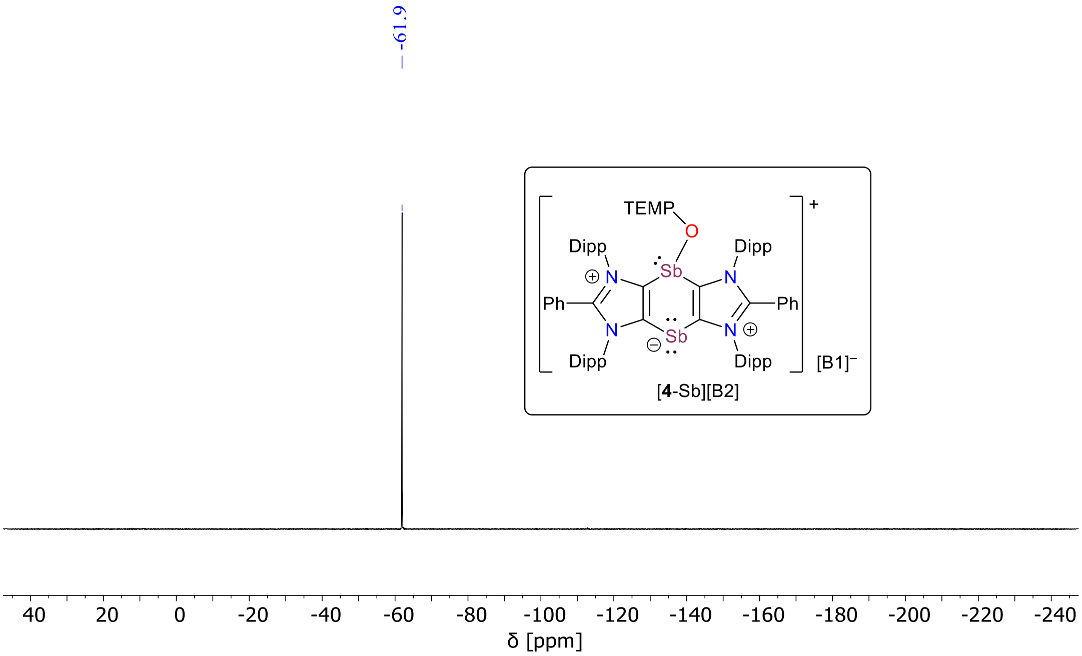

Figure S22. $^{19}F\{^{1}H\}$ NMR (471 MHz, C_6D_6, 298 K) of [**4**-Sb][B2].

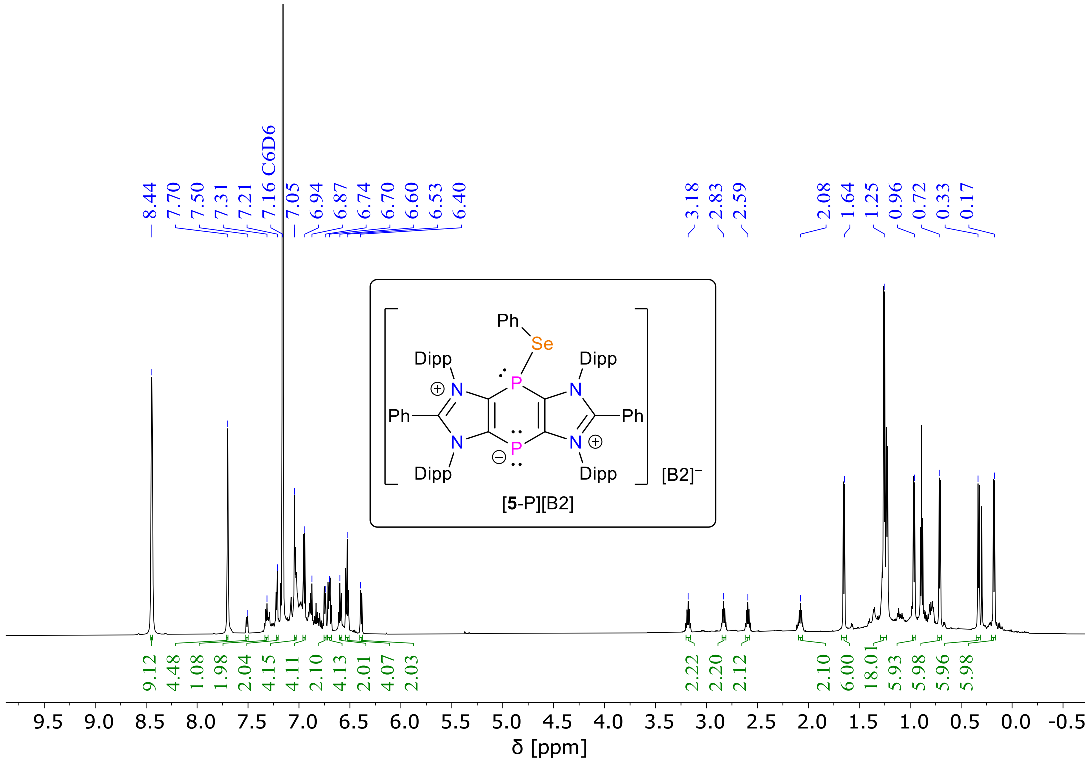

Figure S23. ^{1}H NMR (600 MHz, C_6D_6, 298 K) of [**5**-P][B2] with 1.1 eq of B2.

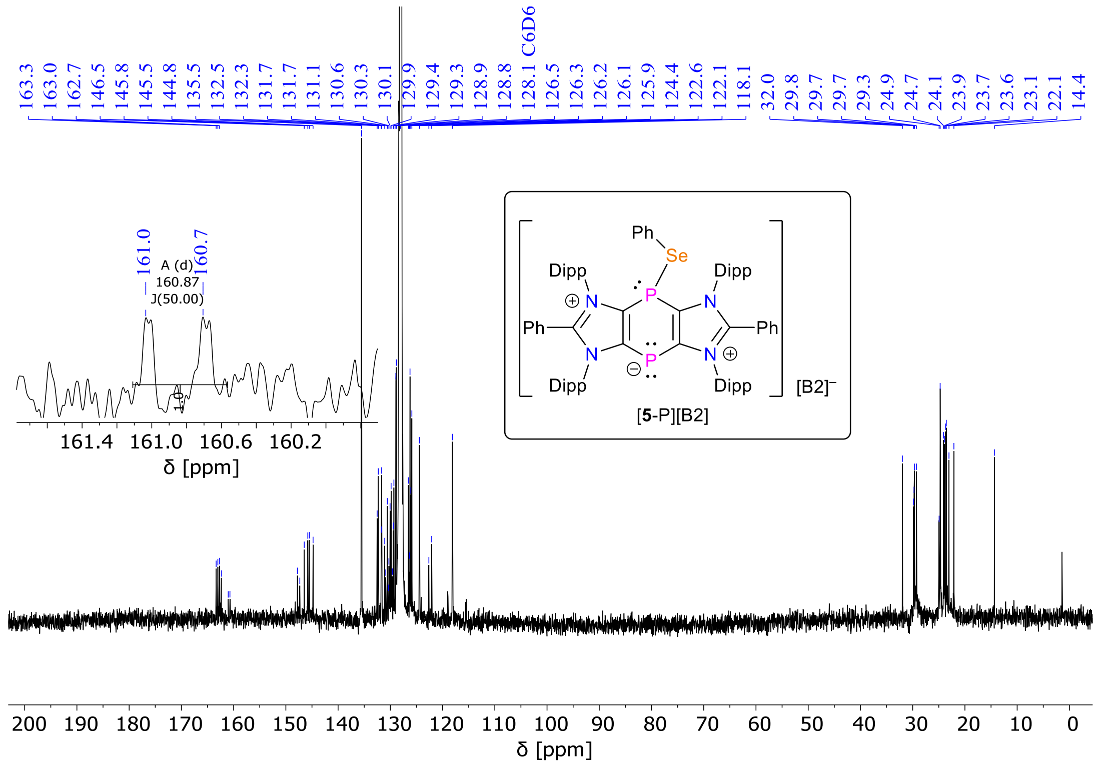

Figure S24. $^{13}C\{^{1}H\}$ NMR (150 MHz, C_6D_6, 298 K) of [**5**-P][B2].

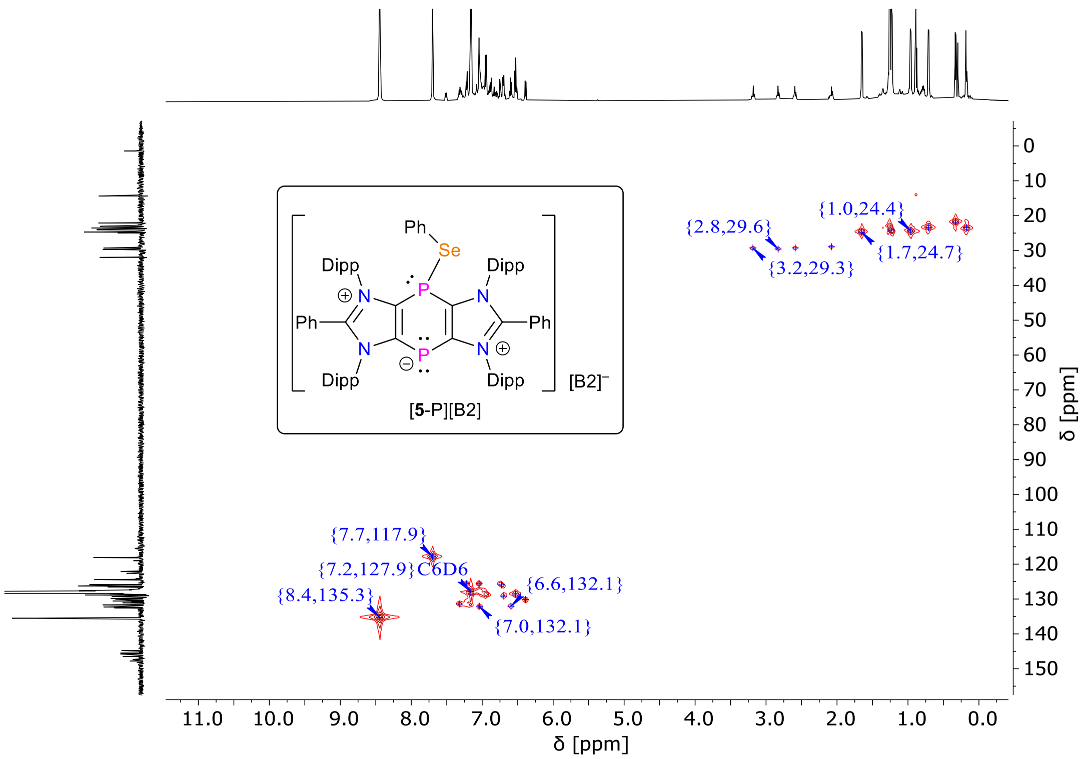

Figure S25. ^{1}H-^{13}C{^{1}H} HMQC NMR (500/125 MHz, C_6D_6, 298 K) of [**5**-P][B2].

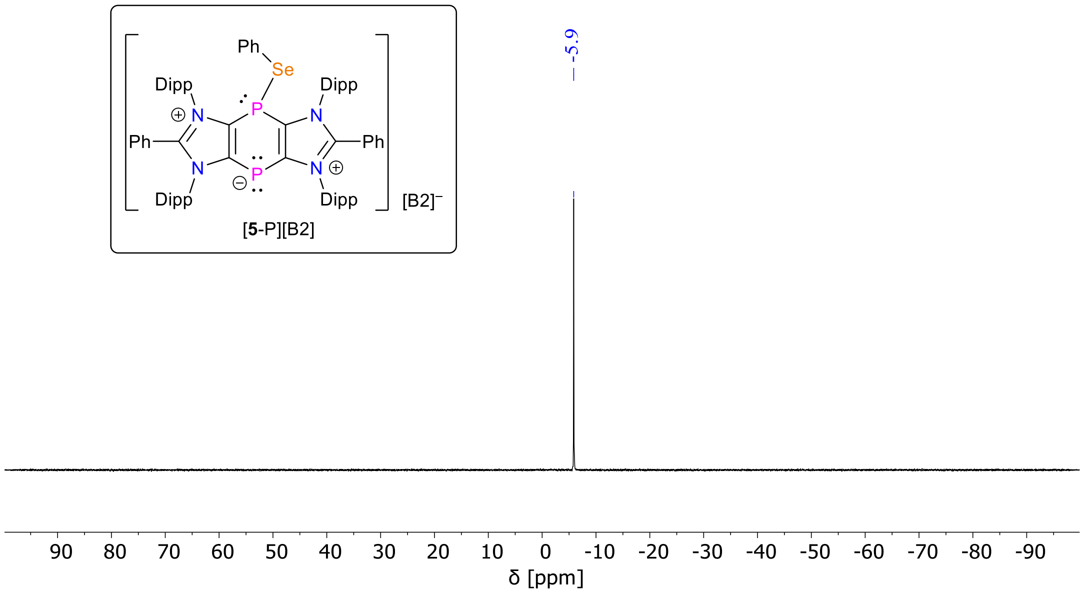

Figure S26. ^{11}B{^{1}H} NMR (160 MHz, C_6D_6, 298 K) of [**5**-P][B2].

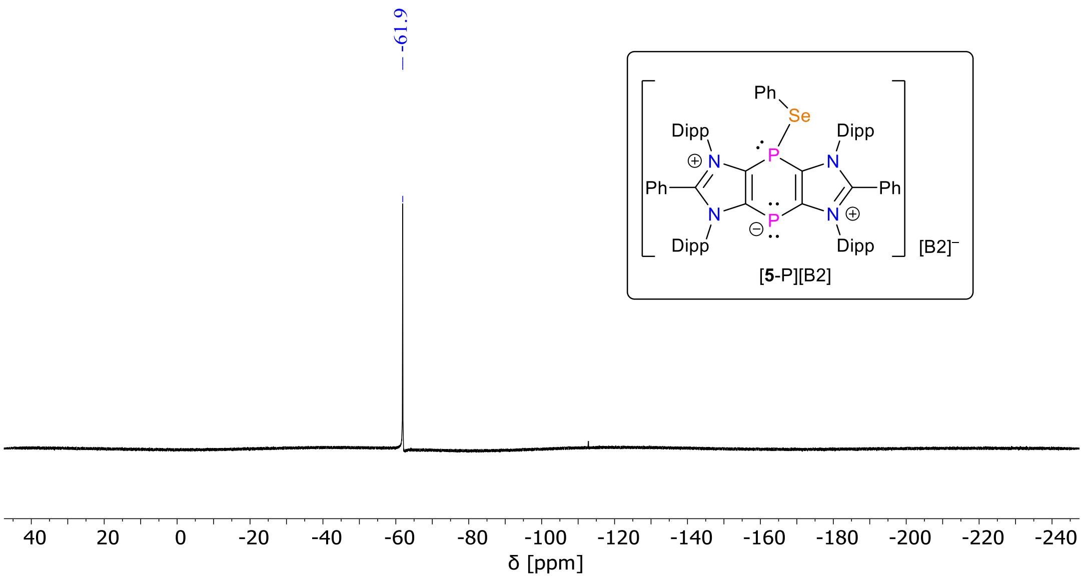

Figure S27. $^{19}F\{^{1}H\}$ NMR (471 MHz, C_6D_6, 298 K) of [**5**-P][B2].

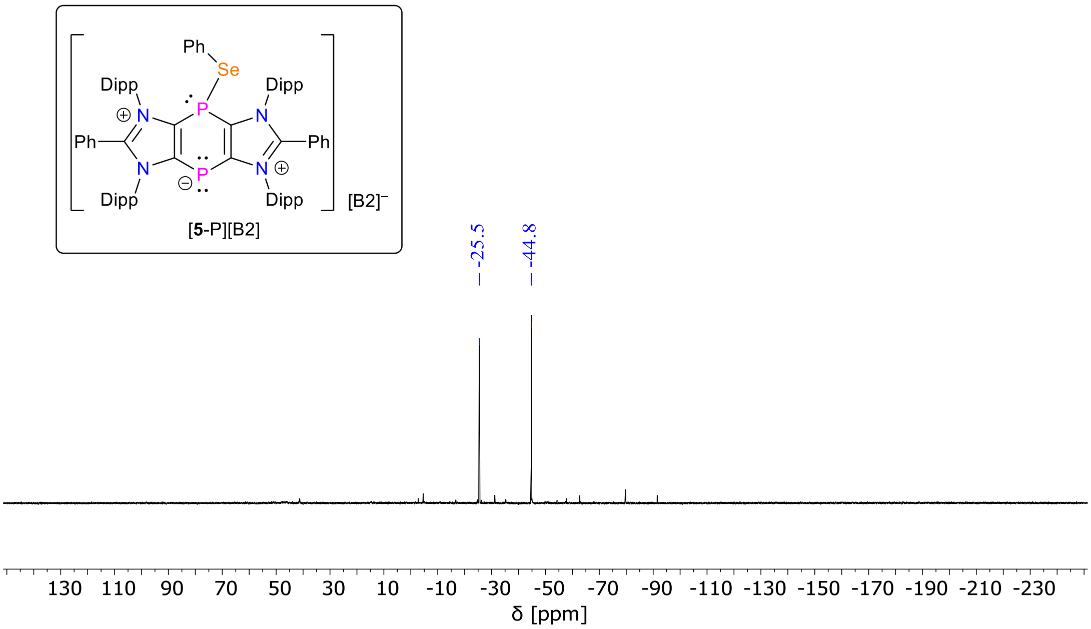

Figure S28. ^{31}P NMR (202 MHz, C_6D_6, 298 K) of [**5**-P][B2].

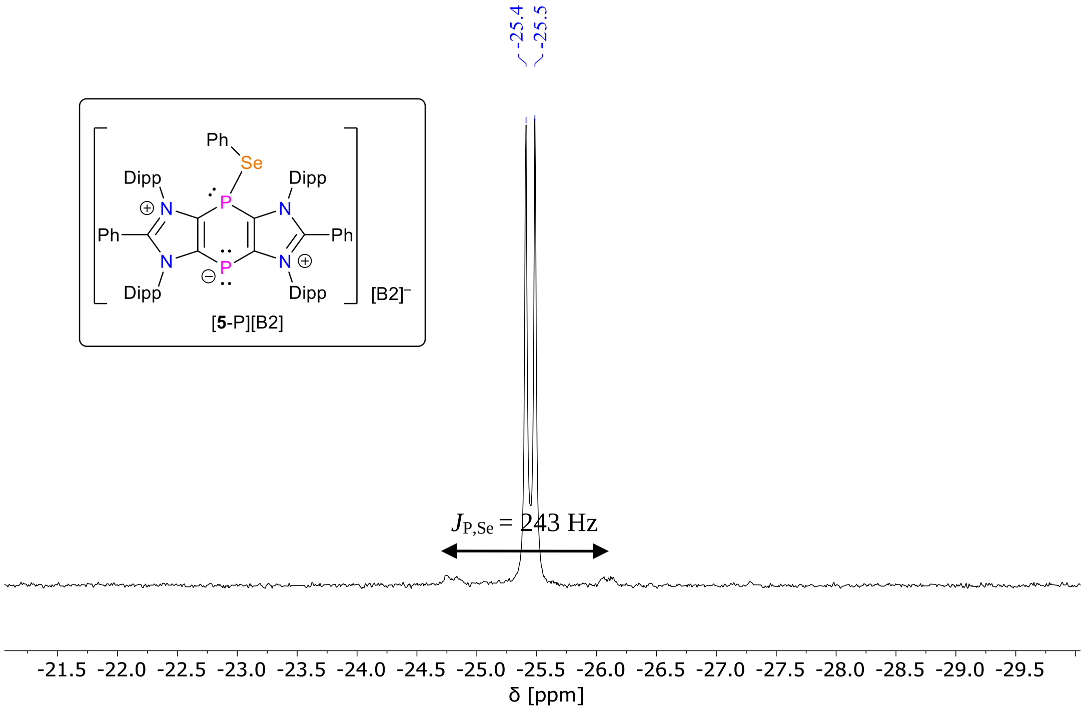

Figure S29. ^{31}P NMR (202 MHz, C_6D_6, 298 K) of [**5**-P][B2] zoom.

UV-Vis. Spectra

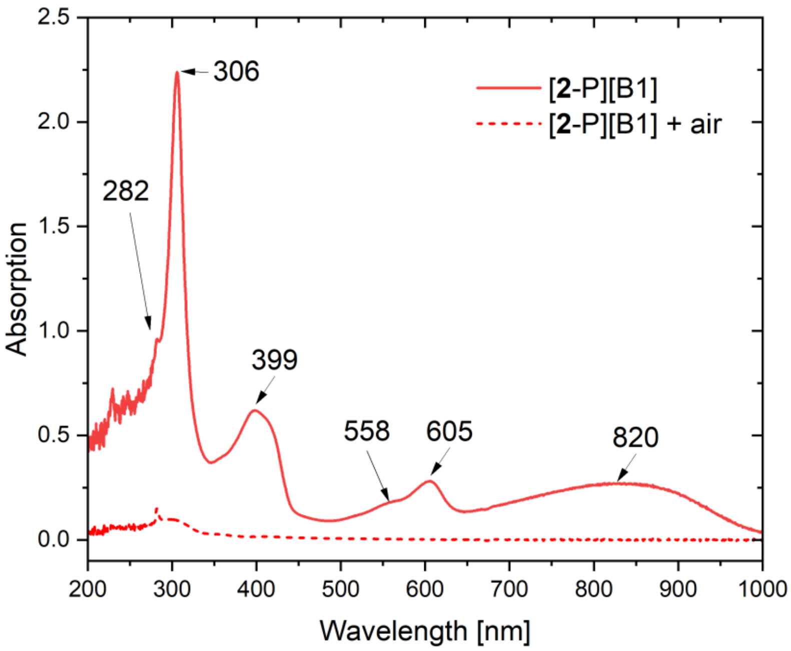

Figure S30. UV-visible spectra of [**2**-P][B1] measured in toluene (120µM). Observed absorption bands and calculated molar extinction coefficients ε (L $mol^{-1}cm^{-1}$): [λ_1 = 282 nm (8333); λ_2 = 306 nm (18333); λ_3 = 399 nm (5000); λ_4 = 558 nm (1417); λ_5 = 605 nm (2250); λ_6 = 820 nm (2167)].

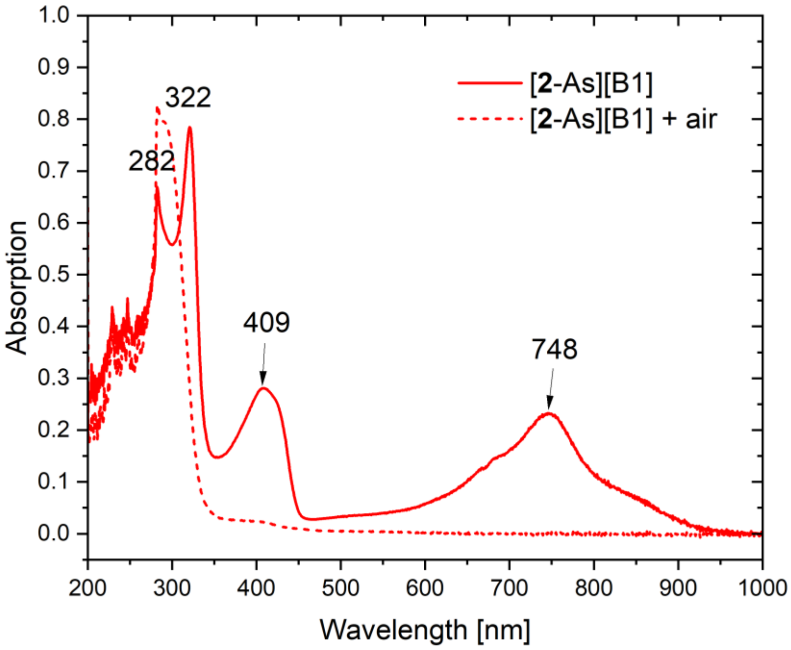

Figure S31. UV-visible spectra of [**2**-As][B1] (63µM) measured in toluene. Observed absorption bands and calculated molar extinction coefficients ε (L mol^{-1} cm^{-1}): [λ_1 = 282 nm (10476); λ_2 = 322 nm (12380); λ_3 = 409 nm (4444); λ_4 = 748 nm (3651)].

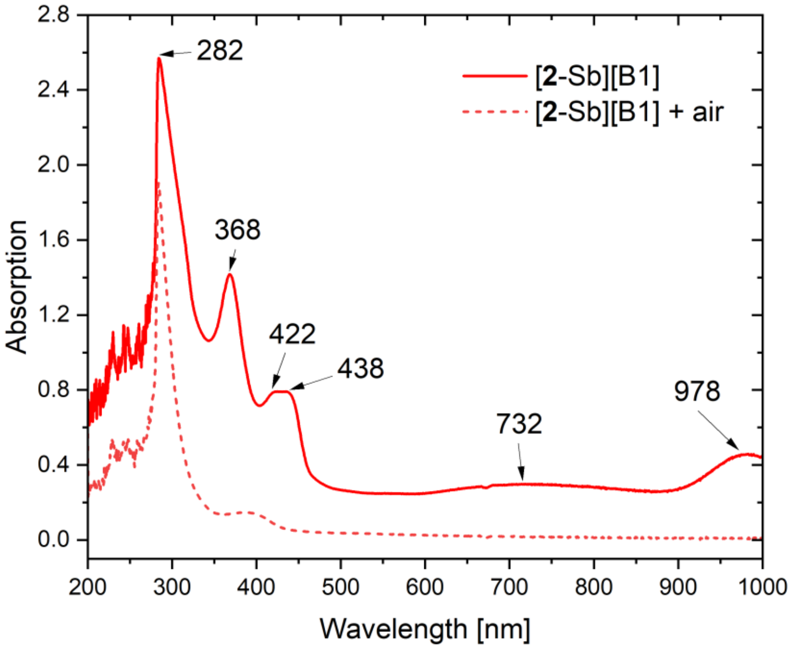

Figure S32. UV-visible spectra of [**2**-Sb][B1] measured in toluene (200 µM). Observed absorption bands and calculated molar extinction coefficients ε (L mol^{-1}cm^{-1}): [λ_1 = 282 nm (12850); λ_2 = 368 nm (7050); λ_3 = 422 nm (3950); λ_4 = 438 nm (3950); λ_5 = 732 nm (1500); λ_6 = 978 nm (2300)].

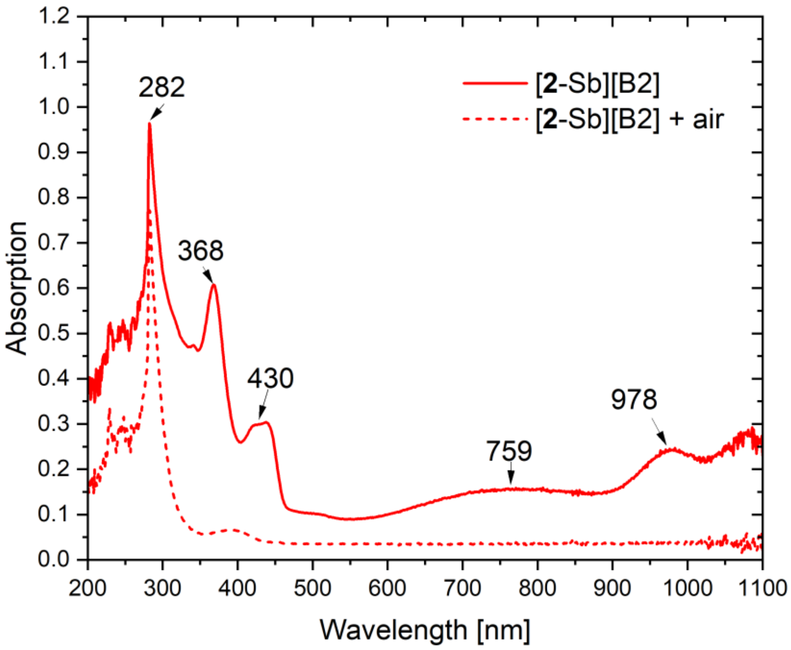

Figure S33. UV-visible spectra of [**2**-Sb][B2] measured in toluene (32 µM). Observed absorption bands and calculated molar extinction coefficients ε (L mol^{-1}cm^{-1}): λ_1 = 282 nm (30000); λ_2 = 368 nm (19063); λ_3 = 430 nm (9375); λ_4 = 759 nm (5000); λ_5 = 978 nm (7500)].

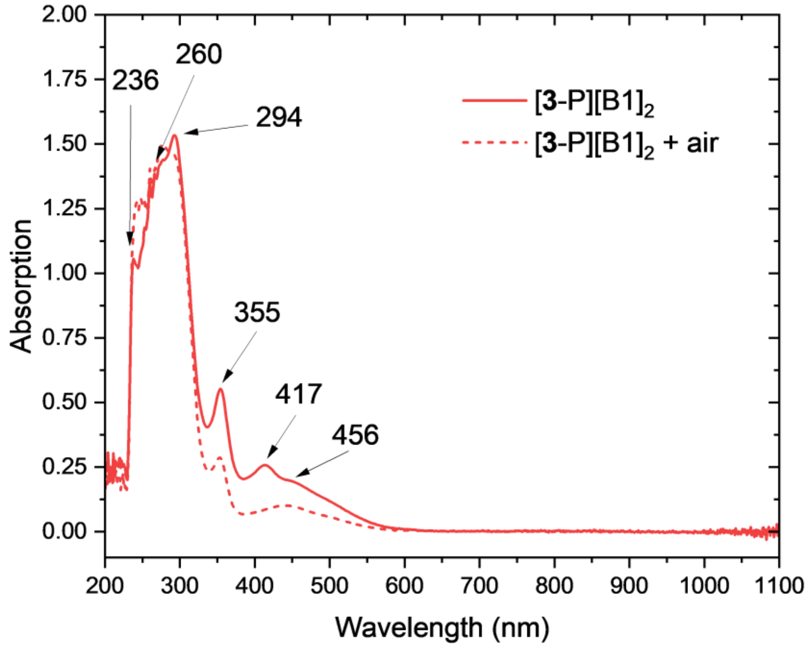

Figure S34. UV-visible spectra of [**3**-P][B1]$_2$ measured in THF. Observed absorption bands and calculated molar extinction coefficients ε (L mol^{-1} cm^{-1}) for 43 μM: [λ_1 = 236 nm (24651); λ_2 = 260 nm (30000); λ_3 = 294 nm (35249); λ_4 = 355 nm (12791); λ_5 = 417 nm (5814); λ_6 = 456 nm (4419)].

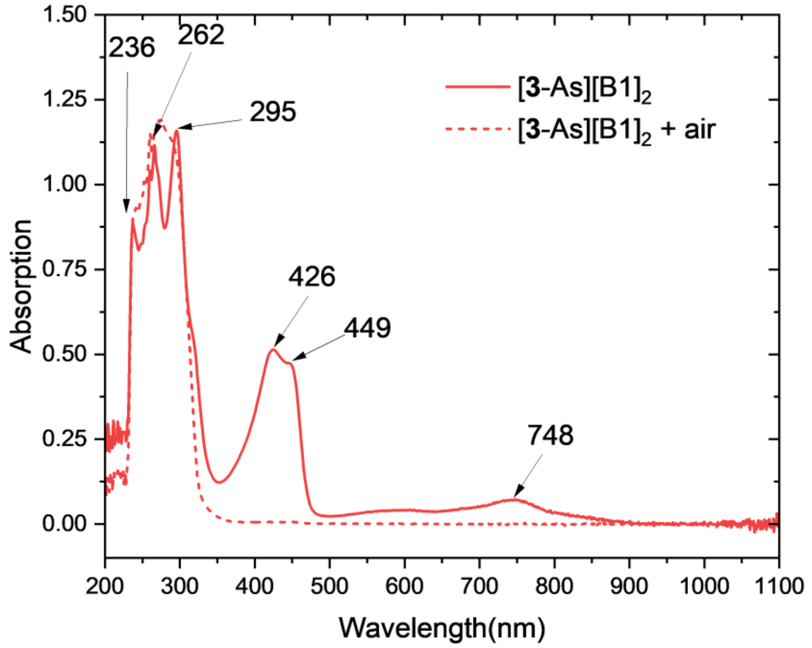

Figure S35. UV-visible spectra of [**3**-As][B1]$_2$ measured in THF. Observed absorption bands and calculated molar extinction coefficients ε (L mol^{-1} cm^{-1}) for 59 μM: [λ_1 = 236 nm (15254); λ_2 = 262 nm (18814); λ_3 = 295 nm (19492); λ_4 = 426 nm (8814); λ_5 = 449 nm (7966); λ_6 = 748 nm (1186)].

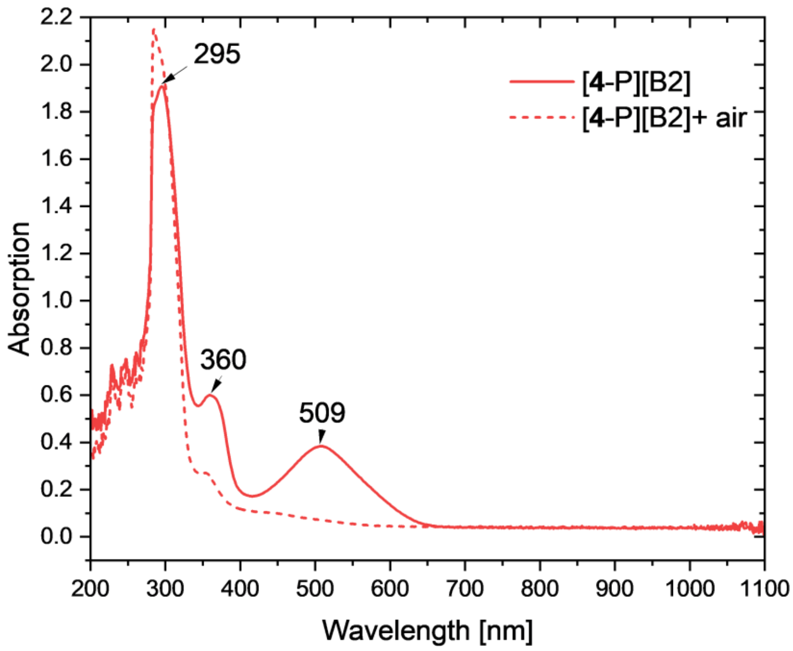

Figure S36. UV-visible spectra of [**4**-P][B2] measured in toluene (85 µM). Observed absorption bands and calculated molar extinction coefficients ε (L mol^{-1} cm^{-1}): λ_1 = 295 nm (22471); λ_2 = 360 nm (7059); λ_3 = 509 nm (4471)].

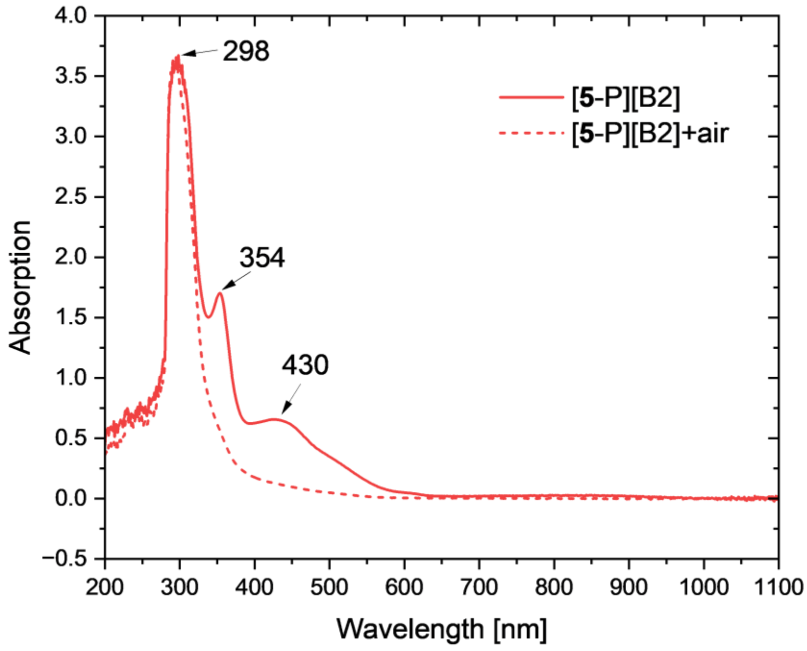

Figure S37. UV-visible spectra of [**5**-P][B2] measured in toluene (100 µM). Observed absorption bands and calculated molar extinction coefficients ε (L mol^{-1} cm^{-1}): λ_1 = 298 nm (36400); λ_2 = 354 nm (16800); λ_3 = 430 nm (6500)].

Cyclic Voltammetry

Cyclic voltammetry (CV) experiments were carried out using a PGSTAT204 electrochemical workstation (METROHM). All experiments were carried out under an atmosphere of argon in a degassed and anhydrous *o*-difluorobenzene (*o*-DFB) solution of **1**-E containing [^{n}Bu$_4$N][PF$_6$] (0.1 M) at scan rates of 50, 100, 250, 500, and 1000 mV/s. The setup consisted of a glassy carbon working electrode (surface area = 0.04 cm^2), a glassy carbon counter electrode, and a silver wire as the pseudo-reference electrode. The CV of pure ferrocene (in *o*-DFB, 0.1 M *n*Bu$_4$N[PF$_6$], 100 mV/s) displayed a characteristic redox wave at $E_{1/2}$ = 0.44 V. However, the presence of **1**-E interfered with the measurements, as no redox wave for the ferrocene/ferrocenium (Fc/Fc$^+$) couple was observed upon adding ferrocene to the solution of **1**-E. Instead, a spike appeared at E_{pa} > 1.5 V in all cases. This may result from a chemical reaction with **1**-E, leading to the deposition of an unknown substance on the working electrode upon ferrocene addition.

(a) (b)

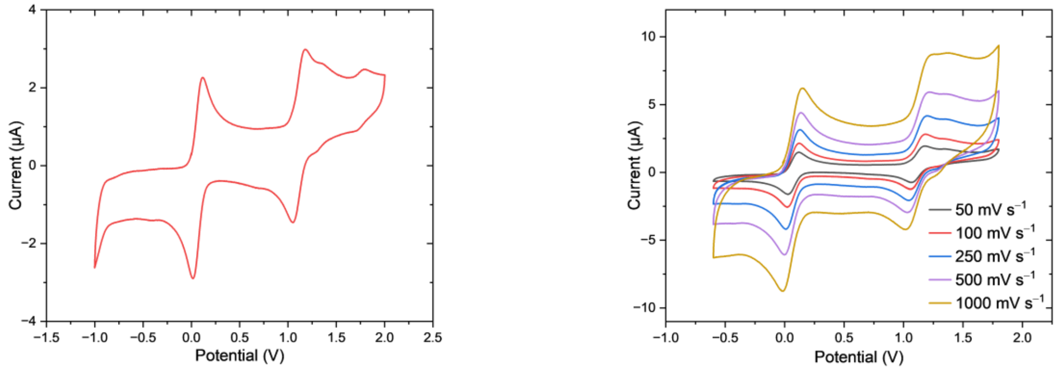

Figure S38. (a) CV of **1**-P (0.81 mM) at 100 mV/s ($E_{1/2}$ = 0.07, 1.12 V *vs.* Ag$^+$/Ag). (b) Stacks of CVs of **1**-P at different scan rates.

(a) (b)

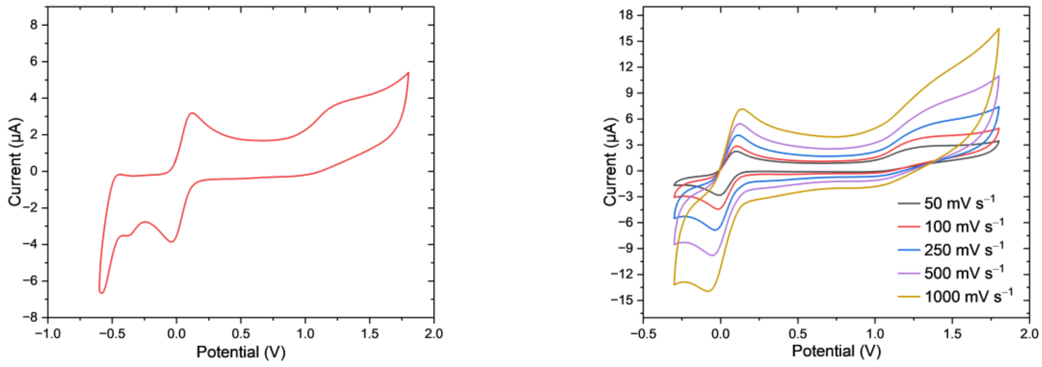

Figure S39. (a) CV of **1**-As (4.27 mM) at 100 mV/s ($E_{1/2}$ = 0.08 V; E_{pa} = 1.19 V *vs.* Ag$^+$/Ag). (b) Stacks of CVs of **1**-As at different scan rates.

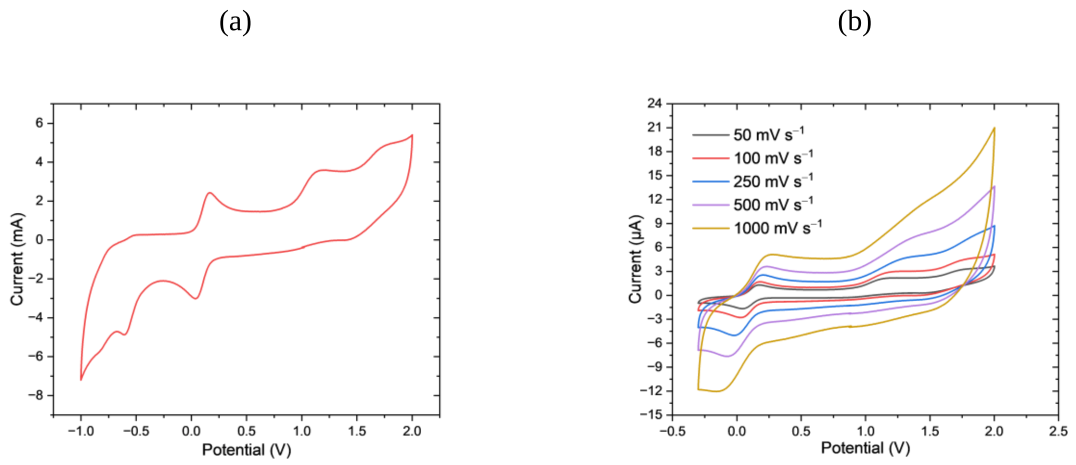

Figure S40. (a) CV of **1**-Sb (2.39 mM) at 100 mV/s ($E_{1/2}$ = 0.10 V; E_{pa} = 1.16 V, 1.72 V *vs.* Ag^+/Ag). (b) Stacks of CVs of **1**-Sb at different scan rates.

EPR Spectroscopy

CW-EPR spectra were recorded in MeTHF solutions at room temperature (298 K) on a Bruker EMXNano X-band spectrometer. Simulations of the EPR spectra were performed by iteration of the isotropic g-values, hyperfine coupling interactions and line widths using EasySpin (version 6.0.6)[8] via the cwEPR GUI (version v3.6).[9] The spectra of [**2**-P][B1], [**2**-As][B1] and [**2**-Sb][B1] were simulated with two equivalent P, As and Sb nuclei, respectively. Simulation of the solution spectra recorded at RT required the usage of a restricted tumbling model using the t_{corr} parameter in the “garlic” module.

Geometries (full atom model) were optimized with Turbomole (version 7.8.1) at the BP86, def2 TZVP, disp3(zero) level. Subsequently, EPR properties were calculated at the b3-lyp, b3 lyp, x2c-TZVPPall-2c, disp3(zero) level using relativistic two-component (spin-orbit) single point calculations ($rx2c, $rlocal, $finnuc), using the “gtensprep.sh -msnso -hfc -efg” shell script procedure described in the Turbomole manual. DFT calculated EPR parameters were obtained with the Turbomole program package.[10] Geometries were optimized at the UDFT/bp86 level. We used the def2-TZVP basis set for the geometry optimizations. Grimme’s dispersion corrections (version 3, disp3, ‘zero damping’) were used to include Van der Waals interactions. EPR parameters were calculated with a separate SCF calculation at the b3-lyp level with relativistic rx2c two-component calculations with the x2c-TZVPall-2c basis set. A comparison of the experimental EPR parameter derived from spectral simulation and the DFT calculated values is shown in Table S1.

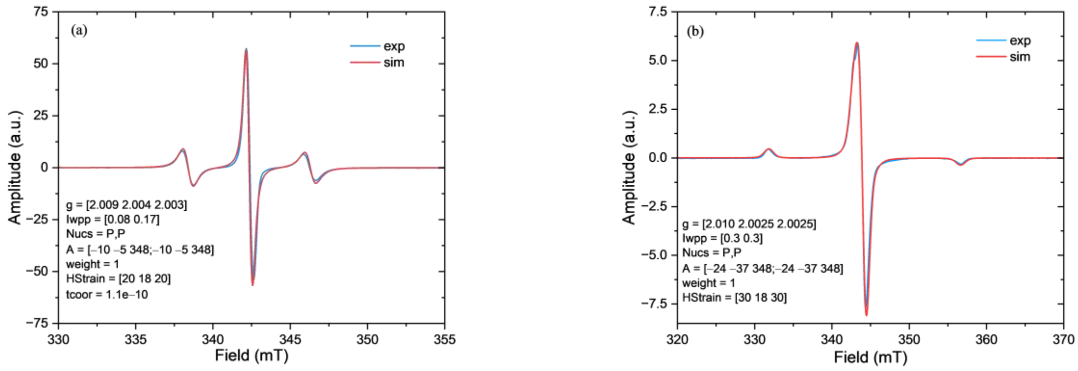

Figure S41. (a): Isotropic X-band EPR spectrum of [**2**-P][B1] recorded at RT in Me-THF. Mw. Freq=9.6094 GHz, Mod. Amp 4 G. Mw. Power = 1 mW. (b): X-band EPR spectrum of [**2**-P][B1] recorded in a frozen Me-THF solution at 20 K. Mw. Freq=9.6491 GHz, Mod. Amp 4 G. Mw. Power = 2 mW. The simulated spectra (red) were obtained with the simulation parameters shown in the insets, using “garlic” (RT) or “pepper” (frozen solution) modules. DFT calculated EPR parameters were used as an initial guess for the simulations.

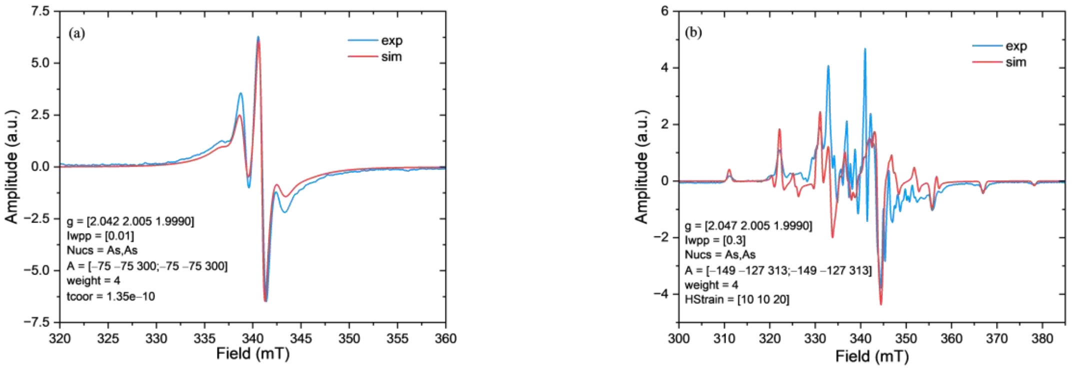

Figure S42. (a): Isotropic X-band EPR spectrum of [**2**-As][B1]. Mw. Freq=9.6094 GHz, Mod. Amp 4 G. Mw. Power = 1 mW. (b): X-band EPR spectrum of [**2**-As][B1] recorded in a frozen Me-THF solution at 20 K. Mw. Freq= 9.6451 GHz, Mod. Amp 4 G. Mw. Power = 2 mW. The (roughly) simulated spectra (red) were obtained with the simulation parameters shown in the insets, using "garlic" (RT) or "pepper" (frozen solution) modules. DFT calculated EPR parameters were used as an initial guess for the simulations.

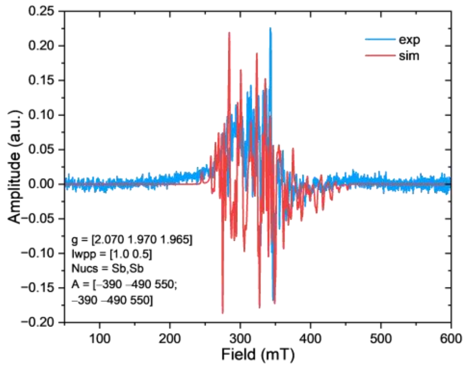

Figure S43. X-band EPR spectrum of [**2**-Sb][B1], recorded in frozen MeTHF at 20K. Mw. Freq=9.6491 GHz, Mod. Amp 4 G. Mw. Power = 20 mW. The (roughly) simulated spectrum (red) was obtained with the simulation parameters shown in the inset, using the "pepper" modules. DFT calculated EPR parameters were used as an initial guess for the simulations.

Table S1. Comparison of the simulated and *DFT calculated* EPR parameters of [**2**-P][B1], [**2**-As][B1] and [**2**-Sb][B1].[a]

	[**2**-P][B1]	[**2**-As][B1]	[**2**-Sb][B1]
**g-tensor 20 K**	2.010, 2.0025, 2.0025	2.047, 2.005, 1.999	2.070, 1.970, 1.965
**g-tensor RT**	2.009, 2.004, 2.004 (t_{corr} = 1.1e^{-10})	2.042, 2.005, 1.999 (t_{corr} = 1.35e^{-10})	/
g-tensor DFT	*2.0075, 2.0019, 2.0006*	*2.0370, 2.0031, 1.9974*	*2.0731, 1.9722, 1.9667*
**A-tensor 20K**	–24, –37, +348; –24, –37, +348	–149, –127, 313; –149, –127, 313	–390, –490, 550; –390, –490, 550
**A-tensor RT**	–10, -5, +348; –10, –5, +348 (t_{corr} = 1.1e^{-10})	[–75, –75, 300; –75, –75, 300] (t_{corr} = 1.35 e^{-10})	/
A-tensor DFT	*–24, –36, +326; –24 –36 +318*	*–130, –148, 233; –133, –152, 241*	*–397, –493, 573; –414, –514, 605*

[a]Simulations using two equivalent P, As and Sb atoms. DFT values based on Turbomole, b3-lyp, rx2c, x2c-TZVPall-2c. The signs of the experimental anisotropic HFC constants (MHz) are based on a combination of comparison with the DFT values and requirements to obtain correct simulations of the isotropic EPR spectra recorded at RT in solution (restricted molecular tumbling model in EasySpin "garlic"). HFC values smaller than 50 MHz are not truly resolved but do contribute to line shape fitting.

Crystallographic Details

Single crystals were examined on a Rigaku Supernova diffractometer using Mo Kα (λ = 0.71073 Å) radiation. The crystals were kept at 100.0(1) K during data collection. Using Olex 2,[11] the structures were solved with the SHELXT[12] ([**2**-P][B1], [**2**-As][B1], [**2**-Sb][B2]) structure solution program using Intrinsic Phasing and refined with the SHELXL[13] ([**2**-P][B1], [**2**-As][B1], [**2**-Sb][B2]) refinement package using Least Squares minimisation. All three compounds contain highly disordered solvent molecules that could not be reliably refined, so solvent masks were calculated.

For [**2**-P][B1] 213 electrons were found in a volume of 1350 $Å^3$ in 4 voids per unit cell. This is consistent with the presence of two toluene solvent molecules per asymmetric unit which account for 200 electrons per unit cell.

For [**2**-As][B1] disorders over two sites of C50, C51 with ratio 67:33, of C108 with ratio 67:33 and of one toluene with ratio 72:28 were found. 229 electrons were found in a volume of 1314 $Å^3$ in 4 voids per unit cell. This is consistent with the presence of 0.5 toluene and two benzene solvent molecules per asymmetric unit which account for 218 electrons per unit cell.

For [**2**-Sb][B2] 100 electrons were found in a volume of 720 $Å^3$ in 4 voids per unit cell. This is consistent with the presence of one toluene solvent molecule per asymmetric unit which account for 100 electrons per unit cell.

Details of the X-ray investigations are given in Table S2. CCDC 2376455 ([**2**-P][B1]), 2376456 ([**2**-As][B1]), and 2376457 ([**2**-Sb][B2]) contain the supplementary crystallographic data for this paper. These data can be obtained free of charge from The Cambridge Crystallographic Data Centre via www.ccdc.cam.ac.uk/structures.

Table S2. Crystallographic details of [**2**-E][B].

	[**2**-P][B1]	[**2**-As][B1]·C_7H_8	[**2**-Sb][B2]
**Empirical formula**	$C_{194}H_{172}B_2F_{40}N_8P_4$	$C_{202.5}H_{179}As_4B_2F_{40}N_8$	$C_{203}H_{188}B_2F_{48}N_8Sb_4$
**Formula weight**	3520.89	3805.83	4160.22
**Temperature [K]**	100.0(1)	100.0(1)	100.0(1)
**Crystal system**	triclinic	triclinic	triclinic
**Space group**	P-1	P-1	P-1
**a [Å]**	18.8332(4)	16.8327(3)	16.9893(4)
**b [Å]**	22.8488(4)	18.6631(3)	24.4470(5)
**c [Å]**	22.8786(5)	29.8456(6)	26.8385(6)
**α [°]**	77.7374(17)	96.4326(16)	66.891(2)
**β [°]**	67.871(2)	96.6223(16)	72.6402(19)
**γ [°]**	80.4721(17)	90.5850(14)	73.6873(18)
**Volume [Å^3]**	8871.9(3)	9252.3(3)	9613.8(4)
Z	2	2	2
ρ_{calc} **[g/cm^3]**	1.318	1.366	1.437
μ **[mm^{-1}]**	0.140	0.812	0.657
**F(000)**	3644.0	3904.0	4216.0
**Crystal size [mm^3]**	0.34 × 0.16 × 0.06	0.29 × 0.2 × 0.13	0.39 × 0.195 × 0.114
**Radiation (λ [Å])**	Mo Kα (λ = 0.71073)	Mo Kα (λ = 0.71073)	Mo Kα (λ = 0.71073)
**2Θ range for data [°]**	6.316 to 60.068	6.592 to 60.068	6.308 to 60.068
**hkl-index ranges**	$-26 \leq h \leq 26$, $-32 \leq k \leq 32$, $-32 \leq l \leq 32$	$-23 \leq h \leq 23$, $-26 \leq k \leq 26$, $-42 \leq l \leq 41$	$-23 \leq h \leq 23$, $-34 \leq k \leq 34$, $-37 \leq l \leq 37$
**Reflections collected**	286569	236407	338860
**Independent reflections**	51840 [R_{int} = 0.0720, R_{sigma} = 0.0636]	54060 [R_{int} = 0.0491, R_{sigma} = 0.0523]	56170 [R_{int} = 0.0664, R_{sigma} = 0.0500]
**Reflections with $I > 2\sigma(I)$**	32883	38993	41052
**Data/restraints/parameters**	51840/0/2139	54060/228/2289	56170/439/2468
***GoF* on F^2**	1.027	1.015	1.029
R_1/wR_2 **[$I > 2\sigma(I)$]**	R_1 = 0.0589, wR_2 = 0.1358	R_1 = 0.0491, wR_2 = 0.1018	R_1 = 0.0465, wR_2 = 0.1028
R_{ind} **[all data]/wR_2**	R_1 = 0.1012, wR_2 = 0.1566	R_1 = 0.0785, wR_2 = 0.1142	R_1 = 0.0725, wR_2 = 0.1154
ρ_{fin} **(max/min) / [e Å^{-3}]**	0.53/−0.42	0.95/−0.55	1.47/−1.05
**CCDC number**	2376455	2376456	2376457

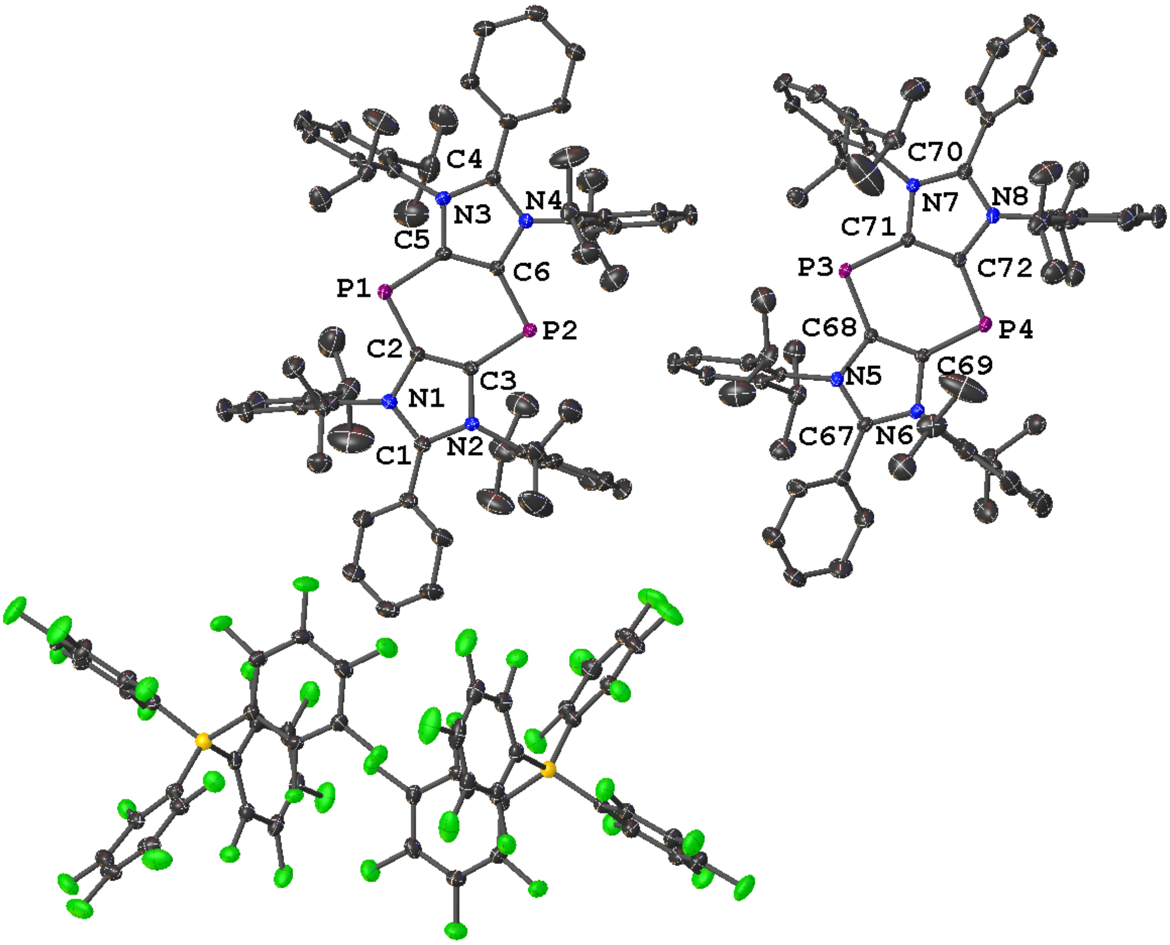

Figure S44. Solid-state molecular structure of $[(ADC)P]_2[B(C_6F_5)_4]$ [**2**-P][B1]. Anisotropic displacement parameters are depicted at the 50 % probability level. Hydrogen atoms are omitted for clarity. Selected bond lengths [Å] and bond angles [°]: *For the first molecule*: P1–C2 1.779(2), P1–C5 1.771(2), P2–C3 1.775(2), P2–C6 1.773(2), C2–C3 1.391(2), C5–C6 1.393(2); C2–C3–P2 132.3(1), C3–C2–P1 132.6(1), C5–C6–P2 133.0(1), C6–C5–P1 132.2(1), C2–P1–C5 95.0(1), C3–P2–C6 94.8(1). Selected Torsion Angles [°]: N1–C2–C3–P2 176.0(2), P1–C2–C3–N2 178.8(2). *For the second molecule*: P3–C68 1.777(2), P3–C71 1.771(2), P4–C69 1.772(2), P4–C72 1.780(2), C68–C69 1.399(2), C71–C72 1.395(2); C68–C69–P4 132.1(1), C69–C68–P3 133.3(1), C71–C72–P4 133.4(1), C72–C71–P3 132.1(1), C68–P3–C71 94.6(1), C69–P4–C72 94.4(1). Selected Torsion Angles [°]: P3–C68–C69–N6 177.1 (1), N5–C68–C69–P4 176.9(1).

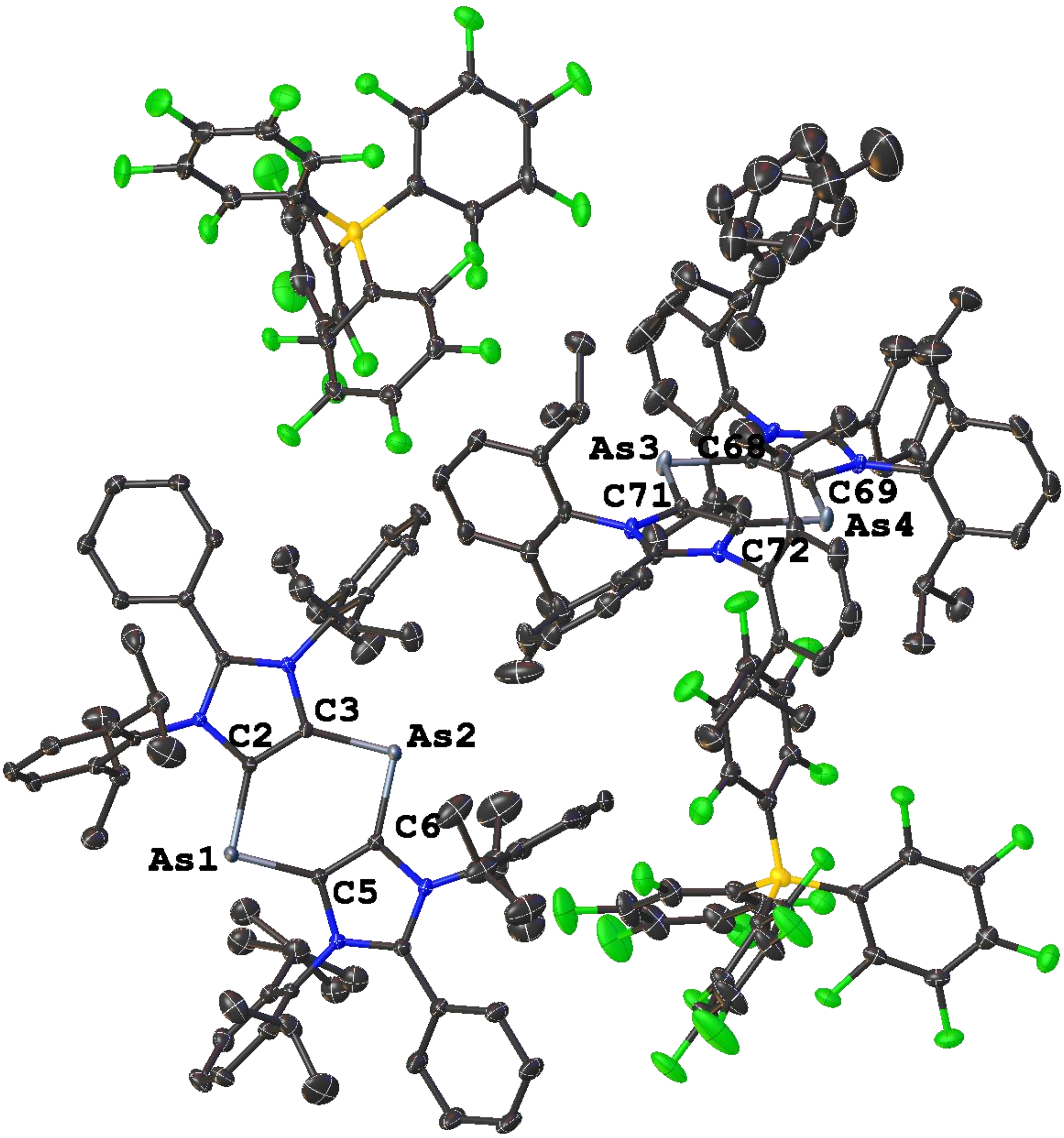

Figure S45. Solid-state molecular structure of $[(ADC)As]_2[B(C_6F_5)_4]$ [**2**-As][B1]. Anisotropic displacement parameters are depicted at the 50 % probability level. Hydrogen atoms are omitted for clarity. Selected bond lengths [Å] and bond angles [°]: *For the first molecule*: As1–C2 1.905(2), As1–C5 1.896(2), As2–C3 1.903(2), As2–C6 1.907(2), C2–C3 1.388(3), C5–C6 1.383(3); C2–C3–As2 133.8(2), C3–C2–As1 133.2(2), C5–C6–As2 133.2(2), C6–C5–As1 133.6(2), C5–As1–C2 92.8(1), C3–As2–C6 92.7(1). Selected Torsion Angles [°]: N1–C2–C3–As2 178.6(2), As1–C2–C3–N2 179.3(2). *For the second molecule*: As3–C68 1.910 (2), As3–C71 1.901(2), As4–C69 1.897(2), As4–C72 1.900(2), C68–C69 1.387(3), C71–C72 1.385(3); C68–C69–As4 133.6(2), C69–C68–As3 133.7(2), C71–C72–As4 134.4(2), C72–C71–As3 133.0(2), C68–As3–C71 92.6(1), C69–As4–C72 92.4(1). Selected Torsion Angles [°]: As3–C68–C69–N6 178.6(2), N5–C68–C69–As4 179.2(2).

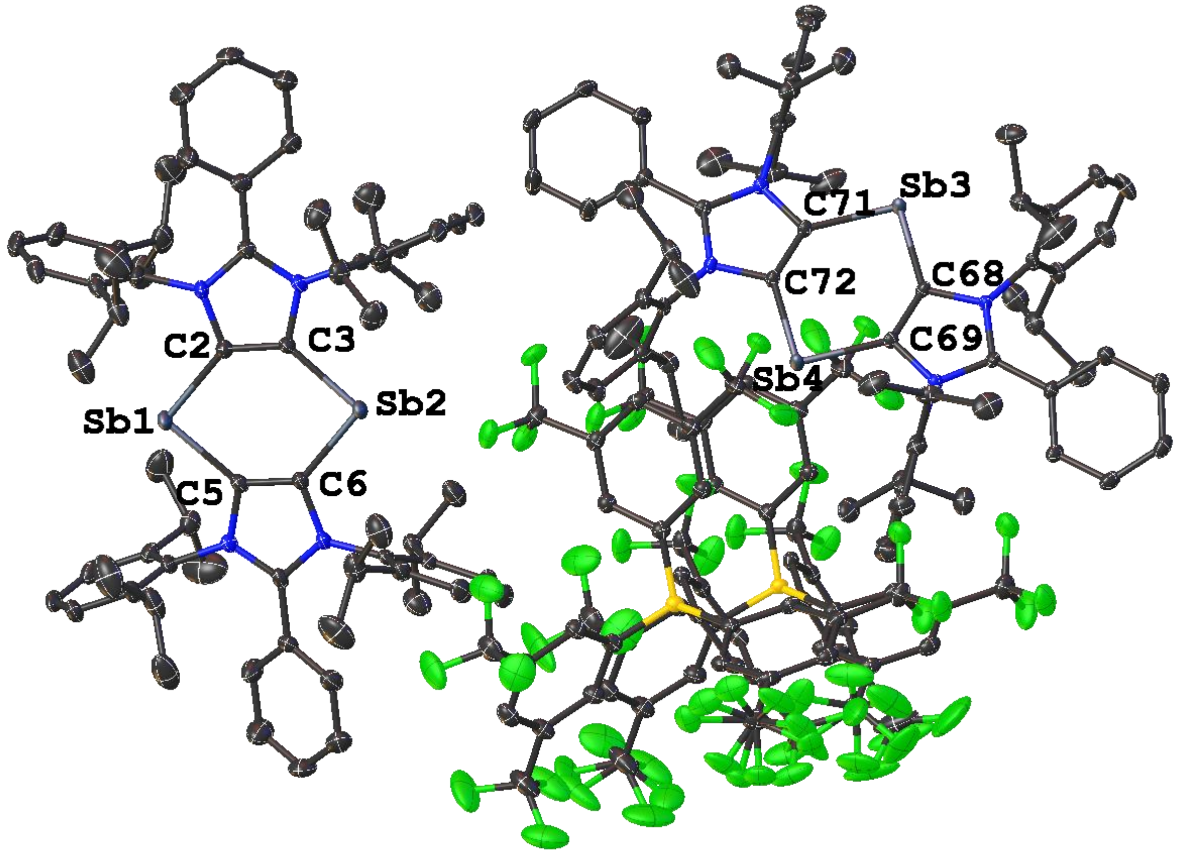

Figure S46. Solid-state molecular structure of $[(ADC)Sb]_2[B\{C_6H_3(CF_3)_2\}_4]$ [**2**-Sb][B2]. Anisotropic displacement parameters are depicted at the 50 % probability level. Hydrogen atoms are omitted for clarity. Just one Selected bond lengths [Å] and bond angles [°]: *For the first molecule*: Sb1–C2 2.126(2), Sb1–C5 2.127(3), Sb2–C3 2.121(2), Sb2–C6 2.117(2), C2–C3 1.386(3), C5–C6 1.387(3); C2–C3– Sb2 135.0(2), C3–C2– Sb1 133.3(2), C5–C6– Sb2 134.3(2), C6–C5–Sb1 134.0(2), C2–Sb1–C5 91.2(1), C6–Sb2–C3 90.8(1). Selected Torsion Angles [°]: N1–C2–C3–Sb2 173.2(2), Sb1–C2–C3–N2 172.5(2). *For the second molecule*: Sb3–C68 2.112 (2), Sb3–C71 2.118(2), Sb4–C69 2.115(2), Sb4–C72 2.117(2), C68–C69 1.389(3), C71–C72 1.381(3); C68–C69–Sb4 134.5(2), C69–C68–Sb3 135.0(2), C71–C72–Sb4 135.1(2), C72–C71–Sb3 134.5(2), C68–Sb3–C71 90.4(1), C69–Sb4–C72 90.4(1). Selected Torsion Angles [°]: Sb3–C68–C69–N6 175.3(2), N5–C68–C69–Sb4 177.6(2).

Computational Details

Molecular structures of the radical cations [**2**-P]$^{\bullet+}$, [**2**-As]$^{\bullet+}$, and [**2**-Sb]$^{\bullet+}$ were optimized at the UKS-PBE0-D3BJ/def2-TZVPP level of theory[14,15,16] implemented in the Orca 5.0.4 software package.[17] For the starting approximations, the respective molecular structures from sc-XRD were taken. The target convergence in geometry optimizations was set to TightOpt. In all Orca calculations, we used the settings TightSCF and DefGrid3, as well as the RIJCOSX accelerating approximation.[18] After optimizations, the structures were additionally symmetrized to remove slight asymmetry due to finite convergence criteria. The optimized molecular structures are shown in Figures S47–S49. Spin density plots are shown in Figures S50–S52.

Natural bond orbital (NBO) analyses[19] were performed using NBO 7.0.10 program.[20] Wavefunctions for these calculations were taken from the described above PBE0-D3BJ/def2-TZVPP geometry optimizations. The results are collected in Table S3.

State-specific complete active space self-consistent field (SS-CASSCF) method were used to investigate the static electron correlation in [**2**-E]$^{\bullet+}$. The def2-TZVP basis sets were utilized in these calculations. As the starting approximation natural orbitals from fc-MP2/def2-SV(P) calculations were taken. Active space of the size (7,6) was used. The results of these calculations (see Table S4) demonstrate a low level of static electron correlation.

Additionally, fractional occupation weighted density (FOD)[21] calculations were performed at the PBE0/def2-TZVPP (T_{el} = 10000 K) level of theory. The resulting N_{FOD} values were 2.40, 2.45 and 2.66 for [**2**-P]$^{\bullet+}$, [**2**-As]$^{\bullet+}$, and [**2**-Sb]$^{\bullet+}$, respectively. These numbers indicate a moderate level of electron correlation. The corresponding FOD plots are shown in Figures S53–S55.

The TD-DFT calculations were performed at the PBE0/def2-TZVPP level using TDA approximation and CPCM solution model of toluene. The selected transitions are listed in Tables S5–S7 and the simulated UV-Vis spectra are shown in Figures S56–S58. Selected frontier molecular orbitals (FMOs) are given in Figures S59–S61.

Table S3. Results of NBO calculations. Natural charges (*q*) and Wiberg bond indices (WBI) and natural spin densities (*s*) are given. For atom numbering see Figures S30–S32.

Parameter	[**2**-P]$^{\bullet+}$	[**2**-As]$^{\bullet+}$	[**2**-Sb]$^{\bullet+}$
q(E1)	0.46	0.49	0.62
q(C8)	−0.18	−0.19	−0.23
q(N3)	−0.33	−0.35	−0.36
q(C7)	0.43	0.43	0.42
s(E1)	0.38	0.40	0.44
s(C8)	−0.01	−0.01	−0.01
s(N3)	−0.02	−0.02	−0.01
s(C7)	0.13	0.11	0.08
WBI(E1–C8)	1.11	1.05	0.93
WBI(C8–C9)	1.34	1.39	1.45
WBI(C8–N3)	1.10	1.09	1.09
WBI(N3–C7)	1.23	1.23	1.24

Table S4. Electronic structure parameters in CASSCF calculations.

Compound	CAS Solution
[**2**-P]$^{\bullet+}$	CAS-SCF STATES FOR BLOCK 1 MULT= 2 NROOTS= 1<br>---<br>ROOT 0: E= −3444.7684559390 Eh<br>0.95548 [125]: 222100<br>0.02112 [100]: 211111<br>0.00545 [114]: 220120<br>0.00524 [88]: 202120<br>0.00523 [112]: 220102<br>0.00515 [86]: 202102
[**2**-As]$^{\bullet+}$	CAS-SCF STATES FOR BLOCK 1 MULT= 2 NROOTS= 1<br>---<br>ROOT 0: E= −7231.6645848925 Eh<br>0.95276 [125]: 222100<br>0.02081 [112]: 220102<br>0.01910 [88]: 202120<br>0.00515 [27]: 022120
[**2**-Sb]$^{\bullet+}$	CAS-SCF STATES FOR BLOCK 1 MULT= 2 NROOTS= 1<br>---<br>ROOT 0: E= −3241.7724945515 Eh<br>0.95563 [125]: 222100<br>0.02125 [100]: 211111<br>0.00543 [114]: 220120<br>0.00529 [112]: 220102<br>0.00525 [88]: 202120<br>0.00523 [86]: 202102

Table S5. Selected transitions (vertical energy differences) in the TD-DFT calculation of [**2**-P]$^{\bullet+}$. Wavelengths λ in nm, oscillator strengths f via transition electric dipole moments and assignments are listed.

λ, nm	f	Assignment
722	0.35	96 % αHOMO → αLUMO
523	0.10	89 % βHOMO → βLUMO
379	0.47	82 % βHOMO−1 → βLUMO
327	0.13	19 % αHOMO−2 → αLUMO+1, 17 % βHOMO−6 → βLUMO, 12 % βHOMO−1 → βLUMO+2
314	0.20	42 % βHOMO → βLUMO+3

Table S6. Selected transitions (vertical energy differences) in the TD-DFT calculation of [**2**-As]$^{\bullet+}$. Wavelengths λ in nm, oscillator strengths f via transition electric dipole moments and assignments are listed.

λ, nm	f	Assignment
694	0.28	96% αHOMO → αLUMO
650	0.12	92% βHOMO → βLUMO
395	0.42	83% βHOMO−1 → βLUMO
335	0.14	25% αHOMO−2 → αLUMO+2, 16 % βHOMO−1 → βLUMO+2
326	0.13	40% βHOMO → βLUMO+3, 12 % βHOMO → βLUMO+5

Table S7. Selected transitions (vertical energy differences) in the TD-DFT calculation of [**2**-Sb]$^{\bullet+}$. Wavelengths λ in nm, oscillator strengths f via transition electric dipole moments and assignments are listed.

λ, nm	f	Assignment
907	0.14	94 % βHOMO → βLUMO
661	0.25	97 % αHOMO → αLUMO
402	0.17	75 % βHOMO−1 → βLUMO
376	0.38	13 % βHOMO−1 → βLUMO, 52 % βHOMO → βLUMO+3, 17 % βHOMO → βLUMO+5

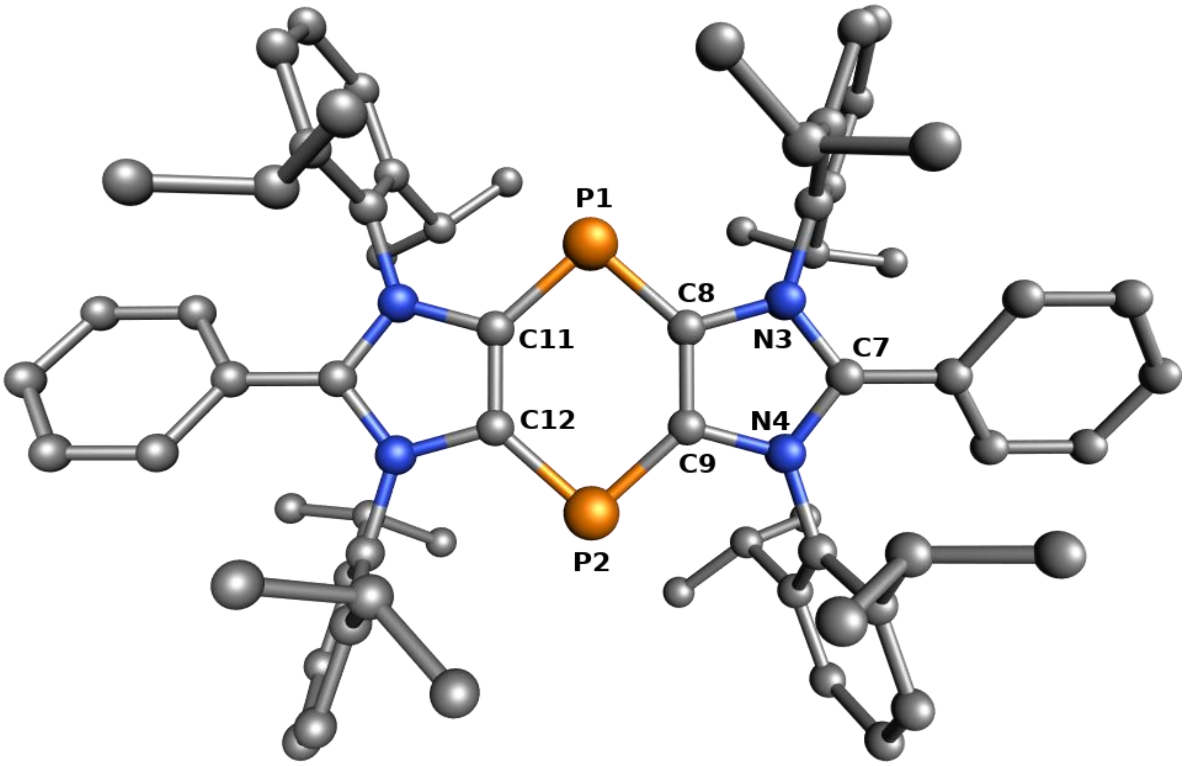

Figure S47. Optimized molecular structure of [**2**-P]$^{\bullet+}$ (D_2 symmetry). Hydrogen atoms are omitted for clarity. Internal working numeration of selected atoms is shown. Selected equilibrium parameters (Å, degrees) are: r(C7-N3)=1.348, r(C7-N4)=1.348, r(C8-P1)=1.770, r(C8-N3)=1.389, r(C9-P2)=1.770, r(C9-N4)=1.389, r(C9-C8)=1.394, r(C11-P1)=1.770, r(C12-P2)=1.770, r(C12-C11)=1.394, a(N3-C7-N4)=106.2, a(C7-N3-C8)=110.9, a(C7-N4-C9)=110.9, a(P1-C8-N3)=121.3, a(P1-C8-C9)=132.7, a(C8-P1-C11)=94.4, a(N3-C8-C9)=105.9, a(P2-C9-N4)=121.3, a(P2-C9-C8)=132.7, a(C9-P2-C12)=94.4, a(N4-C9-C8)=105.9, a(P1-C11-C12)=132.7, a(P2-C12-C11)=132.7, t(C8-N3-C7-N4)= −0.7, t(N3-C7-N4-C9)= −0.7, t(C7-N3-C8-P1)= −176.6, t(C7-N3-C8-C9)=1.9, t(C7-N4-C9-P2)= −176.6, t(C7-N4-C9-C8)=1.9, t(C11-P1-C8-N3)= −179.3, t(P1-C8-C9-P2)= −5.7, t(P1-C8-C9-N4)=176.0, t(C11-P1-C8-C9)=2.6, t(C8-P1-C11-C12)=2.6, t(N3-C8-C9-P2)=176.0, t(N3-C8-C9-N4)= −2.2, t(C12-P2-C9-N4)= −179.3, t(C12-P2-C9-C8)=2.6, t(C9-P2-C12-C11)=2.6, t(P1-C11-C12-P2)= −5.7.

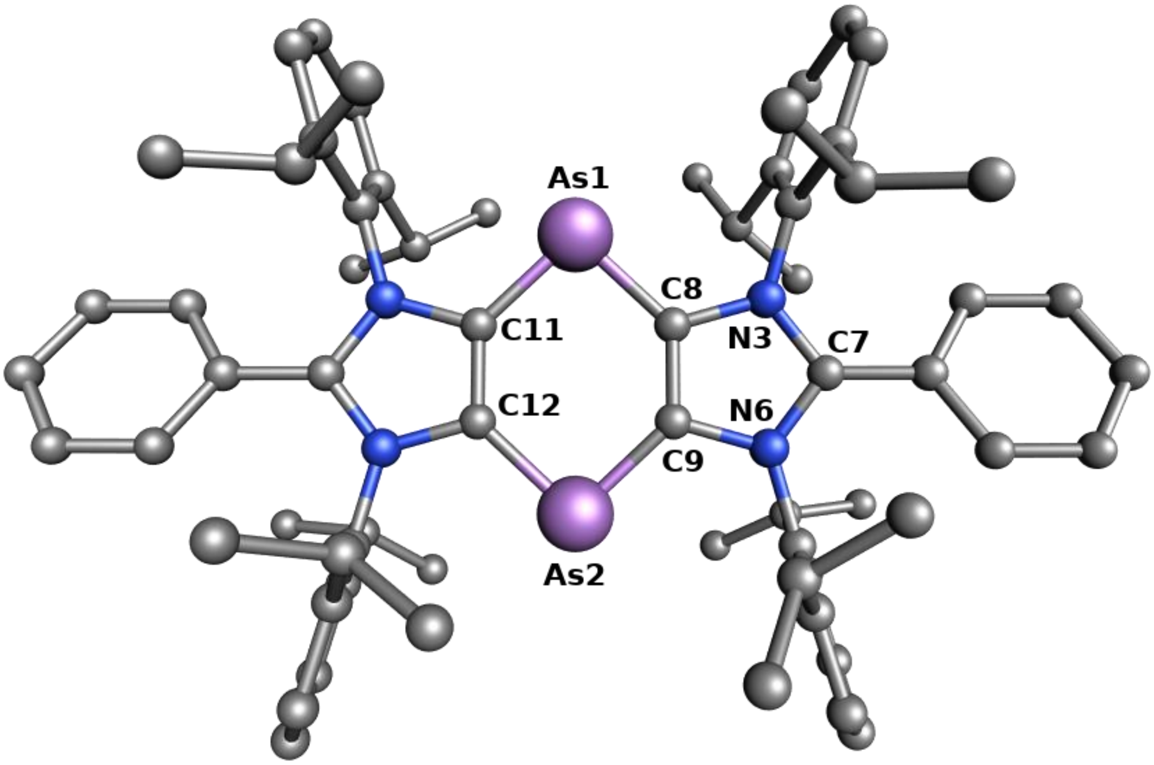

Figure S48. Optimized molecular structure of [**2**-As]$^{\bullet+}$ (C_2 symmetry). Hydrogen atoms are omitted for clarity. Internal working numeration of selected atoms is shown. Selected equilibrium parameters (Å, degrees) are: r(C7-N3)=1.352, r(C7-N6)=1.352, r(C8-As1)=1.900, r(C8-N3)=1.389, r(C9-As2)=1.900, r(C9-N6)=1.389, r(C9-C8)=1.387, r(C11-As1)=1.898, r(C12-As2)=1.898, r(C12-C11)=1.387, a(N3-C7-N6)=105.6, a(C7-N3-C8)=111.1, a(C7-N6-C9)=111.1, a(As1-C8-N3)=120.4, a(As1-C8-C9)=133.6, a(C8-As1-C11)=92.7, a(N3-C8-C9)=106.0, a(As2-C9-N6)=120.4, a(As2-C9-C8)=133.6, a(C9-As2-C12)=92.7, a(N6-C9-C8)=106.0, a(As1-C11-C12)=133.6, a(As2-C12-C11)=133.6, t(C8-N3-C7-N6)=0.7, t(N3-C7-N6-C9)=0.7, t(C7-N3-C8-As1)=177.6, t(C7-N3-C8-C9)= −1.7, t(C7-N6-C9-As2)=177.6, t(C7-N6-C9-C8)= −1.7, t(C11-As1-C8-N3)=177.8, t(As1-C8-C9-As2)=3.7, t(As1-C8-C9-N6)= −177.1, t(C11-As1-C8-C9)= −3.2, t(C8-As1-C11-C12)=5.0, t(N3-C8-C9-As2)= −177.1, t(N3-C8-C9-N6)=2.0, t(C12-As2-C9-N6)=177.8, t(C12-As2-C9-C8)= −3.2, t(C9-As2-C12-C11)=5.0, t(As1-C11-C12-As2)= −7.7.

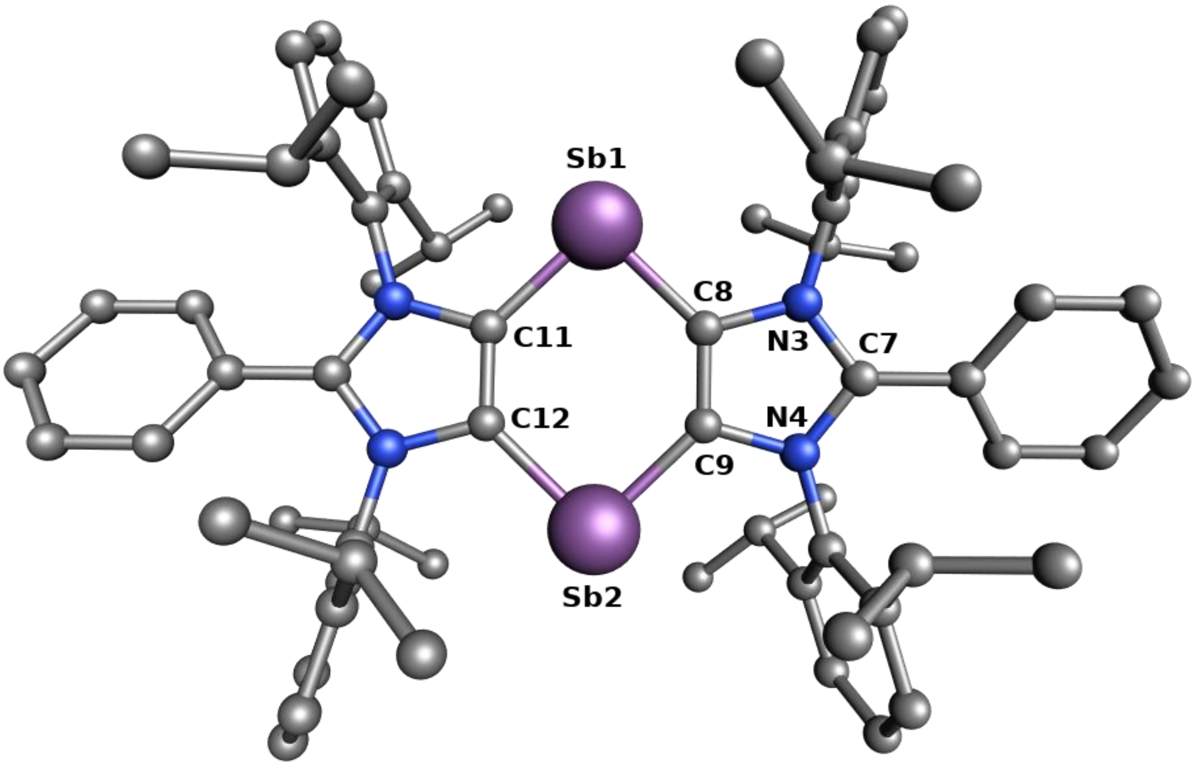

Figure S49. Optimized molecular structure of free [**2**-Sb]$^{\bullet+}$ (D_2 symmetry). Hydrogen atoms are omitted for clarity. Internal working numeration of selected atoms is shown. Selected equilibrium parameters (Å, degrees) are: r(C7-N3)=1.347, r(C7-N4)=1.347, r(C8-Sb1)=2.112, r(C8-N3)=1.391, r(C9-Sb2)=2.112, r(C9-N4)=1.391, r(C9-C8)=1.384, r(C11-Sb1)=2.112, r(C12-Sb2)=2.112, r(C12-C11)=1.384, a(N3-C7-N4)=105.6, a(C7-N3-C8)=111.3, a(C7-N4-C9)=111.3, a(Sb1-C8-N3)=119.4, a(Sb1-C8-C9)=134.5, a(C8-Sb1-C11)=90.4, a(N3-C8-C9)=105.9, a(Sb2-C9-N4)=119.4, a(Sb2-C9-C8)=134.5, a(C9-Sb2-C12)=90.4, a(N4-C9-C8)=105.9, a(Sb1-C11-C12)=134.5, a(Sb2-C12-C11)=134.5, t(C8-N3-C7-N4)= −0.9, t(N3-C7-N4-C9)= −0.9, t(C7-N3-C8-Sb1)= −174.0, t(C7-N3-C8-C9)=2.2, t(C7-N4-C9-Sb2)= −174.0, t(C7-N4-C9-C8)=2.2, t(C11-Sb1-C8-N3)= −179.4, t(Sb1-C8-C9-Sb2)= −11.9, t(Sb1-C8-C9-N4)=172.7, t(C11-Sb1-C8-C9)=5.8, t(C8-Sb1-C11-C12)=5.8, t(N3-C8-C9-Sb2)=172.7, t(N3-C8-C9-N4)= −2.6, t(C12-Sb2-C9-N4)= −179.4, t(C12-Sb2-C9-C8)=5.8, t(C9-Sb2-C12-C11)=5.8, t(Sb1-C11-C12-Sb2)= −11.9.

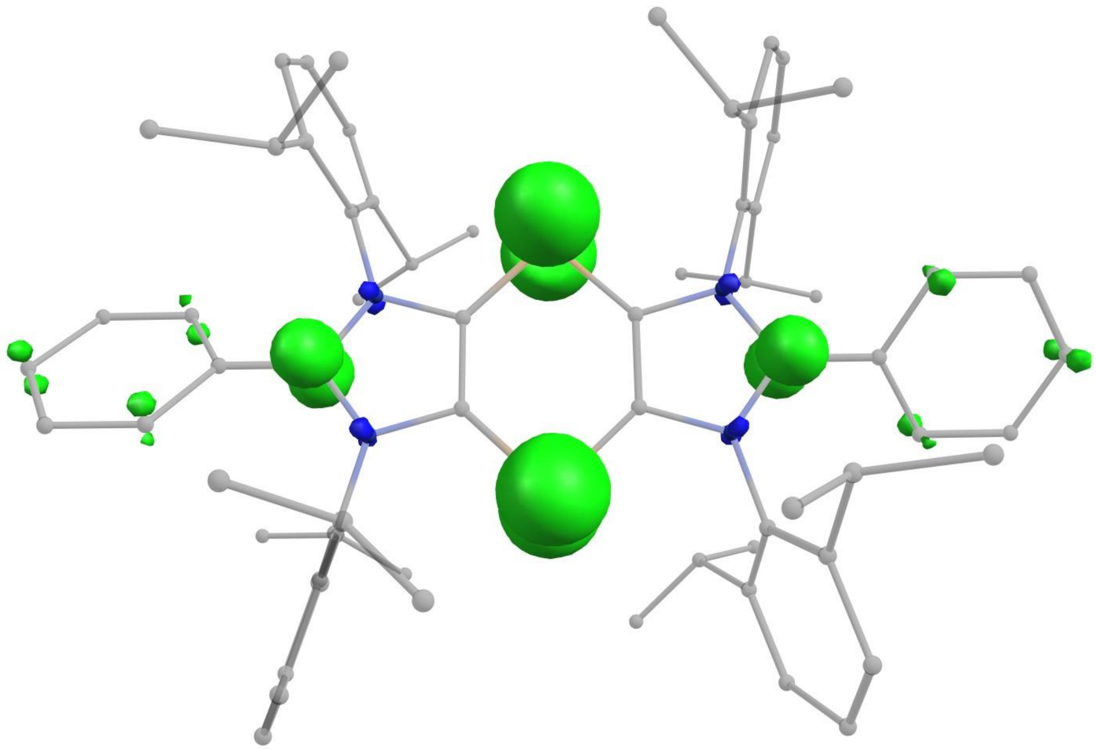

Figure S50. Spin density (isosurfaces 0.005 a.e.) of [**2**-P]$^{\bullet+}$ in PBE0-D3BJ/def2-TZVPP calculation. Hydrogen atoms are omitted for clarity.

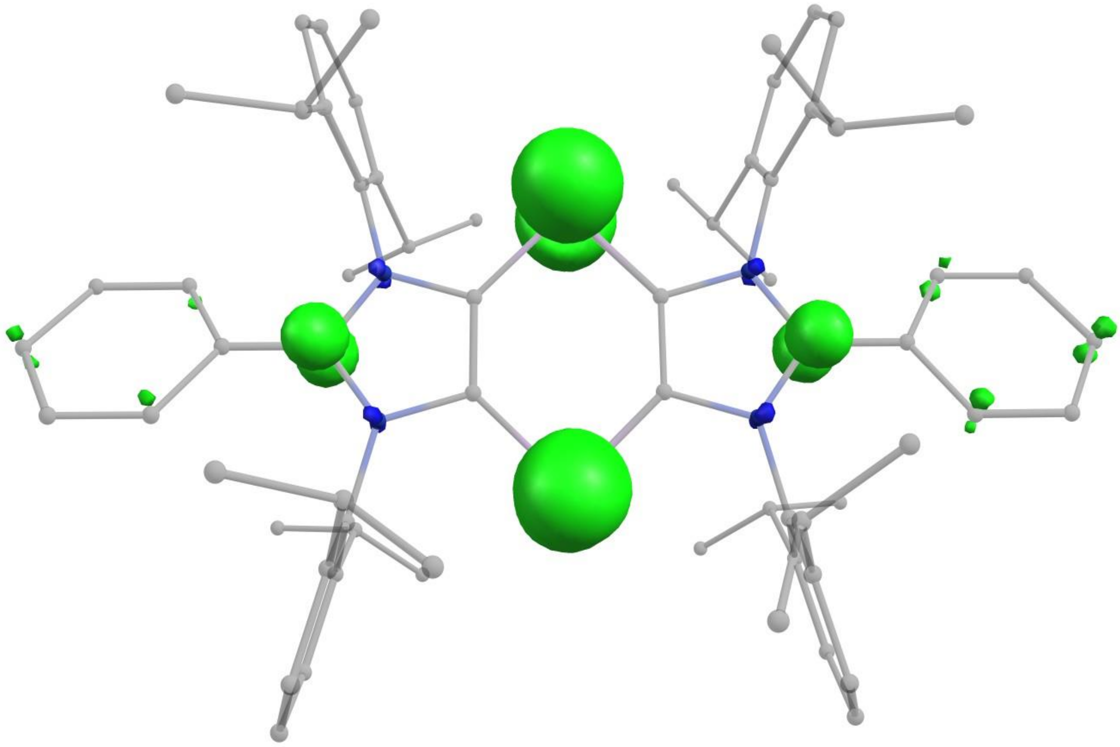

Figure S51. Spin density (isosurfaces 0.005 a.e.) of [**2**-As]$^{\bullet+}$ in PBE0-D3BJ/def2-TZVPP calculation. Hydrogen atoms are omitted for clarity.

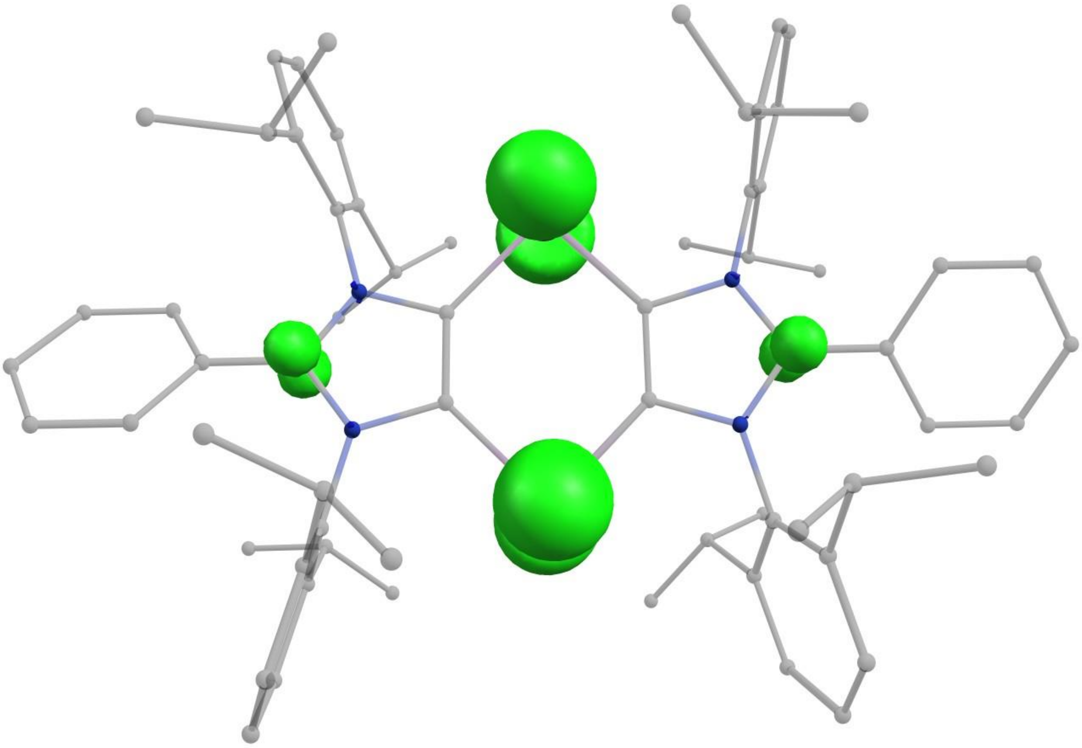

Figure S52. Spin density (isosurfaces 0.005 a.e.) of [**2**-Sb]$^{\bullet+}$ in PBE0-D3BJ/def2-TZVPP calculation. Hydrogen atoms are omitted for clarity.

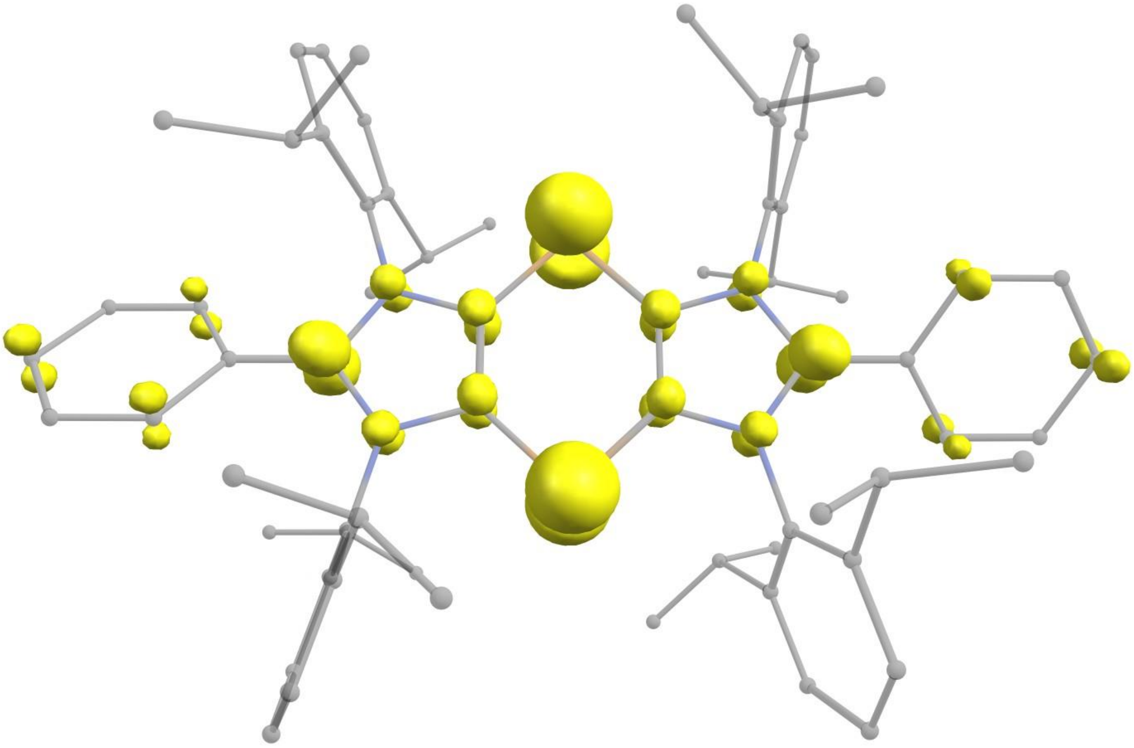

Figure S53. FOD plot (isosurfaces 0.005 a.e. in yellow) of [**2**-P]$^{\bullet+}$. Hydrogen atoms are omitted for clarity.

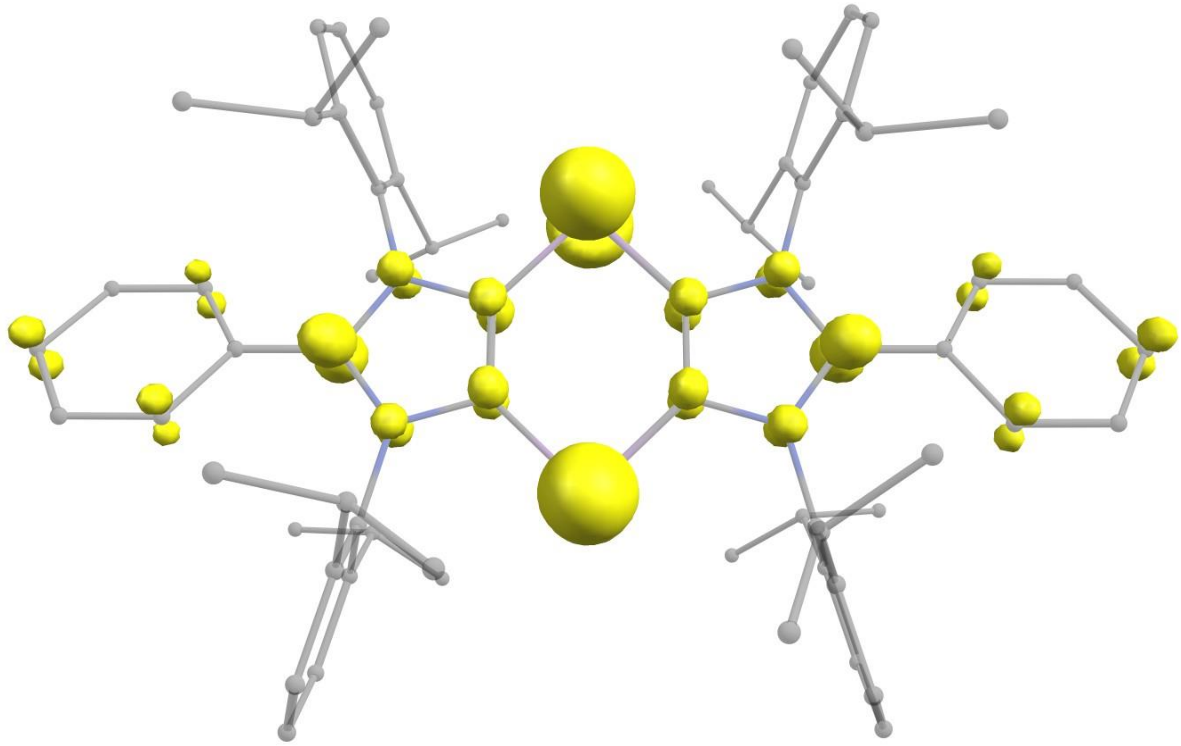

Figure S54. FOD plot (isosurfaces 0.005 a.e. in yellow) of [**2**-As]$^{\bullet+}$. Hydrogen atoms are omitted for clarity.

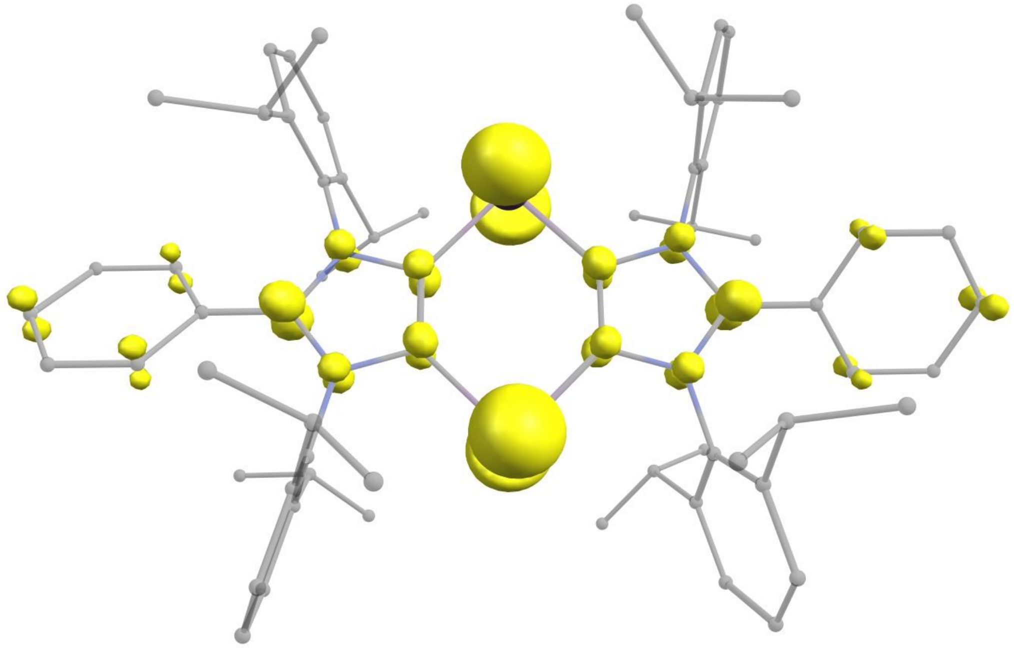

Figure S55. FOD plot (isosurfaces 0.005 a.e. in yellow) of [**2**-Sb]$^{\bullet+}$. Hydrogen atoms are omitted for clarity.

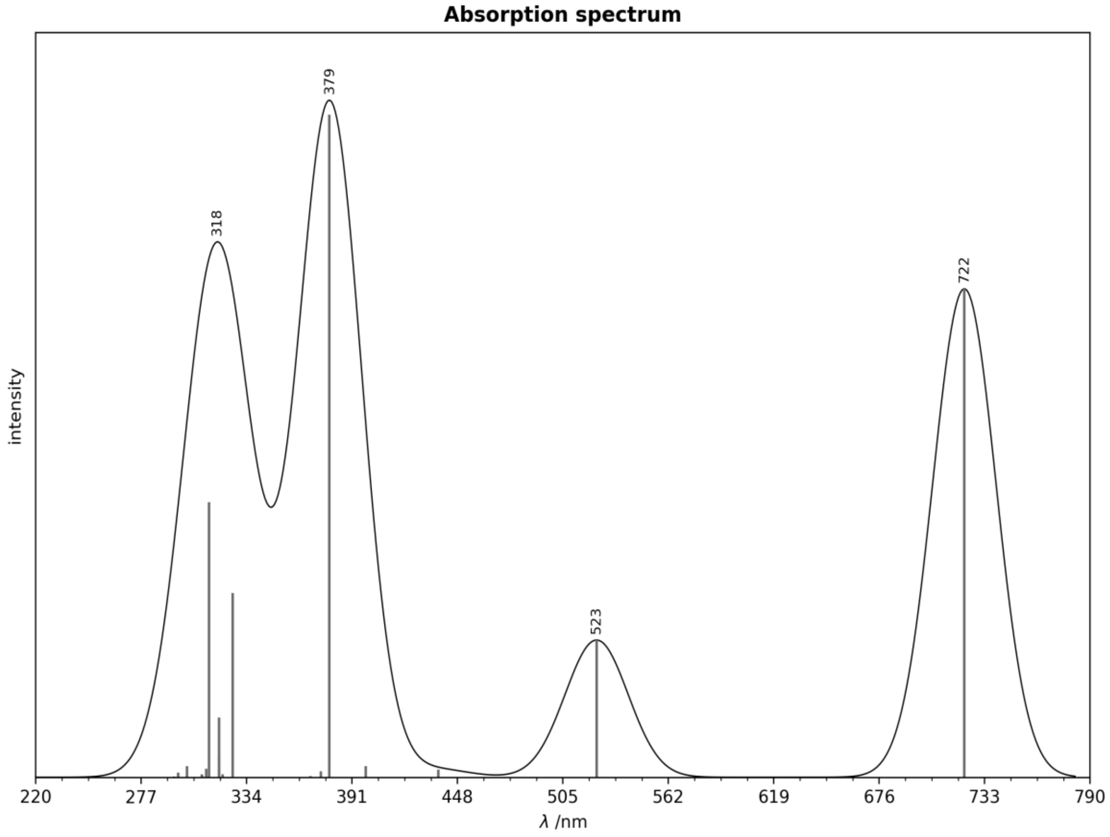

Figure S56. Simulated UV-Vis spectrum of [**2**-P]$^{\bullet+}$ based on TD-DFT calculation at the PBE0/def2-TZVPP level of theory with CPCM(Toluene). Note, the number of calculated roots was restricted to 40. No empirical shift has been applied to the transition energies.

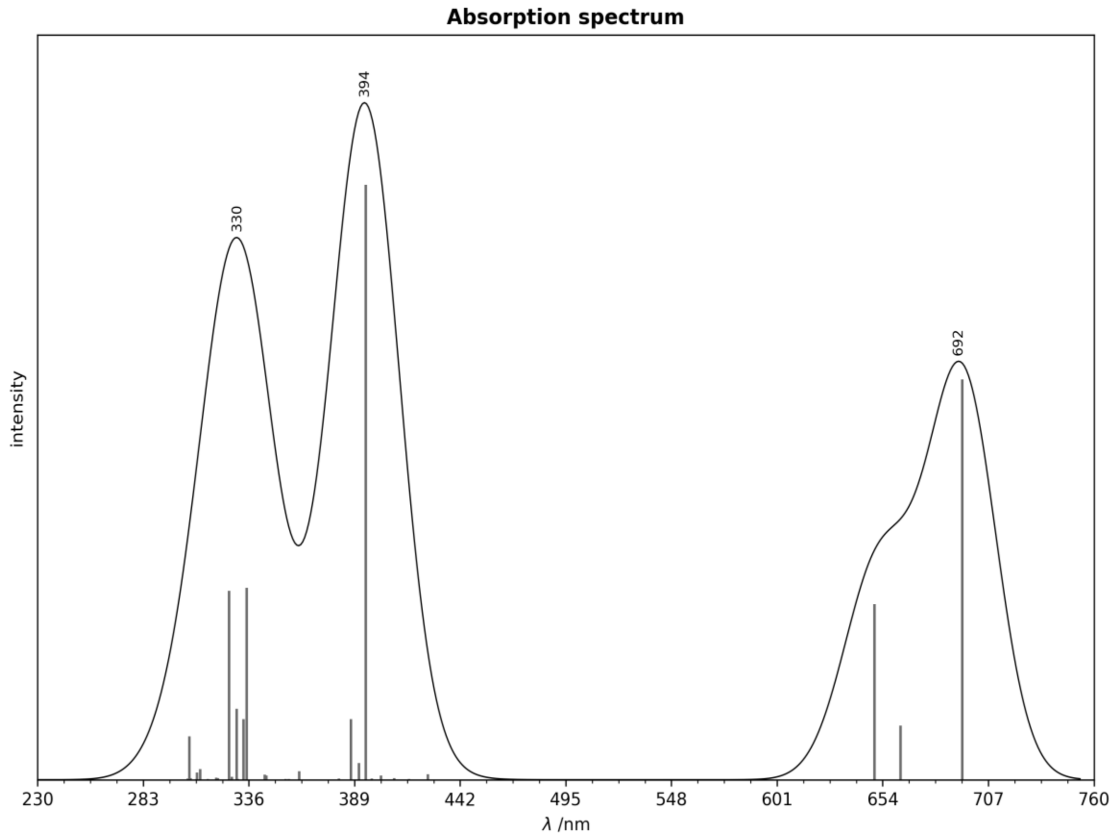

Figure S57. Simulated UV-Vis spectrum of [**2**-As]$^{\bullet+}$ based on TD-DFT calculation at the PBE0/def2-TZVPP level of theory with CPCM(Toluene). Note, the number of calculated roots was restricted to 40. No empirical shift has been applied to the transition energies.

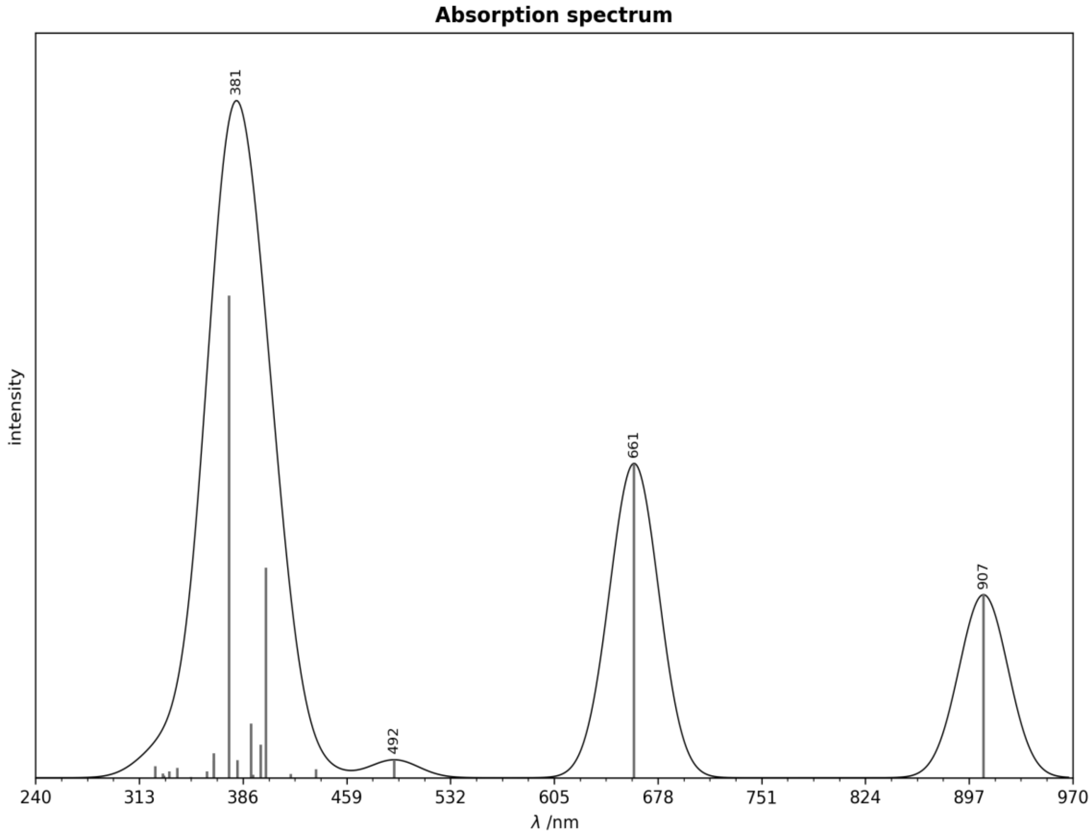

Figure S58. Simulated UV-Vis spectrum of [**2**-Sb]$^{\bullet+}$ based on TD-DFT calculation at the PBE0/def2-TZVPP level of theory with CPCM(Toluene). Note, the number of calculated roots was restricted to 40. No empirical shift has been applied to the transition energies.

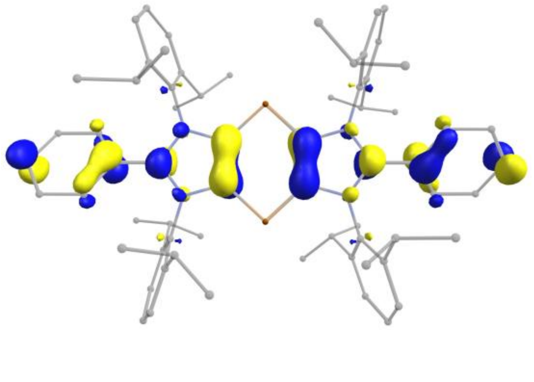

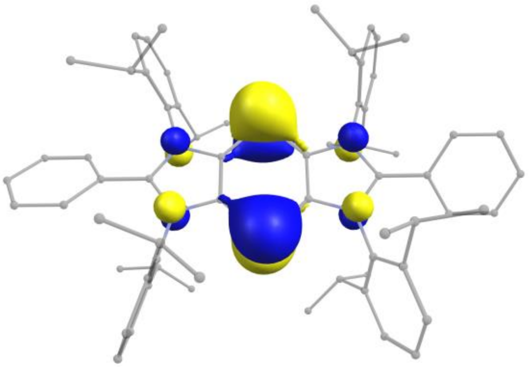

αHOMO−2 (−7.8404)

αHOMO−1 (−7.5127)

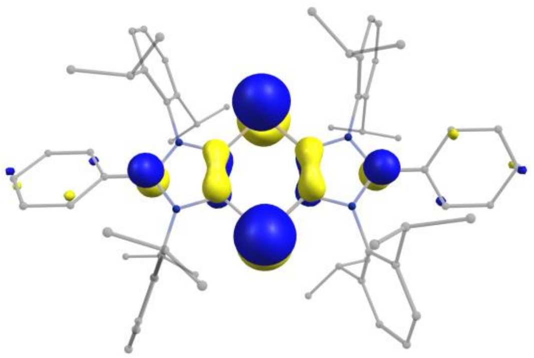

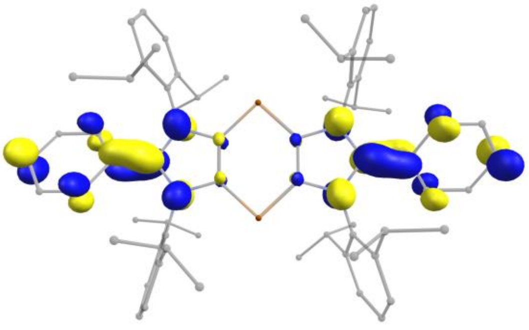

αHOMO (−5.1765)

αLUMO (−2.6931)

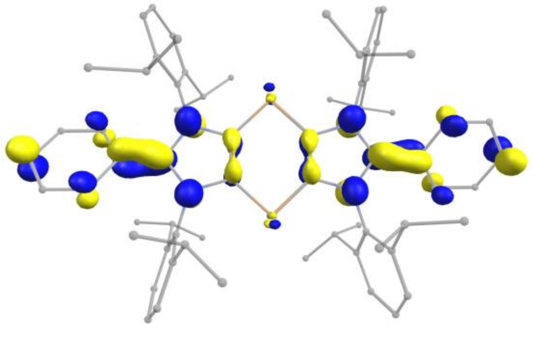

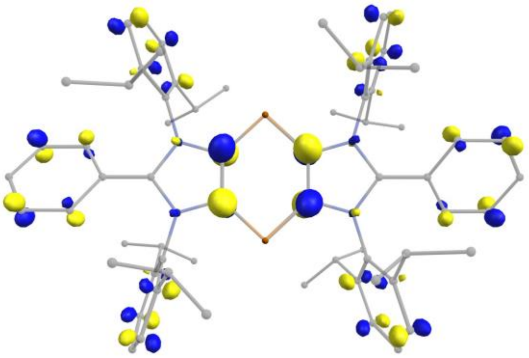

αLUMO+1 (−2.6074)

αLUMO+2 (−1.7559)

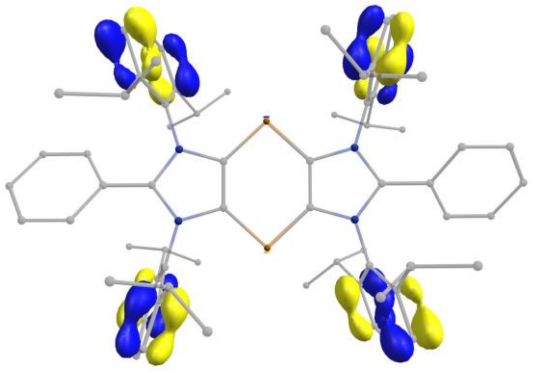

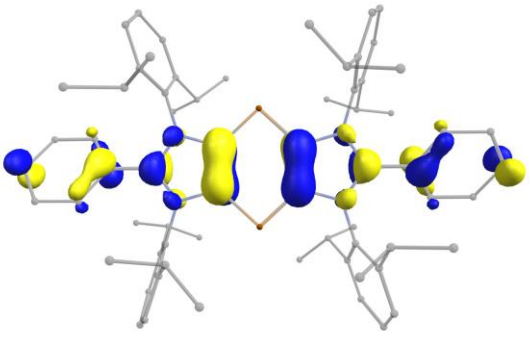

βHOMO−2 (−7.9915)

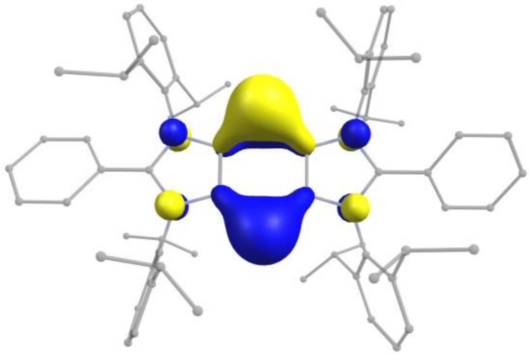

βHOMO−1 (−7.7133)

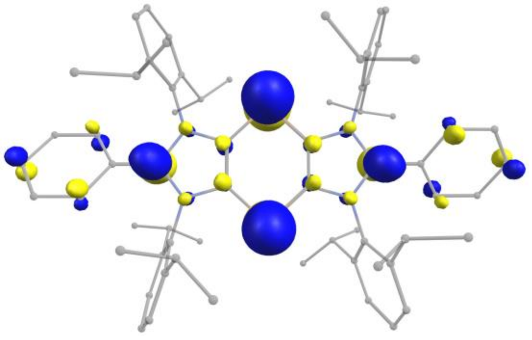

βHOMO (−6.8279)

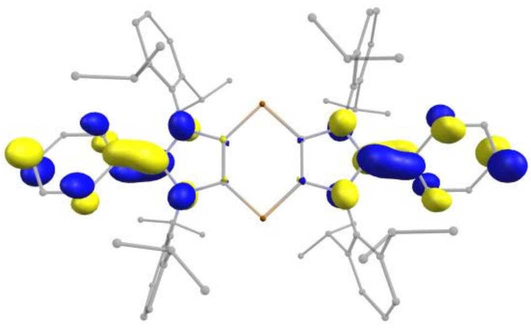

βLUMO (−3.4528)

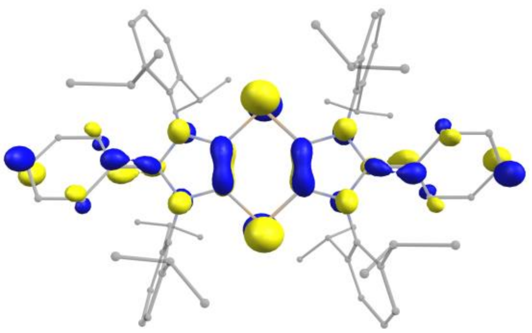

βLUMO+1 (−2.5011)

βLUMO+2 (−2.2708)

Figure S59. Frontier molecular orbitals (isosurfaces 0.05 a.u.) and respective energies (eV) in PBE0/def2-TZVPP CPCM(Toluene) calculation of [**2**-P]$^{\bullet+}$.

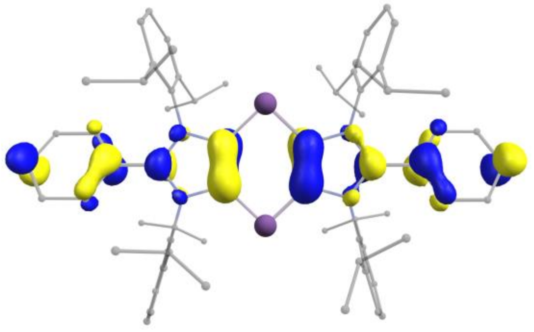

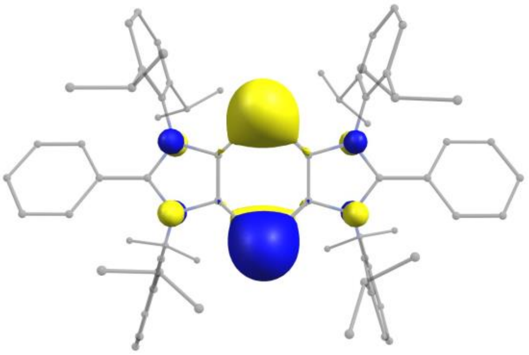

αHOMO−2 (−7.8232) **αHOMO−1 (−7.1225)**

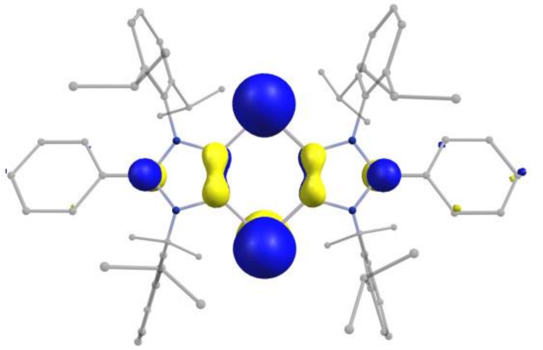

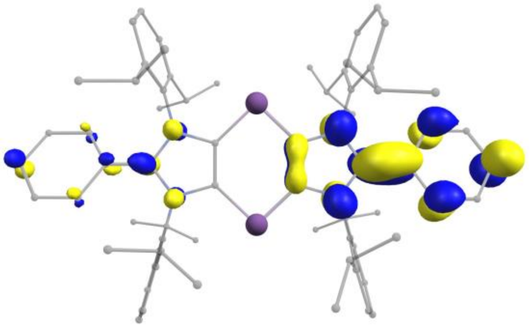

αHOMO (−5.2654) **αLUMO (−2.7136)**

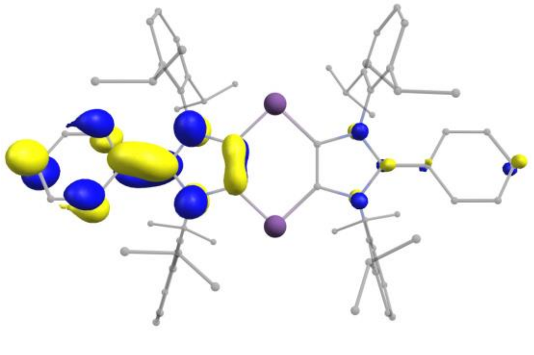

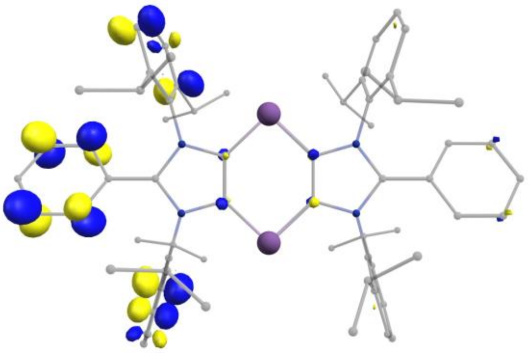

αLUMO+1 (−2.6184) **αLUMO+2 (−1.7157)**

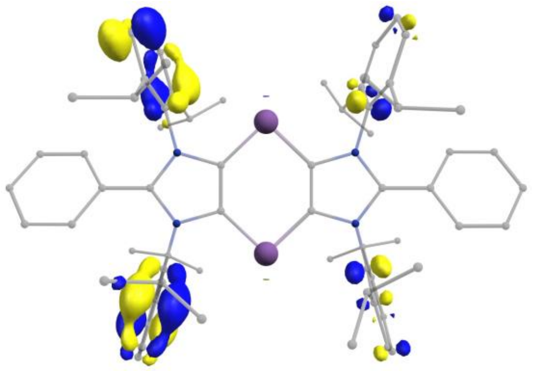

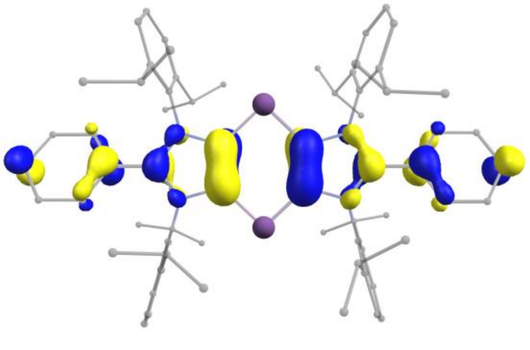

βHOMO−2 (−8.0069) **βHOMO−1 (−7.6983)**

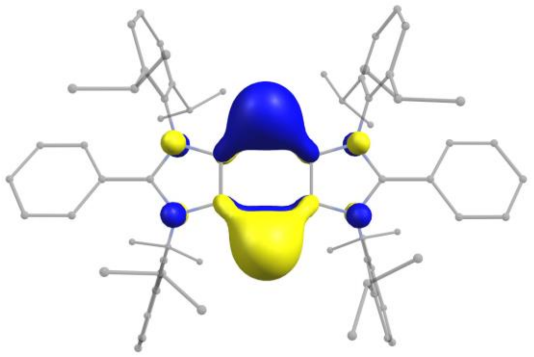

βHOMO (−6.3976)

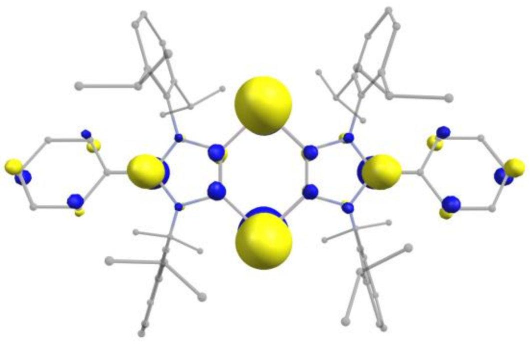

βLUMO (−3.5958)

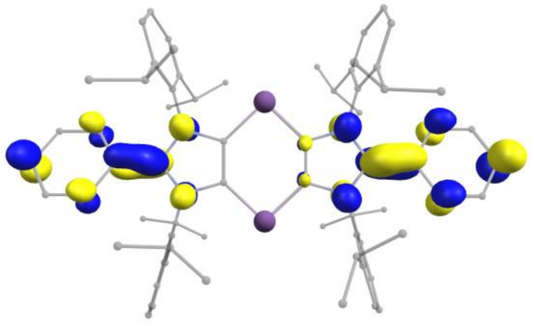

βLUMO+1 (−2.5424)

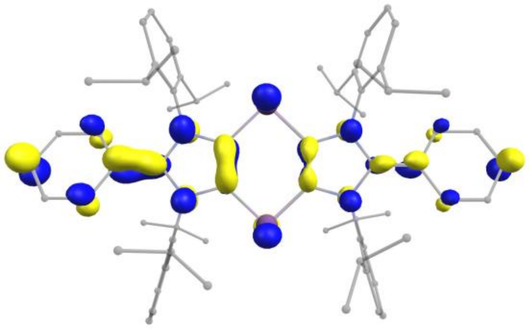

βLUMO+2 (−2.3466)

Figure S60. Frontier molecular orbitals (isosurfaces 0.05 a.u.) and respective energies (eV) in PBE0/def2-TZVPP CPCM(Toluene) calculation of [**2**-As]$^{\bullet+}$.

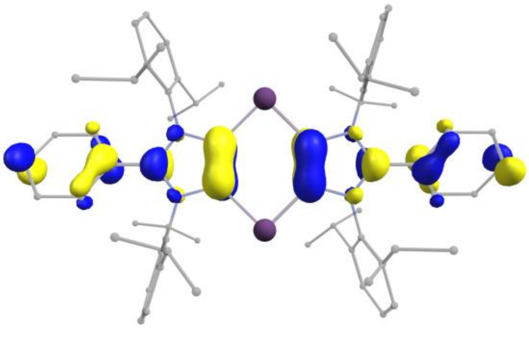

αHOMO−2 (−7.7465)

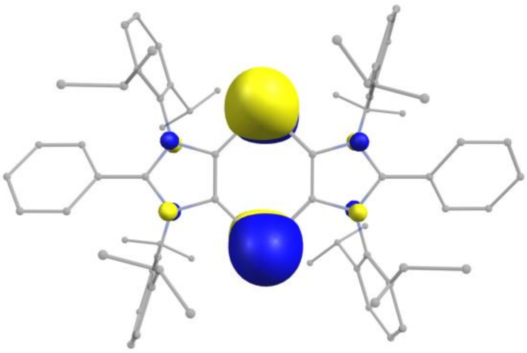

αHOMO−1 (−6.5015)

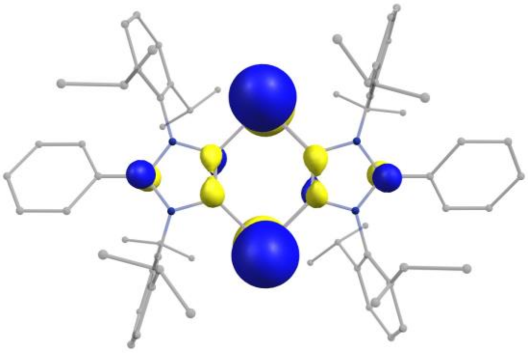

αHOMO (−5.2217)

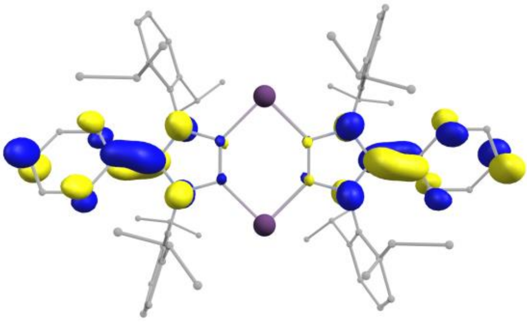

αLUMO (−2.6072)

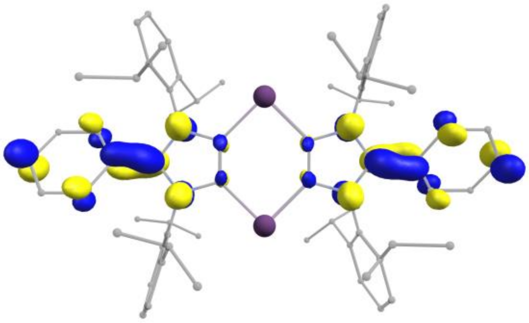

αLUMO+1 (−2.5825)

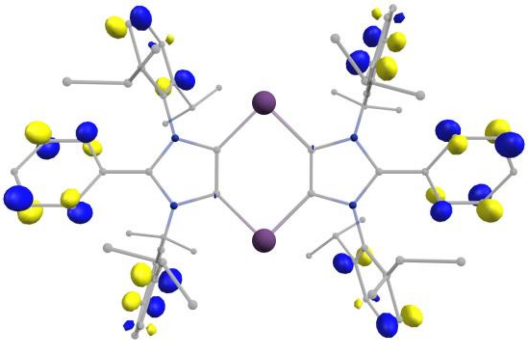

αLUMO+2 (−1.7067)

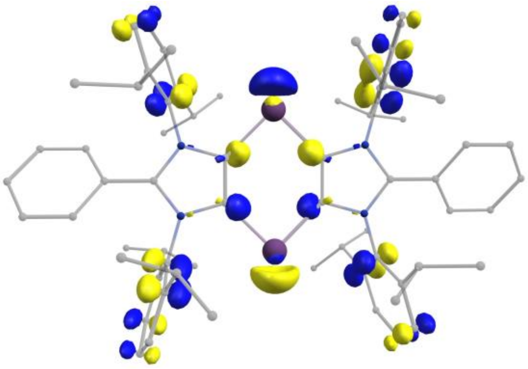

βHOMO−2 (−7.9397)

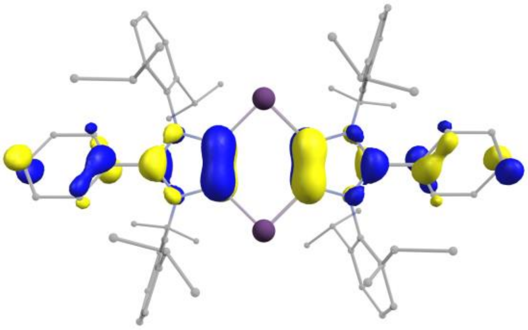

βHOMO−1 (−7.6445)

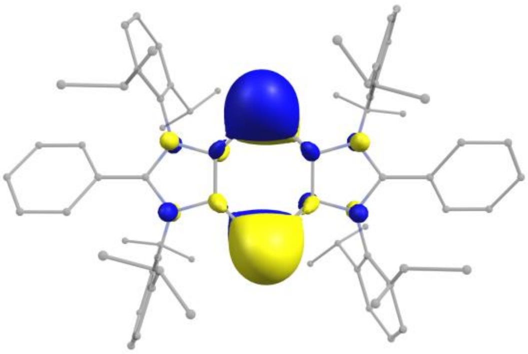

βHOMO (−5.7428)

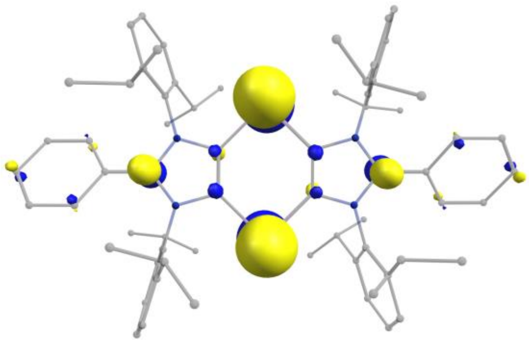

βLUMO (−3.6124)

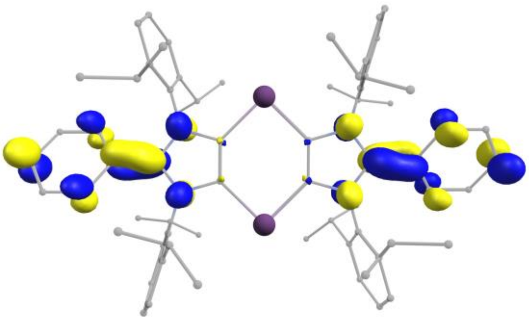

βLUMO+1 (−2.4936)

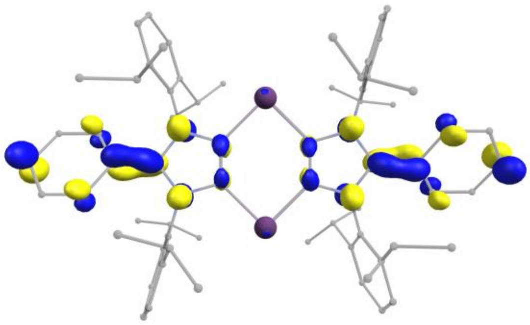

βLUMO+2 (−2.3880)

Figure S61. Frontier molecular orbitals (isosurfaces 0.05 a.u.) and respective energies (eV) in PBE0/def2-TZVPP CPCM(Toluene) calculation of [**2**-Sb]$^{\bullet+}$.

References

[1] G. R. Fulmer, A. J. M. Miller, N. H. Sherden, H. E. Gottlieb, A. Nudelman, B. M. Stoltz, J. E. Bercaw, K. I. Goldberg, *Organometallics* **2010**, *29*, 2176–2179.

[2] N. K. T. Ho, B. Neumann, H.-G. Stammler, V. H. Da Menezes Silva, D. G. Watanabe, A. A. C. Braga, R. S. Ghadwal, *Dalton Trans*. **2017**, *46*, 12027–12031.

[3] A. J. Sicard, R. T. Baker, *Org. Process Res. Dev.* **2020**, *24*, 12, 2950–2952.

[4] H. Podall, W. E. Foster, and A. P. Giraitis, *J. Org. Chem.* **1958**, *23*, 82–85.

[5] J. C. W. Chien, W. M. Tsai, M. D. Rausch, *J. Am. Chem. Soc.* **1991**, *113*, 8570–8571.

[6] A. J. Martínez-Martínez, A. S. Weller, *Dalton Trans.* **2019**, *48*, 3551–3554.

[7] For **1**-P: D. Rottschäfer, B. Neumann, H.-G. Stammler, T. Sergeieva, D. M. Andrada, R. S. Ghadwal, *Chemistry* **2021**, *27*, 3055–3064. For **1**-As: D. Rottschäfer, T. Glodde, B. Neumann, H.-G. Stammler, D. M. Andrada, R. S. Ghadwal, *Angew. Chem. Int. Ed.* **2021**, *60*, 15849–15853. For **1**-Sb: H. Steffenfauseweh, D. Rottschäfer, Y. V. Vishnevskiy, B. Neumann, H.-G. Stammler, D. W. Szczepanik, R. S. Ghadwal, *Angew. Chem. Int. Ed.* **2023**, *62*, e202216003.

[8] Stoll, S.; Schweiger, A. EasySpin, a Comprehensive Software Package for Spectral Simulation and Analysis in EPR. *J. Magn. Reson.* **2006**, *178* (1), 42–55. DOI: 10.1016/j.jmr.2005.08.013.

[9] *cwEPR – MATLAB Central File Exchange*. https://www.mathworks.com/matlabcentral/fileexchange/73292–cwepr

[10] R. Ahlrichs, Turbomole Version 7.8.1, Theoretical Chemistry Group, University of Karlsruhe.

[11] O.V. Dolomanov, L. J. Bourhis, R. J. Gildea, J. A. K. Howard, H. Puschmann, *J. Appl. 1. Cryst* **2009**, *42*, 339–341.

[12] G. M. Sheldrick, *Acta Cryst. A* **2015**, *71*, 3–8.

[13] G. M. Sheldrick, *Acta Cryst. C* **2015**, *71*, 3–8.

[14] C. Adamo, V. Barone, *J. Chem. Phys.* **1999**, *110*, 6158–6170.

[15] S. Grimme, S. Ehrlich, L. Goerigk, *J. Comput. Chem.* **2011**, *32*, 1456–1465.

[16] F. Weigend, R. Ahlrichs, *Phys. Chem. Chem. Phys.* **2005**, *7*, 3297–3305.

[17] F. Neese, *Wiley Interdiscip. Rev. Comput. Mol. Sci.* **2022**, *12*, e1606.

[18] F. Neese, F. Wennmohs, A. Hansen, U. Becker, *Chem. Phys.* **2009**, *356*, 98–109.

[19] F. Weinhold and C. R. Landis, *Valency and Bonding: A Natural Bond Orbital Donor-Acceptor Perspective* (Cambridge University Press, 2005), 760pp.

[20] NBO 7.0. E. D. Glendening, J. K. Badenhoop, A. E. Reed, J. E. Carpenter, J. A. Bohmann, C. M. Morales, P. Karafiloglou, C. R. Landis, and F. Weinhold, Theoretical Chemistry Institute, University of Wisconsin, Madison, WI (2018)

[21] C. A. Bauer, A. Hansen, S. Grimme, *Chem. Eur. J.* **2017**, *23*, 6150–6164.
